# Direct attenuation of *Arabidopsis* ERECTA signalling by a pair of U-box E3 ligases

**DOI:** 10.1038/s41477-022-01303-x

**Published:** 2022-12-20

**Authors:** Liangliang Chen, Alicia M. Cochran, Jessica M. Waite, Ken Shirasu, Shannon M. Bemis, Keiko U. Torii

**Affiliations:** 1 Howard Hughes Medical Institute and Department of Molecular Biosciences, The University of Texas at Austin, Austin, TX USA; 2 Department of Biology, University of Washington, Seattle, WA USA; 3 RIKEN Center for Sustainable Resource Science, Yokohama, Japan; 4 Present Address: USDA-ARS Tree Fruit Research Laboratory, Wenatchee, WA USA

**Keywords:** Plant signalling, Plant morphogenesis, Stomata

## Abstract

Plants sense a myriad of signals through cell-surface receptors to coordinate their development and environmental response. The *Arabidopsis* ERECTA receptor kinase regulates diverse developmental processes via perceiving multiple EPIDERMAL PATTERNING FACTOR (EPF)/EPF-LIKE peptide ligands. How the activated ERECTA protein is turned over is unknown. Here we identify two closely related plant U-box ubiquitin E3 ligases, PUB30 and PUB31, as key attenuators of ERECTA signalling for two developmental processes: inflorescence/pedicel growth and stomatal development. Loss-of-function *pub30 pub31* mutant plants exhibit extreme inflorescence/pedicel elongation and reduced stomatal numbers owing to excessive ERECTA protein accumulation. Ligand activation of ERECTA leads to phosphorylation of PUB30/31 via BRI1-ASSOCIATED KINASE1 (BAK1), which acts as a coreceptor kinase and a scaffold to promote PUB30/31 to associate with and ubiquitinate ERECTA for eventual degradation. Our work highlights PUB30 and PUB31 as integral components of the ERECTA regulatory circuit that ensure optimal signalling outputs, thereby defining the role for PUB proteins in developmental signalling.

## Abstract (web summary)

PUB30/31 ensure the optimal signalling output of ERECTA for proper plant growth and stomatal development. A heterodimeric partner BAK1 is a kinase and a scaffold that activates PUB30/31, which in turn ubiquitinate ligand-activated ERECTA for degradation.

## Main

The development of multicellular organisms relies on coordinated cell proliferation and differentiation in response to external cues. Plants use a battery of membrane-bound cell-surface receptors with an intracellular kinase domain, collectively known as receptor-like kinases (RLKs), to sense and transduce external signals to adjust cellular responses^1^. Among them, those with an extracellular leucine-rich repeat (LRR) domain, LRR-RLKs, comprise the largest subfamily in plants with more than 200 members in *Arabidopsis*^1^. The LRR-RLKs play critical roles in development, hormone perception, interkingdom communication and immunity. Those with known ligands are referred to as LRR-RKs hereafter^2,3^. Well-studied LRR-RKs include the brassinosteroid receptor BRASSINOSTEROID INSENSITIVE1 (BRI1)^4^ and the immune receptor FLAGELLIN SENSING2 (FLS2)^5,6^, among others^2,3^.

The *Arabidopsis* ERECTA LRR-RK regulates diverse aspects of plant development, including inflorescence architecture, stem and pedicel elongation, flower development, vascular differentiation and stomatal patterning^7–12^. ERECTA perceives multiple peptide ligands, all belonging to the EPIDERMAL PATTERNING FACTOR (EPF)/EPF-LIKE (EPFL) family^13^. Previous studies have identified both unique and shared components of ERECTA signalling pathways. For example, among EPF/EPFL signalling peptides, EPFL6 (also known as CHALLAH) and EPFL4 from the stem endodermis are perceived by ERECTA and promote cell proliferation and stem/pedicel elongation for proper inflorescence architecture^14–16^. On the other hand, during stomatal development, EPF2 is primarily perceived by ERECTA to inhibit the stomatal lineage entry divisions^17–19^. The receptor-like protein TOO MANY MOUTHS (TMM) prevents signal interference between these EPF/EPFL-ERECTA mediated signalling to ensure proper stomatal patterning^15,16^.

Upon ligand binding, ERECTA recruits universal coreceptor SOMATIC EMBRYOGENESIS RECEPTOR KINASES (SERKs), including SERK1, SERK3/BRI1-ASSOCIATED RECEPTOR KINASE1 (BAK1) and SERK4 (ref. 20). Immediate intracellular signalling components of ERECTA are shared for both inflorescence growth and stomatal development: those include receptor-like cytoplasmic kinases BR-SIGNALING KINASE1/2 (BSK1/2) and a cascade of mitogen-activated protein kinases (MAPKs), YODA (YDA)-MKK4/5-MPK3/6 (refs. 10,21–24). SERKs/BAK and BSKs were originally identified as components of brassinosteroid-activated BRI1 receptor complex^25–27^. The flagellin-activated FLS2 also forms a complex with its coreceptor BAK1 (ref. 28) and the signal is then mediated by the MAPK cascades^29^.

After receptor activation, the strength of cellular signalling must be promptly downregulated to avoid excessive or untimely signal outputs. Thus, the mechanism of signal downregulation is an integral part of receptor signalling. Studies on FLS2 and BRI1 have highlighted the role of receptor ubiquitination (ubiquitylation) for signal attenuation; interestingly, both ligand-activated FLS2 and BRI1 are ubiquitinated by the two identical plant U-box ubiquitin E3 ligases, PUB12 and PUB13, albeit in a slightly different manner^30,31^. Whereas PUB12/13 target several additional receptor kinases, ERECTA is not ubiquitinated by PUB12/13 (ref. 31). The U-box domain, which was originally identified from Ub Fusion Degradation 2 (UFD2) in yeast, mediates interaction with the ubiquitin-conjugating enzyme^32–35^. The first reported PUB protein is ARM Repeat Containing1 (ARC1), which interacts with the kinase domain of Brassica *S-locus* receptor kinases^36^. The PUB proteins constitute a family of more than 60 members in *Arabidopsis*, some of which are involved in a variety of environmental responses^37^. However, with the exception of a handful of members, their in vivo targets and functions remain unknown. The well-studied PUB proteins function in immunity and/or the stress response^37^, and whether PUB proteins attenuate RLKs regulating development remains an open question. For example, although PUB12/13 target BRI1, neither *pub12*, *pub13* nor the *pub12 pub13* double mutant exhibits any growth phenotypes nor do they enhance the elongated rosette phenotype of BRI1 overexpressors^31^. Thus, it is unclear whether PUB12/13 play a major role in brassinosteroid-mediated growth and developmental processes. A genetic study placed the possible role of PUB4 in CLAVATA signalling in the meristem^38^. However, the identity of PUB4 targets remains unknown^38^.

Here, we report two paralogous PUB proteins, PUB30 and PUB31, as key attenuators of ERECTA signaling transduction pathways for both inflorescence/pedicel growth and stomatal development. The *pub30 pub31* double mutant plants exhibit characteristic inflorescence with extreme pedicel elongation and a reduction in stomatal development. The *erecta* mutation is epistatic to *pub30 pub31*, indicating that PUB30 and PUB31 are redundantly required to downregulate ERECTA activity. We demonstrate that perception of EPF2 and EPFL6 peptides by ERECTA leads to the phosphorylation of PUB30/31 by BAK1 and stronger associations of PUB30/31 with ERECTA and BAK1. In this system, BAK1 acts both as a coreceptor kinase and a scaffold that recruits PUB30/31 to directly ubiquitinate ERECTA, but not BAK1 itself, for eventual degradation and signal attenuation. Our work reveals the modes of action and functions of a pair of PUB proteins in two ERECTA-mediated developmental processes and further suggests a broader view of how plant receptor kinases are attenuated upon signal activation.

## Results

### *PUB30/31* negatively regulate ERECTA-mediated plant growth

**Fig. 1:**
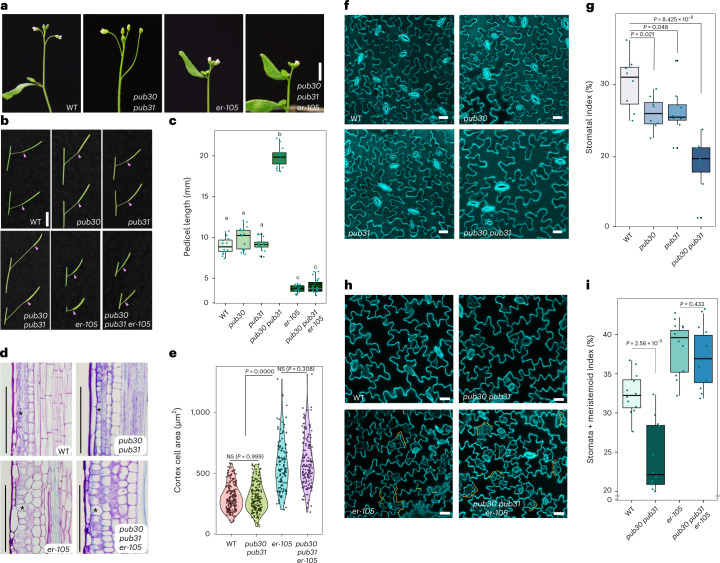
*PUB30/31* regulate inflorescence/pedicel growth and stomata development in the *ERECTA* pathway. **a**, Representative inflorescence of wild type (WT), *pub30 pub31*, *er-105* and *pub30 pub31 er-105* plants. **b**, Representative pedicels with fully expanded siliques of WT, *pub30*, *pub31*, *pub30 pub31*, *er-105* and *pub30 pub31 er-105* plants. Scale bar, 1 cm. **c**, Morphometric analysis of pedicel length from each genotype. Mature pedicels (*n* = 20) from 6-week-old plants were measured. One-way ANOVA followed by Tukey’s HSD test was performed and classified their phenotypes into categories (a, b and c). For *P* values see Extended Dataset 1. **d**, Longitudinal sections of mature pedicels from WT, *pub30 pub31*, *er-105* and *pub30 pub31 er-105* plants. Asterisks, representative cortex cells in each genotype. Scale bar, 200 μm. **e**, Quantitative analysis. Cortex cell area of representative mature pedicels from WT, *pub30 pub31*, *er-105* and *pub30 pub31 er-105* plants. Numbers of cells counted: *n* = 209 (WT), *n* = 165 (*pub30 pub31*), *n* = 129 (*er-105*) and *n* = 155 (*pub30 pub31 er-105*). Welch’s two-sample two-tailed unpaired *t*-test was performed for pairwise comparisons of WT versus *pub30 pub31*, *pub30 pub31* versus *er-105* and *er-105* versus *pub30 pub31 er-105*. *P* values are indicated in the graph. NS, not significant. **f**, Confocal microscopy of 10-day-old abaxial cotyledon epidermis of WT, *pub30*, *pub31* and *pub30 pub31*. Scale bar, 25 μm. **g**, Quantitative analysis. Stomatal index of the cotyledon abaxial epidermis from 10-day-old seedlings of respective genotypes (*n* = 8). Welch’s two-sample two-tailed unpaired *t*-test was performed for pairwise comparisons with the WT. *P* values are indicated in the graph. **h**, Confocal microscopy of 6-day-old abaxial cotyledon epidermis of WT, *pub30 pub31*, *er-105* and *pub30 pub31 er-105*. Scale bar, 25 μm. **i**, Quantitative analysis. Stomata + meristemoid index of the cotyledon abaxial epidermis from 6-day-old seedlings of respective genotypes (*n* = 12). Welch’s two-sample two-tailed unpaired *t*-test was performed for the pairwise comparisons of WT versus *pub30 pub31* as well as *er-105* versus *pub30 pub31 er-105*. *P* values are indicated in the graph. Source data

**Extended Data Fig. 1:**
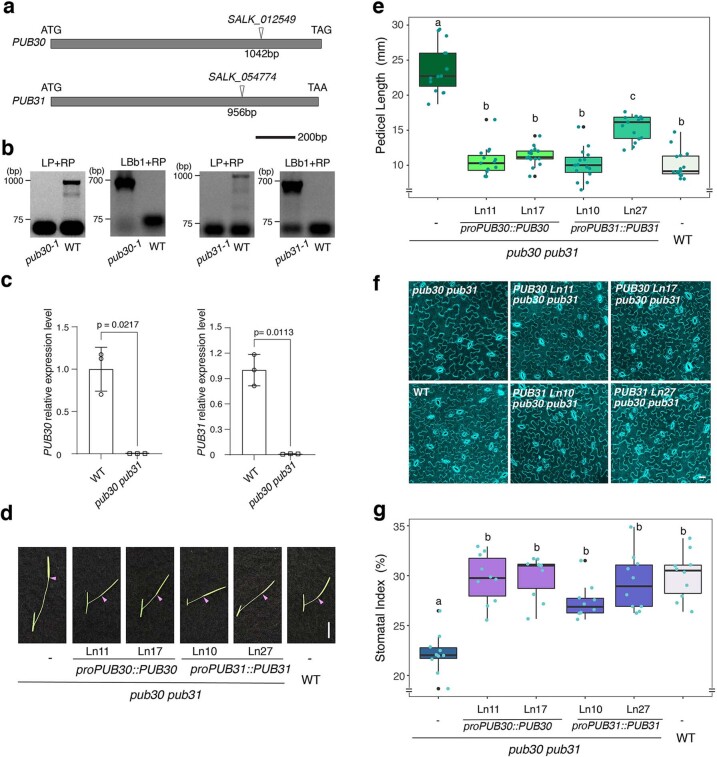
Analysis of *pub30* and *pub31* T-DNA mutants and genetic complementation of *pub30 pub31*. (**a**) Schematic diagrams of T-DNA insertion sites in *pub* mutants. The start codon (ATG) and the stop codon (TAG/ TAA) are indicated. Scale bar: 200 bp. (**b**) PCR identification of the T-DNA insertion in *pub30-1* and *pub31-1* with T-DNA specific primers (LBb1) and flanking primers (LP and RP). These alleles are referred as *pub30* and *pub31*, respectively. (**c**) RT-qPCR analysis of *PUB30* and *PUB31* in WT and *pub30 pub31* plants. Error bars represent SD (n = 3). Two-tailed paired Student’s t-test was performed. p values are in the graph. (**d**) Representative pedicels of mature siliques of *pub30 pub31*, *proPUB30::PUB30*; *pub30 pub31* Ln 11and 17, *proPUB31::PUB31*; *pub30 pub31* Ln 10and 27, and wild type plants. Scale bar: 1 cm. (**e**) Morphometric analysis of pedicel length from each genotype. 6-wk-old mature pedicels (n = 15) were measured. One-way ANOVA followed by Tukey’s HSD test was performed for comparing all other genotypes and classify their phenotypes into three categories (a, b, and c). For p values see Extended Dataset S1. (**f**) Confocal microscopy of 8-d-old abaxial cotyledon epidermis of *pub30 pub31*, *proPUB30::PUB30*; *pub30 pub31* Ln 11and 17, *proPUB31::PUB31*; *pub30 pub31* Ln 10 and 27, and wild type. Scale bar: 25 μm. (**g**) Quantitative analysis. Stomatal index (SI) of the cotyledon abaxial epidermis from 8-day-old seedlings of respective genotypes (n = 10). One-way ANOVA followed by Tukey’s HSD test was performed for comparing all other genotypes and classify their phenotypes into two categories (a and b). For p values see Supplementary Data.

Loss-of-function *erecta* mutant plants exhibit characteristic compact inflorescence and short pedicels (Fig. 1a–c)^7,8,14,39,40^. We hypothesized that potential negative regulators of ERECTA may confer the opposite phenotype—that is, extreme elongation of inflorescence and pedicels. With this in mind, we systematically surveyed the transfer DNA (T-DNA) insertion lines of *PUB* family genes. This led to the identification of *PUB30* and *PUB31* null mutant alleles (Methods and Extended Data Fig. 1a–c). Whereas single mutants of *pub30* and *pub31* do not show obvious growth phenotypes, the *pub30 pub31* double mutant produces elongated inflorescence with extremely long pedicels (Fig. 1a–c). Introduction of wild-type (WT) *PUB30* or *PUB31* coding sequences driven by their native promoters (*proPUB30::PUB30* and *proPUB31::PUB31*) into the *pub30 pub31* double mutant fully rescued the elongated pedicel phenotype (Extended Data Fig. 1d,e). These results indicate that *PUB30* and *PUB31* act redundantly to restrict the elongation of inflorescences and pedicels.

To address the genetic relationships of *PUB30/31* with *ERECTA*, we next generated the triple mutant of *pub30 pub31 er-105* (*erecta* null allele). The pedicel length of the triple mutant phenocopied that of *er-105* (Fig. 1a–c), indicating that the *erecta* mutation is epistatic to *pub30 pub31*. To further examine the underlying cellular basis of the *pub30 pub31* defects and its relationship with *erecta*, we analysed longitudinal sections of mature pedicels (Fig. 1d). It has been shown that short *erecta* pedicels accompany reduced cell proliferation and compensatory cell growth in the cortex layer^14,39^. In contrast to *erecta*, cortex cells in the *pub30 pub31* pedicels are small and highly organized (Fig. 1d, asterisks). Quantitative analysis detects no statistical difference in the cortex cell areas of the WT and *pub30 pub31* pedicels (Fig. 1e), indicating that the extremely elongated pedicel phenotype of *pub30 pub31* is due to excessive cell proliferation, but not cell expansion (Fig. 1d,e). The *pub30 pub31 er-105* pedicels exhibit large, expanded cortex cells that are statistically indistinguishable from *er-105* (Fig. 1d,e). Thus, the *erecta* mutation is epistatic to not only the overall pedicel length, but also the underlying cortex cell proliferation phenotype of *pub30 pub31*. Combined, our results suggest that *PUB30* and *PUB31* function as negative regulators of ERECTA-mediated inflorescence and pedicel growth.

### *PUB30/31* inhibit ERECTA pathway on stomatal development

It is well known that ERECTA family LRR-RKs enforce stomatal patterning^9^. Among the three members, ERECTA plays a major role in restricting the initiation of stomatal cell lineages^9,19^. To dissect the genetic relationship between *ERECTA* and *PUB30/31* in stomatal development, we first analysed the cotyledon epidermal phenotype (Fig. 1f,g). The *pub30* and *pub31* single mutants showed a slightly reduced stomatal index (number of stomata/(number of stomata + non-stomatal epidermal cells) × 100) compared with WT. The stomatal index was reduced notably in the *pub30 pub31* double mutant (Fig. 1f,g). Again, transgenic *pub30 pub31* plants expressing *proPUB30::PUB30* and *proPUB31:PUB31* fully rescued the stomatal phenotype of *pub30 pub31* (Extended Data Fig. 1f,g), indicating that *PUB30* and *PUB31* redundantly promote stomatal development.

We further characterized the stomatal phenotypes of *erecta*, *pub30 pub31* and *pub30 pub31 erecta*. Consistent with the epistatic effect of *erecta* over *pub30 pub31* with respect to inflorescence and pedicel growth (Fig. 1a–c), *erecta* is epistatic to *pub30 pub31* on stomatal development: *er-105* confers increased numbers of small stomatal lineage cells (Fig. 1h, orange brackets) thus vastly elevating the stomatal + meristemoid index (number of stomata + meristemoids)/(number of stomata + non-stomatal epidermal cells) × 100) (Fig. 1i) due to excessive asymmetric entry division events^9,19^. The *pub30 pub31 er-105* epidermis is phenotypically indistinguishable from the *er-105* epidermis (Fig. 1h,i). Thus, *PUB30/31* negatively regulate two distinct *ERECTA*-mediated developmental processes: inflorescence/pedicel elongation and stomatal lineage development.

### Ligand perception promotes interaction of ERECTA-PUB30/31

**Fig. 2:**
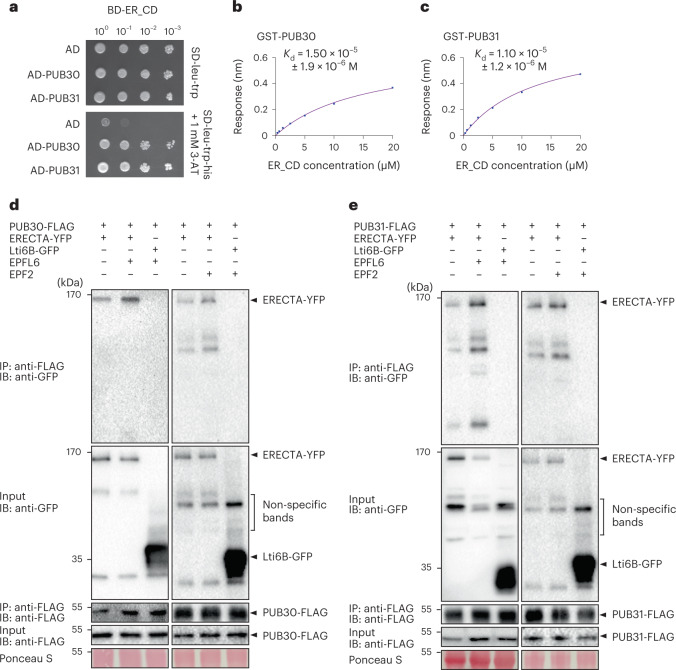
PUB30/31 directly interact with ERECTA. **a**, PUB30 and PUB31 interact with the cytoplasmic domain of ERECTA (ER_CD) in yeast. ER_CD was used as a bait. AD alone, PUB30 and PUB31 were used as prey. Yeast were spotted in tenfold serial dilutions on appropriate selection media. The experiment was repeated independently three times with similar results. **b**, Quantitative analysis of interactions between PUB30 and ER_CD using BLI. In vitro binding response curves for recombinantly purified GST-PUB30 and MBP-ER_CD at seven different concentrations (312.5, 625, 1,250, 2,500, 5,000, 10,000 and 20,000 nM) are shown. *K*_d_ values are indicated. Data are representative of two independent experiments. **c**, Quantitative analysis of interactions between PUB31 and ER_CD using BLI. In vitro binding response curves for recombinantly purified GST-PUB31 and MBP-ER_CD at seven different concentrations (312.5, 625, 1,250, 2,500, 5,000, 10,000 and 20,000 nM) are shown. *K*_d_ values are indicated. Data are representative of two independent experiments. **d**, Both EPFL6 and EPF2 induce the association of PUB30 with ERECTA in vivo. After treatment with the ligands of ERECTA, EPFL6 and EPF2, proteins from *proPUB30::PUB30-FLAG*; *proERECTA::ERECTA-YFP* and *proPUB30::PUB30-FLAG*; *Lti6B-GFP* plants were immunoprecipitated with anti-FLAG beads (IP) and the immunoblots (IB) were probed with anti-GFP and anti-FLAG antibodies, respectively. ERECTA-YFP was detected in the immunoprecipitated PUB30-FLAG complex. The experiment was repeated independently two times with similar results. **e**, Both EPFL6 and EPF2 induce the association of PUB31 with ERECTA in vivo. After treatment with the ligands of ERECTA, EPFL6 and EPF2, proteins from *proPUB31::PUB31-FLAG*; *proERECTA:: ERECTA-YFP* and *proPUB31::PUB31-FLAG*; *Lti6B-GFP* plants were immunoprecipitated with anti-FLAG beads (IP) and the immunoblots (IB) were probed with anti-GFP and anti-FLAG antibodies, respectively. ERECTA-YFP was detected in the immunoprecipitated PUB31-FLAG complex. The experiment was repeated independently two times with similar results.

**Extended Data Fig. 2:**
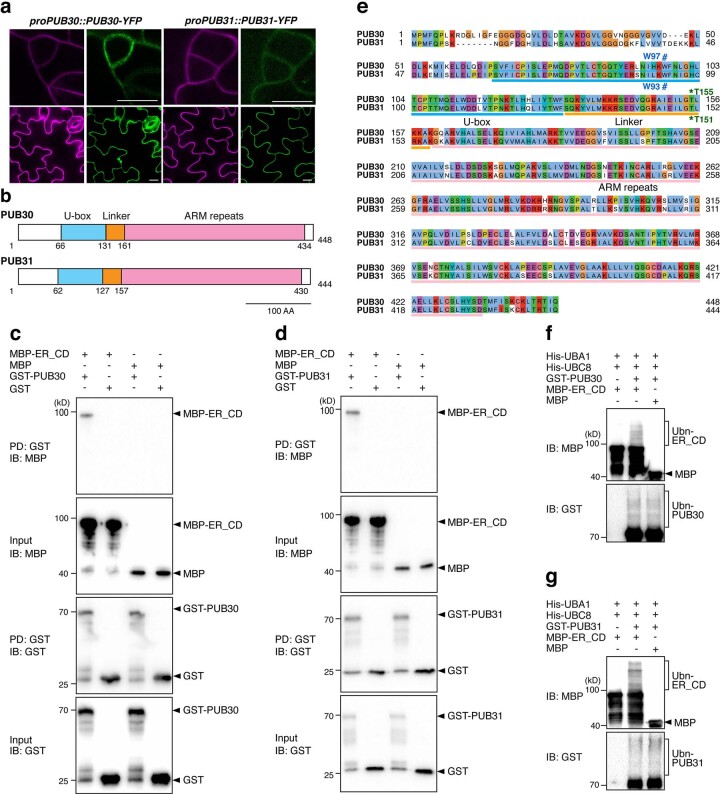
Subcellular localization, domain structures, interaction and ubiquitination assays of PUB30/31. (**a**) PUB30/31-YFP signals could be detected at the PM in both meristemoids and pavement cells. Confocal microscopy of 6-d-old abaxial cotyledon epidermis of *proPUB30::PUB30-YFP;* *pub30 pub31* or *proPUB31::PUB31-YFP;* *pub30 pub31* transgenic lines. Scale bar, 10 µm. (**b**) Schematic diagram of the domains of PUB30 and PUB31. PUB30 and PUB31 contain a U-box domain, a linker domain, and an ARMADILLO (ARM) repeat domain. The light blue, orange and pink color rectangles indicate the U-box domain, the linker domain, and the ARM repeats domain, respectively. Scale bar: 100 aa. (**c**) PUB30 interacts with ER_CD *in vitro*. MBP-ER_CD was pulled down (PD) by GST-PUB30 immobilized on Glutathione Sepharose 4B and analyzed by immunoblotting (IB) using an anti-MBP antibody. GST and MBP were used as negative controls. (**d**) PUB31 interacts with ER_CD *in vitro*. (**e**) The alignment of PUB30 and PUB31. Protein sequences above light blue lines, orange lines, and pink lines represent U-box domain, linker domain, and ARM repeats domain, respectively. The amino acid position of PUB30 is labeled on the top. The Tryptophan 97 (W97) site in PUB30 and the conserved W93 site in PUB31 are labeled with #. The Threonine 155 (T155) site in PUB30 and the conserved T151 site in PUB31 are labeled with *. (**f**) PUB30 ubiquitinates ERECTA *in vitro*. The ubiquitination of MBP-ER_CD was carried out by using GST-fused PUB30 as the E3 ligase, His-fused AtUBA1 as E1 activating enzyme, and His-fused UBC8 as E2 conjugating enzyme. MBP was used as a control. (**g**) PUB31 ubiquitinates ERECTA *in vitro*.

*ERECTA* functions in the same genetic pathway with *PUB30/31* to regulate pedicel growth and stomatal lineage development (Fig. 1). Just like previously reported localization patterns of ERECTA-YFP^41^, the Yellow Fluorescent Protein (YFP)-fused PUB30 and PUB31 expressed by their own native promoters (*proPUB30::PUB30-YFP* and *proPUB31::PUB31-YFP*) are detected in the developing cotyledon epidermis (Extended Data Fig. 2a). The similar expression and localization patterns of ERECTA and PUB30/31 imply their potential interaction. To test whether ERECTA interacts directly with PUB30/31, we first performed a yeast two-hybrid assay (Y2H). A truncated ERECTA protein with a cytosolic domain (ERECTA_CD), which contains the juxtamembrane domain, kinase domain and the C-terminal tail, was fused to the DNA-binding domain and used as bait. PUB30/31 are predicted cytoplasmic proteins, which contain a U-box domain, an ARM repeat and a linker domain in between (Extended Data Fig. 2b). Full-length PUB30 and PUB31 proteins were fused to the activation domain (AD). As shown in Fig. 2a, ERECTA_CD interacts with PUB30/31. Next, we confirmed the direct interaction of ERECTA_CD and PUB30/31 by in vitro pull-down assays using purified recombinant ERECTA_CD and full-length PUB30 and PUB31 proteins (Extended Data Fig. 2c,d). Finally, to quantitatively characterize the kinetics of protein–protein interactions between PUB30/31 and ERECTA_CD, we performed biolayer interferometry (BLI) assays (Methods). PUB30 and PUB31 bind with ERECTA_CD at a micromolar affinity (Fig. 2b,c). The observed weak affinity might be attributed to the transient and dynamic interactions of ERECTA and PUB30/31.

To examine the in vivo association of ERECTA with PUB30 and PUB31 in *Arabidopsis*, we further performed co-immunoprecipitation (co-IP) analyses using transgenic plants carrying epitope-tagged ERECTA (*proERECTA::ERECTA-YFP*) and PUB30/31 (*proPUB30::PUB30-FLAG* and *proPUB31::PUB31-FLAG*). ERECTA-YFP was detectable in the immunoprecipitated PUB30-FLAG or PUB31-FLAG complexes (Fig. 2d,e). It has been shown that EPF/EPFL ligand perception by ERECTA triggers the formation of an active receptor complex^20,42,43^. To test the hypothesis that receptor activation promotes the interaction of ERECTA and PUB30/31, we next treated the seedlings with EPFL6 and EPF2 peptides. Indeed, ERECTA strongly associates with PUB30 and PUB31 upon peptide stimulation (Fig. 2d,e). Combined, our results demonstrate that ERECTA physically interacts with PUB30 and PUB31 and their in vivo interactions are stimulated by corresponding peptide ligands regulating inflorescence elongation and stomatal development.

### PUB30 and PUB31 ubiquitinate ligand-activated ERECTA

As members of the PUB protein family, PUB30 and PUB31 possess sequence features of E3 ligases (Extended Data Fig. 2e). To determine whether PUB30/31 have E3 ubiquitin ligase activity and whether ERECTA is their substrate, we first performed in vitro ubiquitination assays. Faint laddering bands of ERECTA (MBP-ERECTA_CD) were detected when co-incubated with PUB30/31 proteins (glutathione S-transferase (GST) tagged-PUB30 and GST-PUB31), E2 ubiquitin-conjugating enzyme (His-UBC8) and E1 ubiquitin-activating enzyme (His-UBA1) (Extended Data Fig. 2f,g), indicating that PUB30/31 can ubiquitinate ERECTA in vitro.

**Fig. 3:**
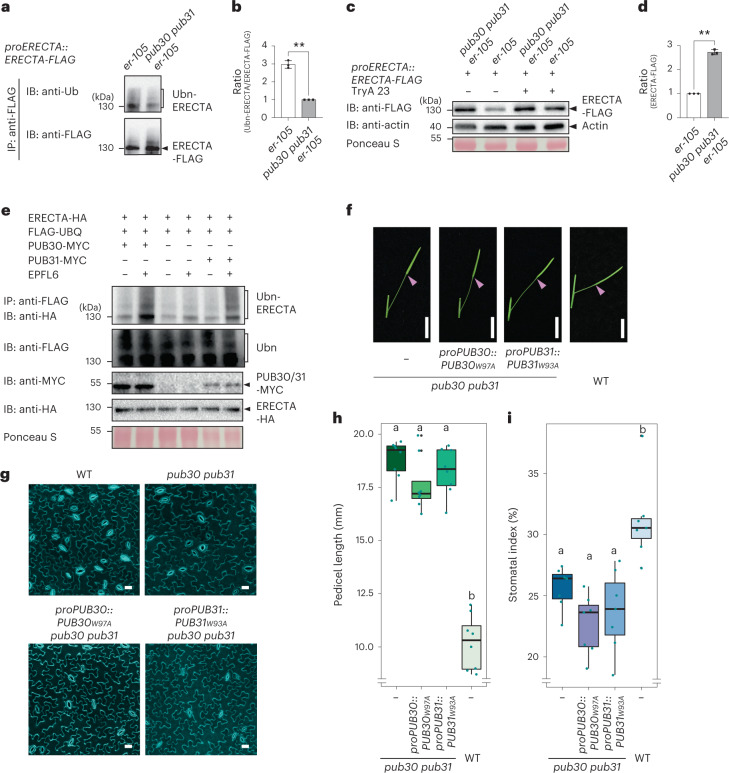
PUB30/31 ubiquitinate and regulate the protein abundance of ERECTA. **a**, Reduced in vivo ERECTA ubiquitination in *pub30 pub31* mutant. Immunoprecipitation (IP) was performed using anti-FLAG antibody on solubilized microsomal fraction protein extracts from ERECTA-FLAG plants in ‘WT’ or *pub30 pub31* background. Immunoblots (IB) were probed with anti-ubiquitin and anti-FLAG antibody, respectively. **b**, Quantitative analysis of ERECTA ubiquitination profiles. Error bars represent s.d. (*n* = 3). Asterisks indicate statistical significance using two-tailed paired Student’s *t*-test (*P* = 0.0039). **c**, ERECTA protein accumulation in WT and *pub30 pub31*, in the absence and presence of the endocytosis inhibitor Tyr A23. Data normalized by anti-actin. **d**, Quantification of ERECTA abundance (ERECTA/actin). Error bars represent s.d. (*n* = 3). Asterisks indicate statistical significance using two-tailed paired Student’s *t*-test (*P* = 0.0012). **e**, PUB30 or PUB31 mediates ERECTA ubiquitination in vivo. *Arabidopsis* protoplasts were cotransfected with ERECTA-HA, FLAG-UBQ and a control vector or PUB30-MYC or PUB31-MYC. Five micromolar EPFL6 was used for treatment for 1 h. After immunoprecipitation using anti-FLAG beads, the ubiquitinated ERECTA (Ubn-ERECTA) was probed with anti-HA antibody. The total ubiquitinated proteins were probed by anti-FLAG antibody and PUB30 or PUB31 proteins were probed by anti-MYC antibody. The inputs of ERECTA were probed with anti-HA antibody. **f**, Representative pedicels of mature siliques of *pub30 pub31*, *proPUB30::PUB30*_*W97A*_; *pub30 pub31*, *proPUB31::PUB31*_*W93A*_; *pub30 pub31* and WT plants. Scale bar, 1 cm. **g**, Confocal microscopy of 8-day-old abaxial cotyledon epidermis of *pub30 pub31*, *proPUB30::PUB30*_*W97A*_; *pub30 pub31*, *proPUB31::PUB31*_*W93A*_; *pub30 pub31* and WT plants. Scale bar, 25 μm. **h**, Morphometric analysis of pedicel length from each genotype. Mature pedicels (*n* = 15) from 6-week-old plants were measured. One-way ANOVA followed by Tukey’s HSD test was performed and classified their phenotypes into categories (a and b). For *P* values see Extended Dataset 1. **i**, Quantitative analysis. Stomatal index of the cotyledon abaxial epidermis from 8-day-old seedlings of respective genotypes (*n* = 7). One-way ANOVA followed by Tukey’s HSD test was performed and classified their phenotypes into categories (a and b). For *P* values see Supplementary Data.

**Extended Data Fig. 3:**
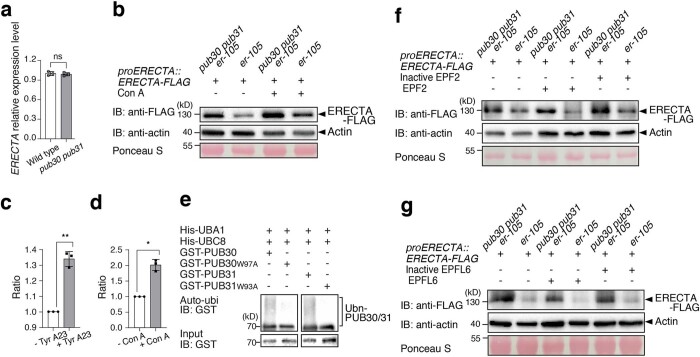
Regulation of ERECTA protein abundance and specific pathways involved. (**a**) RT-qPCR analysis of *ERECTA* in wild type and *pub30 pub31* plants. Error bars represent SD (n = 3). Two tailed paired Student’s t-test was performed (p = 0.7073). ns, not significant. (**b**) ERECTA protein accumulation in wildtype, and *pub30 pub31*, in the absence and presence of the vacuolar ATPase inhibitor Concanamycin A (Con A). Total proteins were isolated from 7-d-old seedlings and probed by anti-FLAG antibody. The protein inputs were equilibrated using anti-actin antibody. (**c**) Quantification of ERECTA abundance (ERECTA/Actin) in the absence and presence of Tyr A23. Error bars represent SD (n = 3). The asterisks indicate statistical significance by using two-tailed paired Student’s t-test (p = 0.0067). (**d**) Quantification of ERECTA abundance (ERECTA/Actin) in the absence and presence of Con A. Error bars represent SD (n = 3). The asterisks indicate statistical significance by using two-tailed paired Student’s t-test (p = 0.0102). (**e**) The residues (W97 in PUB30 and W93 in PUB31) are essential for their autoubiquitination, respectively. The *in vitro* autoubiquitination assays were performed using GST-PUB30/31 wild type or mutants as the E3 ligases. (**f**) ERECTA protein accumulation in wild type, and *pub30 pub31*, in the absence and presence of the EPF2 and inactive EPF2. Total proteins were isolated from 7-d-old seedlings and probed by anti-FLAG antibody. The protein inputs were equilibrated using anti-actin antibody. (**g**) ERECTA protein accumulation in wild type, and *pub30 pub31*, in the absence and presence of the EPFL6 and inactive EPFL6. Total proteins were isolated from 7-d-old seedlings and probed by anti-FLAG antibody. The protein inputs were equilibrated using anti-actin antibody.

To address the in vivo role of PUB30/31 in regulating ERECTA, we next compared the in vivo ubiquitination status of ERECTA in *erecta* null mutant, *er-105*, complemented with *proERECTA::ERECTA-FLAG* (hereafter referred to as ‘WT’) and *erecta pub30 pub31* triple mutant complemented with *proERECTA::ERECTA-FLAG* (‘*pub30 pub31*’) seedlings^44^ (Fig. 3a,b). The relative signal intensity ratio between ubiquitinated ERECTA detected by anti-ubiquitin antibody and immunoprecipitated ERECTA detected by anti-FLAG antibody, indicates that, in the absence *of PUB30/31*, ERECTA is less ubiquitinated in vivo (Fig. 3a,b). Poly-ubiquitinated proteins can be destined for degradation via the ubiquitin/26S proteosome pathway or the endocytic/vacuolar degradation pathway^45–49^. We subsequently examined whether PUB30/31 regulate ERECTA stability in vivo. Higher accumulation of ERECTA proteins (ERECTA-FLAG) was detected in *pub30 pub31* mutant background compared with the WT (Fig. 3c,d). By contrast, *ERECTA* transcript levels were not significantly different between WT and *pub30 pub31* seedlings (Extended Data Fig. 3a), indicating that the effects of *PUB30/31* on ERECTA accumulation is likely posttranslational. To test whether endocytic/vacuolar degradation pathways are involved in the PUB30/31-mediated ERECTA degradation, we subsequently treated the seedlings with Tyrphostin A23 (Tyr A23, an inhibitor of clathrin-mediated endocytosis)^50^ and Concanamycin A (Con A, a vacuolar ATPase inhibitor)^51^. As shown in Fig. 3c and Extended Data Fig. 3b–d, treatments of both Tyr A23 and Con A resulted in the significant increase in ERECTA protein accumulation in the ‘WT’ seedlings, suggesting that endocytosis followed by degradation in vacuole may be involved in the ERECTA degradation.

We further performed an in vivo ubiquitination assay using *Arabidopsis* protoplasts co-expressing epitope-tagged ERECTA (ERECTA-HA), PUB30 or PUB31 (PUB30-MYC or PUB31-MYC) and ubiquitin (FLAG-UBQ) (Methods). Laddering bands with high molecular mass proteins are detected after immunoprecipitation, indicative of the ubiquitination of ERECTA in vivo (Fig. 3e). Strikingly, application of EPFL6 peptide intensified the polyubiquitination of ERECTA by PUB30/31 (Fig. 3e), suggesting that PUB30/31 mediate the ligand-stimulated ERECTA ubiquitination.

Next, to address whether the ubiquitination activity of PUB30/31 is essential for their function as regulators of ERECTA-mediated processes, we introduced amino acid substitutions into PUB30/31 sequences that replace the conserved E2-binding tryptophan residue to alanine (PUB30_W97A_ or PUB31_W93A_) within their U-box motif (Extended Data Fig. 2e). In vitro auto-ubiquitination assays showed that these mutations (W97A in PUB30 or W93A in PUB31) diminished the ubiquitination activity of PUB30 and PUB31 (Extended Data Fig. 3e). Subsequently, these E2-binding defective PUBs were expressed by their native promoters in the *pub30 pub31* mutant. Neither transgenic *proPUB30::PUB30*_*W97A*_ nor *proPUB31::PUB31*_*W93A*_ was able to rescue the pedicel growth phenotype or stomatal phenotype (stomatal index) of *pub30 pub31* (Fig. 3f–i). Therefore, the E3 ligase activity of PUB30/31 is indeed required for proper pedicel elongation and stomatal development.

Finally, to address whether PUB30/31 target ligand-activated ERECTA, we examined whether EPF2/EPFL6 peptide perception induces the degradation of ERECTA. To this end, we performed peptide treatment experiments using bioactive as well as inactive (heat denatured) EPF2/EPFL6 peptides. As shown in Extended Data Fig. 3f,g, treatment of bioactive EPF2 or EPFL6, but not their inactive peptides, significantly decreased the accumulation of ERECTA protein in the ‘WT’ seedlings. By contrast, treatment of either active or inactive EPF2/EPFL6 peptides conferred much less change for the ERECTA protein abundance in the *pub30 pub31* mutant backgrounds. Based on these findings, we conclude that PUB30 and PUB31 downregulate the accumulation of ligand-stimulated ERECTA proteins via ubiquitination.

### Coreceptor of ERECTA, BAK1, interacts with PUB30 and PUB31

**Fig. 4:**
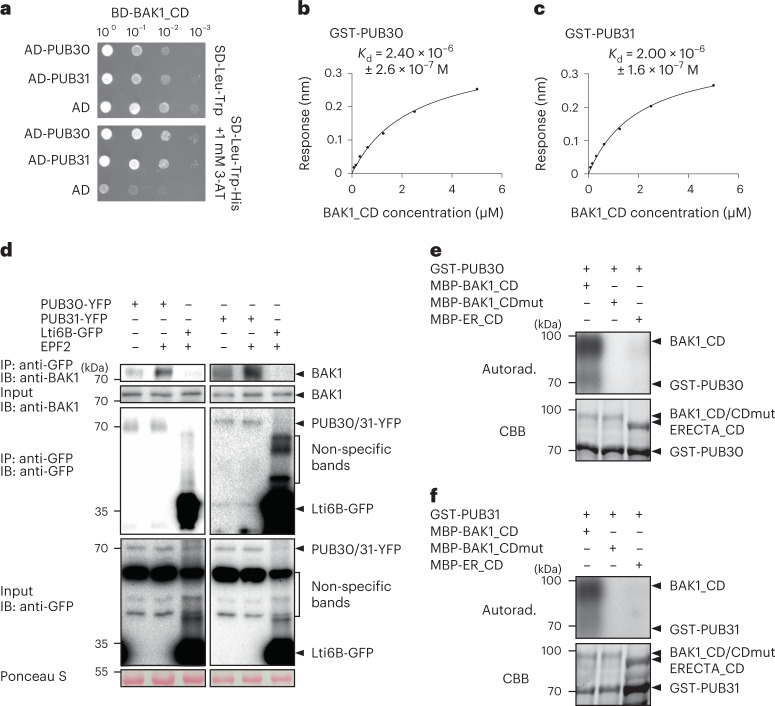
BAK1 interacts with and phosphorylates PUB30 and PUB31. **a**, PUB30 and PUB31 interact with BAK1_CD in yeast. BAK1_CD were used as bait. PUB30, PUB31 and AD alone were used as prey. Yeast clones were spotted in tenfold serial dilutions on appropriate selection media. The experiment was repeated independently three times with similar results. **b**, A quantitative analysis of interactions between PUB30 and BAK1_CD using BLI. In vitro binding response curves for recombinantly purified GST-PUB30 and MBP-BAK1_CD at seven different concentrations (78.125, 156.25, 312.5, 625, 1,250, 2,500 and 5,000 nM) are shown. *K*_d_ values are indicated on the right. Data are representative of two independent experiments. **c**, Quantitative analysis of interactions between PUB31 and BAK1_CD using BLI. Data are representative of two independent experiments. **d**, EPF2 induces the association of PUB30 and PUB31 with BAK1 in vivo. After treatment with EPF2, proteins from *proPUB30::PUB30-YFP*; *pub30 pub31*, *proPUB31::PUB31-YFP*; *pub30 pub31* and *Lti6B-GFP* plants were immunoprecipitated with anti-GFP beads (IP), and the immunoblots (IB) were probed with anti-BAK1 and anti-GFP antibodies, respectively. **e**, BAK1_CD phosphorylates PUB30 in vitro. The phosphorylation of GST-PUB30 was carried out by using MBP-BAK1_CD as the kinase. MBP-BAK1_CDmut was used as a negative control. MBP-ER_CD was also used as kinase for GST-PUB30. Autoradiography (Autorad.; upper) was used for phosphorylation detection, and Coomassie Brilliant Blue (CBB) staining (lower) was performed to show the protein loading. **f**, BAK1_CD phosphorylates PUB31 in vitro.

**Extended Data Fig. 4:**
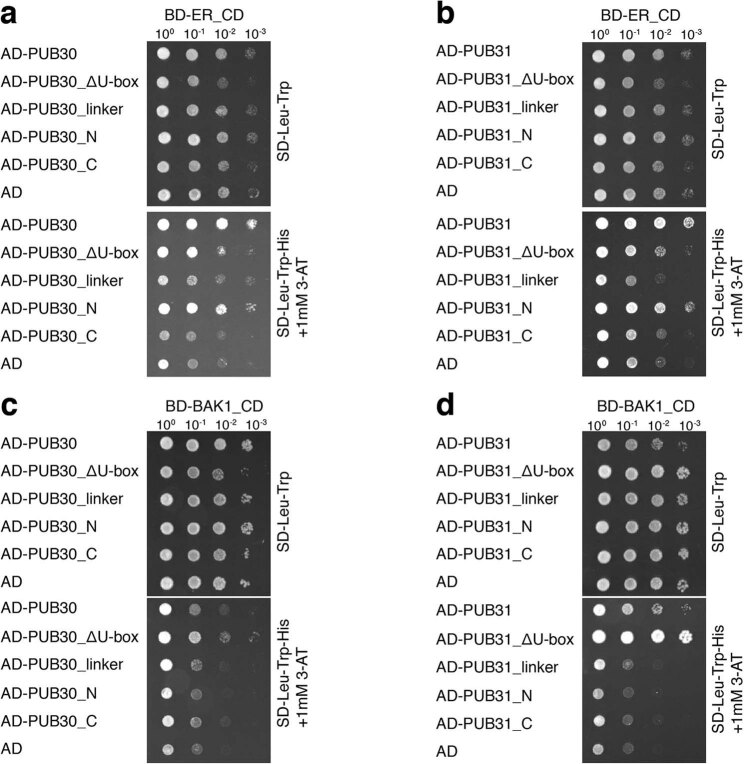
Domain interaction analysis of PUB30/31 with ERECTA and BAK1 in yeast. (**a**) The U-box domain of PUB30 is not essential for its interaction with ERECTA in yeast. Full length and different regions of PUB30 were used as baits. ER_CD and AD alone (as a negative control) were used as prey. Yeast clones were spotted in 10-fold serial dilutions on appropriate selection media. The experiment was repeated independently three times with similar results. For the constructs used here: PUB30 (1-65 + 132-448) is PUB30 (ΔU-box). PUB30 (132-200) contains the linker domain. PUB30 (1-200) is PUB30_N, which contains the U-box domain and linker domain. PUB30 (168-448) is PUB30_C, which does not contain U-box or linker domain. (**b**) U-box domain of PUB31 is not essential for its interaction with ERECTA in yeast. For the constructs used here: PUB31 (1-61 + 128-444) is PUB31 (ΔU-box). PUB31 (128-200) contains the linker domain. PUB31 (1-200) is PUB31_N, which contains the U-box domain and linker domain. PUB31 (168-444) is PUB31_C, which does not contain U-box or linker domain. (**c**) The U-box domain of PUB30 is not essential for its interaction with BAK1 in yeast. (**d**) The U-box domain of PUB31 is not essential for its interaction with BAK1 in yeast.

**Extended Data Fig. 5:**
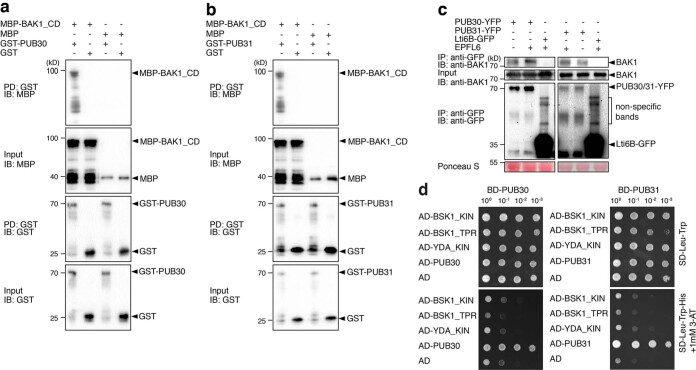
PUB30/31 interact with BAK1 but not with downstream BSK1 or YODA. (**a**) PUB30 interacts with BAK1_CD *in vitro*. MBP-BAK1_CD was pulled down (PD) by GST-PUB30 immobilized on Glutathione Sepharose 4B and analyzed by immunoblotting (IB) using an anti-MBP antibody. MBP and GST were used as negative controls. (**b**) PUB31 interacts with BAK1_CD *in vitro*. (**c**) EPFL6 induces the association of PUB30 and PUB31 with BAK1 *in vivo*. After treatment with EPFL6, proteins from *proPUB30::PUB30-YFP*; *pub30 pub31*, *proPUB31::PUB31-YFP*; *pub30 pub31* and *Lti6B-GFP* plants were immunoprecipitated with anti-GFP beads (IP), and the immunoblots (IB) were probed with anti-BAK1 and anti-GFP antibodies, respectively. The experiment was repeated independently two times with similar results. (**d**) PUB30 and PUB31 have no interaction with BSK1 or YODA (YDA) in yeast. PUB30 or PUB31 was used as bait. The TPR domain and kinase domain of BSK1, kinase domain of YDA, PUB30 or PUB31 (as a positive control), and AD alone (as a negative control) were used as prey. Yeast clones were spotted in 10-fold serial dilutions on appropriate selection media. The experiment was repeated independently three times with similar results.

EPF/EPFL ligands trigger the active receptor complex formation of ERECTA and its coreceptor BAK1 (ref. 20). We thus sought to decipher the regulatory relationships between BAK1 and PUB30/31. First, we asked whether BAK1 could directly interact with PUB30/31. As show in Fig. 4a, the cytosolic domain of BAK1 fused with DNA-binding domain (BD-BAK1_CD) interacts with PUB30/31 (AD-PUB30/31) in the Y2H assays. Further domain analysis in yeast implies that the domains of PUB30/31 encompassing the linker region mediates the association with BAK1 and ERECTA and that the U-box domains are dispensable for the interaction (Extended Data Fig. 4). The in vitro pull-down assays confirmed the interaction between recombinant PUB30/31 (GST-PUB30/31) with BAK1_CD (MBP-BAK1_CD) (Extended Data Fig. 5a,b). To quantitatively characterize the interaction property of BAK1_CD with PUB30/31, we further performed the BLI assays (Fig. 4b,c). Compared with ERECTA_CD (Fig. 2b, c), BAK1_CD exhibited stronger physical interaction in the order of magnitude with PUB30 and PUB31 (Fig. 4b,c). We next performed co-IP analyses to investigate the in vivo association of BAK1 with PUB30 and PUB31 in *Arabidopsis*. Just like the in vivo interaction of ERECTA with PUB30/31 (Fig. 2d,e), BAK1 was weakly detected in the absence of the peptide treatment. Upon EPF2 or EPFL6 peptide incubation, however, BAK1 strongly associated with PUB30 and PUB31 (Fig. 4d and Extended Data Fig. 5c).

It has been reported that EPF/EPFL signal perceived by ERECTA-BAK1/SERKs is subsequently transduced via BSK1/2 and YODA MAPK cascade^10,52^. To address the extent to which PUB30/31 associate with the ERECTA signalling components, we expanded our protein–protein interaction assays. As shown in Extended Data Fig. 5d, no interaction of PUB30/31 with BSK1/2 as well as YODA was detected by Y2H. Combined, our results demonstrate that the coreceptor of ERECTA, BAK1, also interacts with PUB30/31 in the EPF/EPFL ligand-stimulated manner and suggest that the regulation of PUB30/31 activity likely occurs at the level of the active receptor complex but not further downstream components.

### BAK1 phosphorylates PUB30 and PUB31

**Extended Data Fig. 6:**
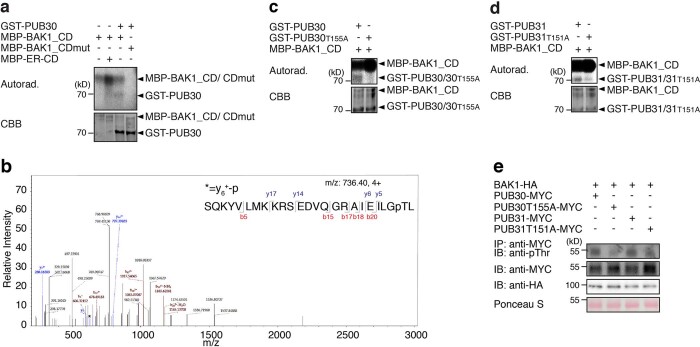
BAK1 phosphorylates PUB30 and PUB31 in the linker domain. (**a**) BAK1_CD phosphorylates PUB30 *in vitro*. The phosphorylation of GST-PUB30 was carried out by using MBP-BAK1_CD as the kinase. MBP-BAK1_CD alone was used as a negative control. Combination of MBP-BAK1_CDmut and GST-PUB30 was also used as a negative control. In contrast, combination of MBP-BAK1_CD and MBP-ER_CD, but not with GST-PUB30 was used as a positive control. Autoradiography (Top) was occupied for phosphorylation detection, and CBB staining (Bottom) was performed to show the protein loading. (**b**) MS/MS spectra for selected in vitro phosphorylation site of PUB30: Thr155. (**c**) PUB30 T155 is required for BAK1-mediated phosphorylation. The threonine residue (PUB30_T155_) was mutated to alanine (A). GST-tagged wild-type or mutated PUB30 proteins were subjected to an *in vitro* kinase assay using MBP-BAK1_CD as the kinase. (**d**) PUB31 T151 is required for BAK1-mediated phosphorylation. The threonine residue (PUB31_T151_) was mutated to alanine (A). GST-tagged wild-type or mutated PUB31 proteins were subjected to an *in vitro* kinase assay using MBP-BAK1_CD as the kinase. (**e**) BAK1 mediates PUB30/31 phosphorylation *in vivo*. Arabidopsis protoplasts were co-transfected with HA-tagged BAK1 (BAK1-HA), and MYC-tagged wild type and phosphonull versions of PUB30 or PUB31 and incubated for 8 h followed by treatment with 5 μM EPFL6 for 1 h in the presence of 2 μM MG132. After immunoprecipitation using anti-MYC beads, the phosphorylated PUB30/31 (wild-type and phosphonull versions) was probed with anti-pThr antibody. The input PUB30 or PUB31 proteins and BAK1 were probed with anti-MYC antibody and anti-HA antibody, respectively.

Both ERECTA and BAK1 are functional protein kinases^27,53^ and interact with PUB30/31 upon ligand treatment (Figs. 2 and 4). Does ERECTA or BAK1 phosphorylate PUB30/31? To address this question, we first performed in vitro kinase assays using purified recombinant epitope-tagged proteins and radioactive ATP (Fig. 4e,f and Extended Data Fig. 6a). BAK1_CD (MBP-BAK1_CD) strongly autophosphorylated itself and trans-phosphorylated PUB30/31 (GST-PUB30/31) (Fig. 4e,f). On the other hand, BAK1_CDmut, in which kinase activity is abolished by the substitution of an invariable lysine to methionine (K364M), showed no autophosphorylation or phosphorylation of GST-PUB30/31 (Fig. 4e,f and Extended Data Fig. 6a). These results suggest that BAK1 phosphorylates PUB30 and PUB31 in vitro. We also tested whether ERECTA phosphorylates PUB30/31. However, as reported previously^20^, ERECTA_CD exhibited weak/negligible kinase activity. Consequently, we detected no phosphorylation of PUB30 or PUB31 by ERECTA_CD (Fig. 4e,f).

To further identify the exact residue(s) of PUB30/31 phosphorylated by BAK1, we performed liquid chromatography tandem mass spectrometry analysis after an in vitro phosphorylation reaction using MBP-BAK1_CD as kinase and GST-PUB30 as substrate. The threonine 155 (T155) residue, located in the linker domain of PUB30, which serves as interaction domain with BAK1 and ERECTA, was identified as a phosphosite (Extended Data Figs. 4 and 6b, and Supplementary Table 1). The T155 in PUB30 is conserved in PUB31 as threonine 151 (T151) (Extended Data Fig. 2e). These threonine residues were replaced by alanines (PUB30_T155A_ or PUB31_T151A_) to confirm that they are the major phosphosites. Indeed, GST-PUB30_T155A_ and GST-PUB31_T151A_ were less phosphorylated by BAK1_CD in vitro (Extended Data Fig. 6c,d). Furthermore, we performed an in vivo phosphorylation assay to confirm the importance of these phosphorylation sites. After incubation together with BAK1 and treatment with EPFL6, the phosphorylation level of WT PUB30/31 was much higher than that of phosphonull versions of PUB30/31 (Extended Data Fig. 6e). These results further support that single amino acid residues in the linker domain of PUB30/31 are the major in vitro phosphosites by BAK1.

### BAK1 is required for ERECTA-PUB30/31 interaction

**Extended Data Fig. 7:**
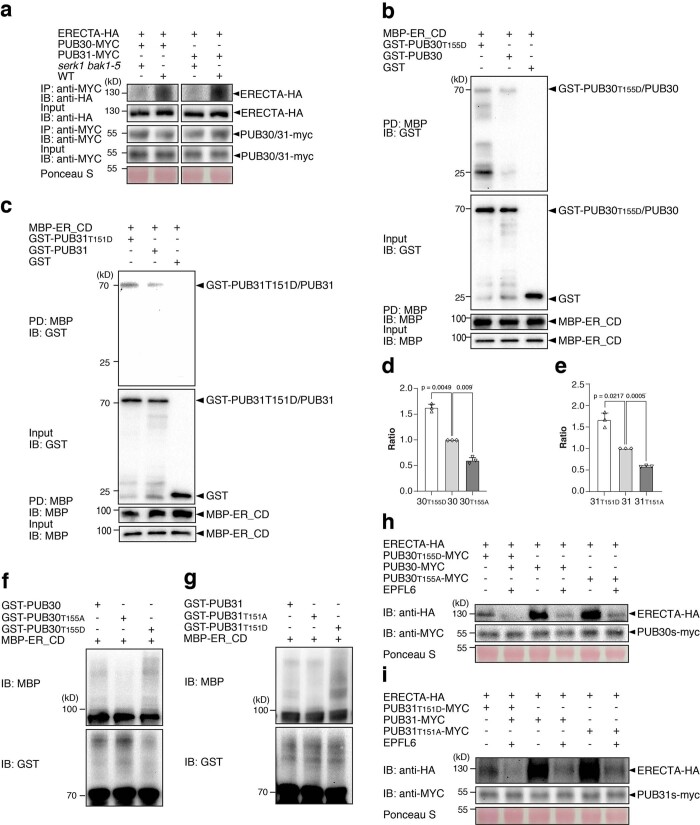
Phosphorylation of PUB30 and PUB31 by BAK1 is required for their association with and ubiquitination of ERECTA. (**a**) The association of PUB30/31 with ERECTA *in vivo* in wild type (WT) and *serk1bak1-5*. ERECTA-HA and PUB30/PUB31-MYC were co-transfected into protoplast. After incubation for 8 h, pretreatment with 2 μM MG132 for 1 h and treatment with 5 μM EPFL6 for 1 h, total proteins were immunoprecipitated with anti-MYC beads (IP), and the immunoblots (IB) were probed with anti-HA and anti-MYC antibodies, respectively. (**b**) The phosphor-mutations of PUB30 affect its interaction with ER_CD *in vitro*. GST-PUB30_T155D_ or GST-PUB30 was pulled down (PD) by MBP-ER_CD immobilized on Amylose Resin and analyzed by immunoblotting (IB) using an anti-GST antibody. GST was used as a negative control. (**c**) The phosphor-mutations of PUB31 affect its interaction with ER_CD *in vitro*. GST-PUB31_T151D_ or GST-PUB31 was pulled down (PD) by MBP-ER_CD and analyzed by immunoblotting (IB) using anti-GST antibody. GST was used as a negative control. (**d, e**) Quantification of *in vivo* interaction strength of ERECTA with wild-type or various phosphor-mutated PUB30/31. Error bars represent SD (n = 3). Two-tailed paired Student’s t-test was performed. p values are indicated in the graph. (**f**) T155 phosphorylation of PUB30 is not prerequisite for its E3 ligase activity, but important for the ubiquitination of ER_CD. *In vitro* ubiquitination assays were performed with the indicated recombinant proteins. PUB30 auto-ubiquitination and ER_CD ubiquitination were detected with immunoblot using anti-GST antibody and anti-MBP antibody, respectively. (**g**) T151 phosphorylation of PUB31 is not prerequisite for its E3 ligase activity, but important for the ubiquitination of ER_CD. (**h**) EPFL6 treatment destabilizes ERECTA-HA in Arabidopsis protoplasts co-expressing different versions of PUB30 (PUB30_T155D_-MYC, PUB30-MYC, PUB30_T155A_-MYC). Protoplasts expressing the indicated proteins were treated with 50 μM CHX and 5 μM EPFL6 for 3 hr before total protein was examined with immunoblot. The experiment was repeated independently two times with similar results. (**i**) EPFL6 treatment destabilizes ERECTA-HA in Arabidopsis protoplasts co-expressing different versions of PUB31 (PUB31_T151D_-MYC, PUB31-MYC, PUB31_T151A_-MYC). Experiments are done as described in (h). The experiment was repeated independently two times with similar results.

We have shown that EPF/EPFL ligand perception intensifies the association of ERECTA as well as BAK1 with PUB30/31, leading to subsequent ubiquitination and degradation of ERECTA by PUB30/31 (Figs. 2–4). These findings suggest that the ligand-activated ERECTA-BAK1 receptor complex recruits and activates PUB30/31. To address this question, we first examined whether BAK1 is required for the interactions of ERECTA and PUB30/31. For this purpose, we performed in vivo co-IP experiments using protoplasts from the *serk1 bak1-5* double mutant, which confers a stomatal development phenotype but not severe growth defects^20^. We observed a striking reduction in the association of ERECTA-HA with PUB30-MYC as well as PUB31-MYC in *serk1 bak1-5* background (Extended Data Fig. 7a). The results indicate that BAK1 (and SERK1) is required for ERECTA-PUB30/31 interaction.

**Fig. 5:**
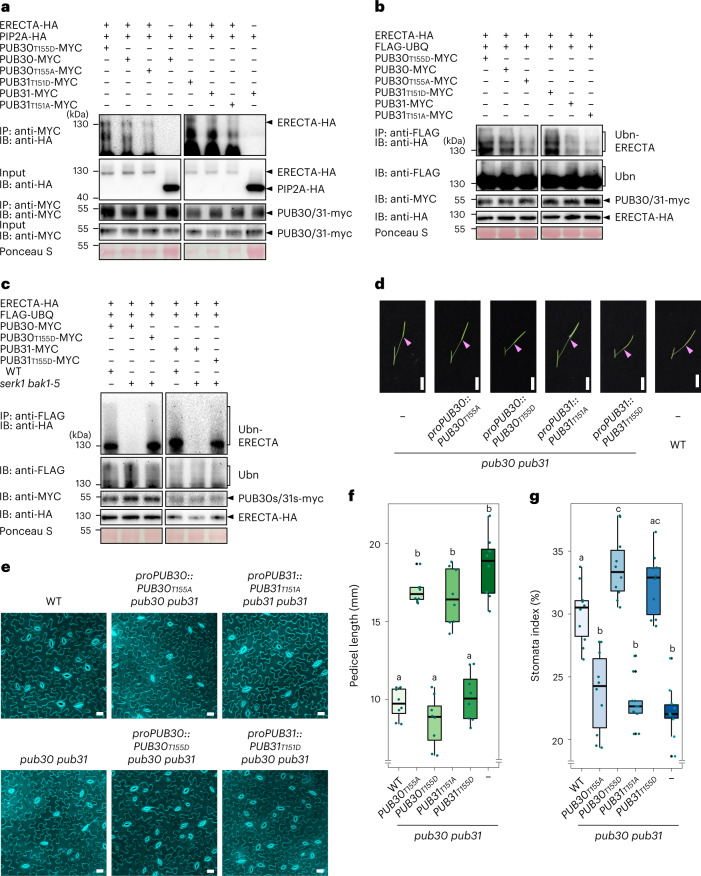
Phosphorylation of PUB30 and PUB31 by BAK1 is essential for the function of PUB30/31. **a**, Association of wild type (WT) or various phosphor-mutated PUB30/31 with ERECTA in vivo. ERECTA-HA and PUB30 or PUB31 (WT or various phosphor-mutated)-MYC plasmids were transfected into protoplast. Combinations of PIP2A-HA and PUB30 or PUB31-MYC plasmids were used as negative controls. Five micromolar EPFL6 was used for treatment for 1 h. Total proteins were immunoprecipitated with anti-MYC beads (IP), and the immunoblots (IB) were probed with anti-HA and anti-MYC antibodies, respectively. **b**, In vivo ERECTA ubiquitination by WT or various phosphor-mutated PUB30/31. *Arabidopsis* protoplasts were cotransfected with ERECTA-HA, FLAG-UBQ, and together with PUB30_T155D_-MYC, PUB30-MYC, PUB30_T155A_-MYC, PUB31_T151D_-MYC, PUB31-MYC, PUB31_T151A_-MYC. Five micromolar EPFL6 was used for treatment for 1 h. After immunoprecipitation using anti-FLAG beads, the ubiquitinated ERECTA was probed with anti-HA antibody. The total ubiquitinated proteins were probed by anti-FLAG antibody and PUB30 or PUB31 proteins were probed by anti-MYC antibody. **c**, In vivo ERECTA ubiquitination by PUB30/31 in WT and *serk1 bak1-5* double mutant using *Arabidopsis* protoplasts. The total ubiquitinated proteins were probed by anti-FLAG antibody and PUB30 or PUB31 proteins were probed by anti-MYC antibody. The input levels of ERECTA were probed with anti-HA antibody. **d**, Representative pedicels and mature siliques of *pub30 pub31*, *proPUB30::PUB30*_*T155A*_; *pub30 pub31*, *proPUB30::PUB30*_*T155D*_; *pub30 pub31*, *proPUB31::PUB31*_*T151A*_; *pub30 pub31*, *proPUB31::PUB31*_*T151D*_; *pub30 pub31* and WT plants. Scale bar, 1 cm. **e**, Confocal microscopy of 8-day-old abaxial cotyledon epidermis of *pub30 pub31*, *proPUB30::PUB30*_*T155A*_; *pub30 pub31*, *proPUB30::PUB30*_*T155D*_; *pub30 pub31*, *proPUB31::PUB31*_*T151A*_; *pub30 pub31*, *proPUB31::*PUB31_T151D_; *pub30 pub31* and WT plants. Scale bar, 25 μm. **f**, Morphometric analysis of pedicel length from each genotype. Six-week-old mature pedicels (*n* = 8) were measured. One-way ANOVA followed by Tukey’s HSD test was performed and classified their phenotypes into categories (a and b). For *P* values see Extended Dataset 1. **g**, Quantitative analysis. Stomatal index of the cotyledon abaxial epidermis from 8-day-old seedlings of respective genotypes (*n* = 10). One-way ANOVA followed by Tukey’s HSD test was performed and classified their phenotypes into categories (a, b and c). For *P* values see Extended Dataset 1.

Next, to address whether the phosphorylation of PUB30/31 at the T151/T155 residues by BAK1 promotes the association PUB30/31 with ERECTA, we performed a series of in vitro and in vivo assays. The in vitro pull-down experiments showed that the phosphomimetic versions of PUB30/31 exhibit stronger interaction with ERECTA_CD than their WT versions (Extended Data Fig. 7b,c). Subsequently, we performed the in vivo co-IP experiments using *Arabidopsis* protoplasts expressing the epitope-tagged ERECTA and WT, phosphomimetic and phosphonull versions of PUB30/31 (Methods). The association of ERECTA-HA with PUB30_T155A_-MYC as well as PUB31_T151A_-MYC was markedly reduced compared with the WT versions of PUB30/31-MYC (Fig. 5a and Extended Data Fig. 7d,e). By contrast, the phosphomimetic mutant PUB30_T155D_-MYC and PUB31_T151D_-MYC exhibited stronger interactions with ERECTA than the WT PUB30 and PUB31, respectively (Fig. 5a and Extended Data Fig. 7d,e). Combined, these results indicate that BAK1-mediated phosphorylation of PUB30/31 intensifies their association with ERECTA.

### ERECTA ubiquitination–degradation by PUB30/31 require BAK1

To investigate whether phosphorylation of PUB30/31 by BAK1 is required for the ubiquitination of ERECTA by PUB30/31, we first performed in vitro ubiquitination assays of MBP-ERECTA_CD by purified recombinant GST-PUB30/31 as well as the phosphomimetic (PUB30_T155D_ and PUB31_T151D_) and phosphonull (PUB30_T155A_ and PUB31_T151A_) versions. The ubiquitination of ERECTA_CD by PUB30_T155D_ and PUB31_T151D_ was stronger than that by WT PUB30 and PUB31, respectively (Extended Data Fig. 7f,g). By contrast, the ubiquitination of ERECTA_CD by PUB30_T155A_ and PUB31_T151A_ was slightly reduced compared with the WT versions of PUB30/31 (Extended Data Fig. 7f,g). No difference was observed for the auto-ubiquitination of PUB30/31 WT, phosphomimetic and phosphonull versions, as detected by anti-GST immunoblots (Extended Data Fig. 7f,g). Therefore, although we cannot fully exclude the possibility that these phosphomimetics/phosphonull mutations may affect the protein structure, our results indicate that the phosphorylation of PUB30/31 by BAK1 may not have a direct effect on their ubiquitination activity per se.

We next performed in vivo ubiquitination assays in *Arabidopsis* protoplasts upon EPFL6 peptide application, the condition that triggers polyubiquitination of ERECTA (Fig. 3e). As evidenced by the reduced ladder-like smear formation, the phosphonull mutants, PUB30_T155A_-MYC and PUB31_T151A_-MYC, conferred reduced ubiquitination of ERECTA-HA compared with WT PUB30-MYC and PUB31-MYC, respectively (Fig. 5b). By contrast, the phosphomimetic mutants, PUB30_T155D_-MYC and PUB31_T151D_-MYC caused increased ubiquitination on ERECTA-HA compared with WT versions of PUB30/31 (Fig. 5b). Combined, these results support that the phosphorylation of PUB30/31 linker domains promotes the ubiquitination of ERECTA.

To unambiguously address whether BAK1 is required for PUB30/31-mediated ubiquitination of ERECTA, we further performed the in vivo ubiquitination assays using *Arabidopsis* WT and *serk1 bak1-5* protoplasts. As evidenced by the reduced ladder-like smear formation, PUB30-MYC and PUB31-MYC conferred reduced ubiquitination of ERECTA-HA in the *serk1 bak1-5* background (Fig. 5c). By contrast, the phosphomimetic mutants, PUB30_T155D_-MYC and PUB31_T151D_-MYC caused comparable ubiquitination of ERECTA-HA regardless of the presence or absence of BAK1 (and SERK1) (Fig. 5c).

Finally, we tested the effects of PUB30/31 phosphorylation by BAK1 on degradation of ERECTA protein. For this purpose, we co-expressed ERECTA and WT, phosphomimetic and phosphonull versions of PUB30/31 in *Arabidopsis* protoplast and performed cotreatment with EPFL6 and cycloheximide (de novo protein synthesis inhibitor). Upon EPFL6 treatment, phosphomimetic mutants, PUB30_T155D_-MYC and PUB31_T151D_-MYC caused a distinct decrease in ERECTA-HA protein level (Extended Data Fig. 7h,i). By contrast, phosphonull mutants, PUB30_T155A_-MYC and PUB31_T151A_-MYC conferred less decrease in ERECTA-HA protein level (Extended Data Fig. 7h,i). Taken together, our results show that the BAK1 phosphorylation of PUB30 and PUB31 at T155 and T151 residues, respectively, facilitates the ubiquitination and subsequent degradation of ERECTA.

### BAK1 is not a substrate of PUB30/31

**Extended Data Fig. 8:**
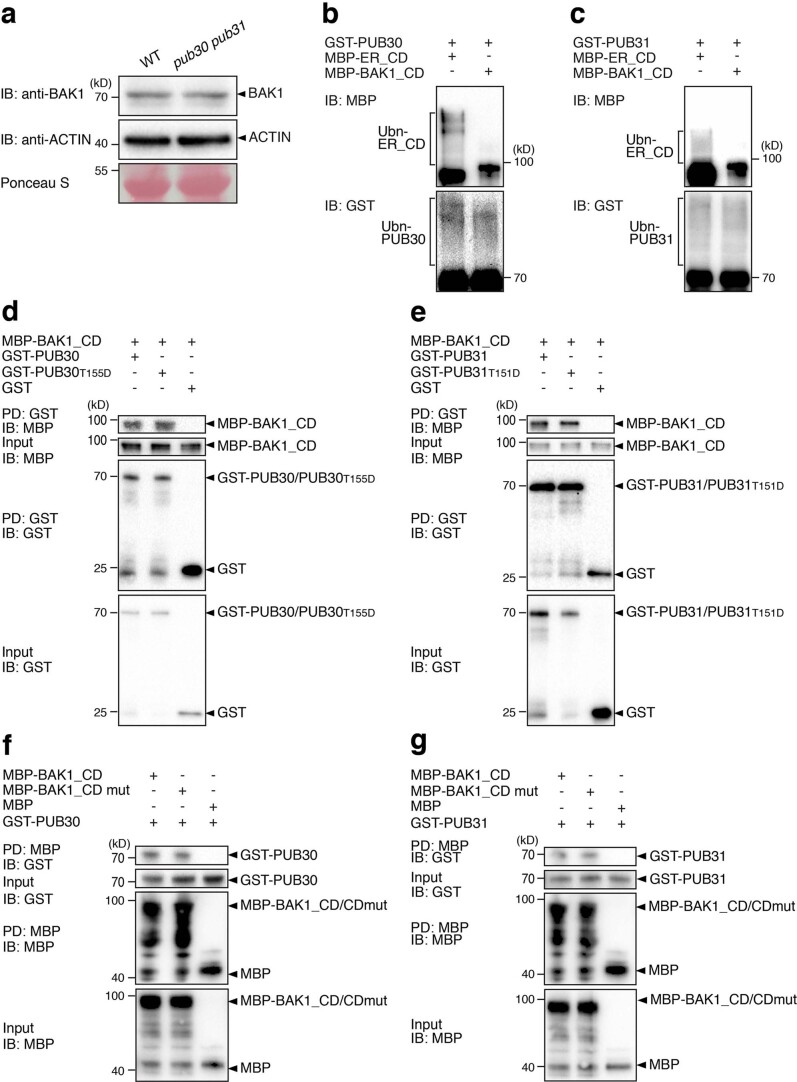
BAK1 is not a substrate of PUB30/31. (**a**) BAK1 protein accumulation in wild type (WT) and *pub30 pub31*. Total proteins were isolated from 7-d-old seedlings and probed by anti-BAK1 antibody. The protein inputs were equilibrated using anti-actin antibodies. (**b**) BAK1 is not ubiquitinated by PUB30 *in vitro*. The ubiquitination assay of MBP-BAK1_CD was carried out by using GST-fused PUB30 as the E3 ligase, His-fused AtUBA1 as E1 activating enzyme, and His-fused UBC8 as E2 conjugating enzyme. MBP-ER_CD was used as a positive control. (**c**) BAK1 is not ubiquitinated by PUB31 *in vitro*. (**d**) The phosphorylation of PUB30 by BAK1 does not affect its interaction with BAK1_CD *in vitro*. MBP-BAK1_CD was pulled down (PD) by GST-PUB30 or GST-PUB30_T155D_ immobilized on Glutathione Sepharose 4B and analyzed by immunoblotting (IB) using an anti-MBP antibody. GST was used as a negative control. (**e**) The phosphorylation of PUB31 by BAK1 does not affect its interaction with BAK1_CD *in vitro*. (**f**) The kinase activity of BAK1 does not affect its interaction with PUB30 *in vitro*. GST-PUB30 was pulled down (PD) by MBP-BAK1_CD or MBP-BAK1_CDmut immobilized on Amylose Resin and analyzed by immunoblotting (IB) using an anti-GST antibody. MBP was used as a negative control. (**g**) The kinase activity of BAK1 does not affect its interaction with PUB31 *in vitro*.

Our experimental evidence supports the model that BAK1 plays a key role in mediating the downregulation of ligand-activated ERECTA by PUB30/31 to attenuate signal transduction. The obvious and important question is whether BAK1 itself is also a substrate of PUB30/31. To address this question, we first examined whether PUB30/31 regulate the stability of BAK1 in vivo. Abundance of the endogenous BAK1 proteins, as detected by anti-BAK1 antibody, is similar in WT and *pub30 pub31* seedlings (Extended Data Fig. 8a), indicating that PUB30/31 do not have discernible effects on BAK1 protein levels. Next, we performed in vitro ubiquitination assays of BAK1. Compared with the positive control ERECTA (MBP-ER_CD), no laddering bands of BAK1 (MBP-BAK1_CD) were detected when co-incubated with PUB30/31 proteins (GST-PUB30 and GST-PUB31), His-UBC8 (E1) and His-UBA1 (E2) (Extended Data Fig. 8b,c), indicating that BAK1 is not ubiquitinated by PUB30/31 in vitro.

Because BAK1 directly phosphorylates PUB30/31 (Fig. 4 and Extended Data Fig. 6), we further tested whether phosphorylation status of PUB30/31 affects its interaction with BAK1 by in vitro pull-down experiments. As shown in Extended Data Fig. 8d,e, both the WT and phosphomimetic versions of PUB30/31 exhibit similar interaction with BAK1_CD. We further test whether the kinase activity of BAK1 is essential for its interaction with PUB30/31. Again, the in vitro pull-down experiments showed that BAK1_CD and BAK1_CDmut had similar interaction with PUB30/31 (Extended Data Fig. 8f,g). On the basis of these findings, we conclude that BAK1 is unlikely a substrate of PUB30/31 but rather an indispensable kinase and a scaffold to bring PUB30/31 to a proximity of ligand-activated ERECTA.

### PUB30/31 biological functions require phosphorylation by BAK1

**Extended Data Fig. 9:**
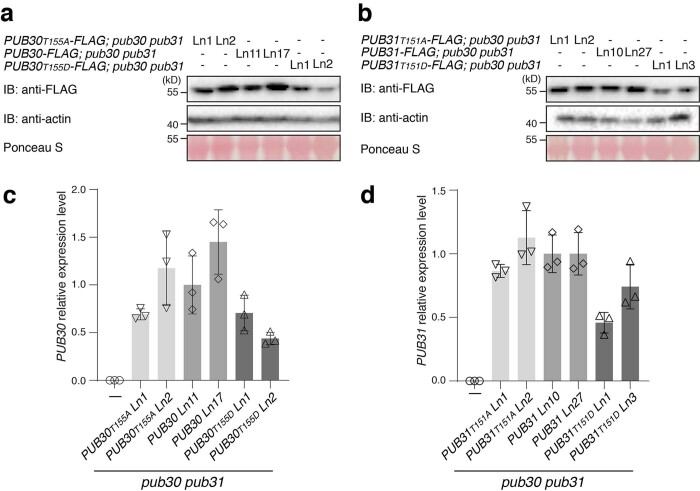
Protein and transcript levels of phosphonull and phosphomimetics PUB30/31 in Arabidopsis seedlings. (**a**) The protein levels of wild-type or various phosphosite mutated PUB30 in the Arabidopsis complementation lines. Total proteins were isolated from 7-d-old seedlings and probed by anti-FLAG antibody. Anti-actin antibody was used as a native control. (**b**) The protein levels of wild-type or various phosphosite mutated PUB31 in the Arabidopsis complementation lines. Total proteins were isolated from 7-d-old seedlings and probed by anti-FLAG antibody. Anti-actin antibody was used as a native control. (**c**) The transcript levels of PUB30 by RT-qPCR analysis in *pub30 pub31* and wild type or various phosphosite mutated PUB30 complementation lines. Error bars represent SD (n = 3). (**d**) The transcript levels of PUB30 by RT-qPCR analysis of *PUB31* in *pub30 pub31* and wild type or various phosphosite mutated PUB30 complementation lines. Error bars represent SD (n = 3).

We have shown that BAK1-mediated phosphorylation of PUB30 and PUB31 is critical for subsequent association with, and ubiquitination and degradation of ERECTA. Do PUB30/31 phosphorylation events affect their in vivo functions in plant development? To evaluate the contribution of PUB30 T155 and PUB31 T151 phosphorylation on their biological functions, we introduced the phosphomimetic and phosphonull versions of PUB30/31 driven by their native promoters into the *pub30 pub31* mutant. Transgenic plants expressing *proPUB30::PUB30*_*T155A*_ and *proPUB31::PUB31*_*T151A*_ failed to rescue either pedicel growth or stomatal phenotype of *pub30 pub31* (Fig. 5d–g). By contrast, transgenic plants expressing *proPUB30::PUB30*_*T155D*_ and *proPUB31::PUB31*_*T151D*_ fully rescued the mutant phenotypes, both in the context of pedicel growth and stomatal index (Fig. 5d–g). To exclude the possibility that the absence of phenotypic rescues by the phosphonull mutants of PUB30/31 may be attributed to their reduced protein accumulation, we examined the protein expression levels in these transgenic lines. The PUB30_T155A_/31_T151A_ protein and transcript levels are comparable (slightly higher than) with the phosphomimetic or WT versions of PUB30/31 (Extended Data Fig. 9), although they failed to rescue the *pub30 pub31* mutant phenotypes. Collectively, our results highlight that the BAK1-mediated phosphorylation of PUB30 and PUB31 is required for their biological functions in regulating inflorescence/pedicel growth and stomatal development.

## Discussion

**Fig. 6:**
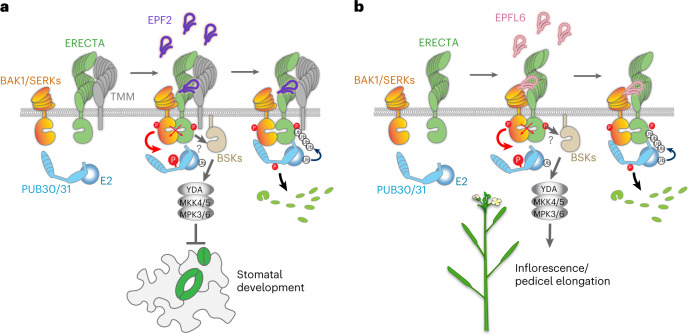
Proposed regulatory mechanisms of ERECTA signal attenuation by PUB30/31 in inflorescence/pedicel growth and stomatal patterning. **a**, Regulation of stomatal development. (Left) ERECTA (green) and TMM (grey) associate in the absence of ligand. (Middle) Upon perception of EPF2 peptide (violet), ERECTA becomes activated and ERECTA and its coreceptor BAK1/SERKs (orange) undergo transphosphorylation events. At the same time, activated BAK1/SERKs phosphorylate PUB30/31 (cyan) at their linker region. The activated ERECTA-BAK1/SERK receptor complex transduces signals most likely (?) via BSK (sand). This leads to activation of a MAPK cascade composed of YODA-MKK4/5-MPK3/6 and inhibition of stomatal development. (Right) The phosphorylated PUB30/31 associate robustly with ERECTA-BAK1/SERKs complex with BAK1/SERKs acting as a scaffold. PUB30/31 ubiquitinate ERECTA (but not BAK1/SERKs) for eventual degradation. The identity of E2 ligase (cyan ball) is unclear. **b**, Regulation of inflorescence/pedicel growth. (Left) ERECTA (green) and BAK1/SERKs (orange) do not associate strongly in the absence of ligand. (Middle) Upon perception of EPFL6 peptide (pink), ERECTA becomes activated, and ERECTA and BAK1/SERKs undergo transphosphorylation events. At the same time, activated BAK1/SERKs phosphorylates PUB30/31 (cyan) at their linker region. The activated ERECTA-BAK1/SERK receptor complex transduces signals most likely (?) via BSK (sand). This leads to activation of a MAPK cascade composed of YODA-MKK4/5-MPK3/6 and promotion of inflorescence and pedicel growth. (Right) Phosphorylated PUB30/31 associate robustly with ERECTA-BAK1/SERKs complex with BAK1/SERKs acting as a scaffold. PUB30/31 ubiquitinate ERECTA (but not BAK1/SERKs) for eventual degradation. The identity of E2 ligase (cyan ball) is unclear.

In this study, we identified a pair of U-box E3 ligases, PUB30 and PUB31, as conserved negative regulators of ERECTA. Our genetic, molecular and biochemical analyses place PUB30/31 as integral components of the regulatory circuit that fine-tunes the ERECTA signalling outputs (Fig. 6). Upon EPF/EPFL ligand perception by ERECTA, ERECTA forms an active receptor complex with BAK1, which directly phosphorylates PUB30/31. This triggers PUB30/31 to ubiquitinate ERECTA, but not BAK1, for degradation (Fig. 6). Following activation, the ERECTA-BAK1 complex relays the signal through BSKs, the MAPK cascade and then the downstream factors to modulate developmental outcomes^23,52,54^. We propose that negative regulation of ERECTA signalling by PUB30/31 ensures robust yet appropriate signalling strengths upon the ligand perception (Fig. 6).

**Extended Data Fig. 10:**
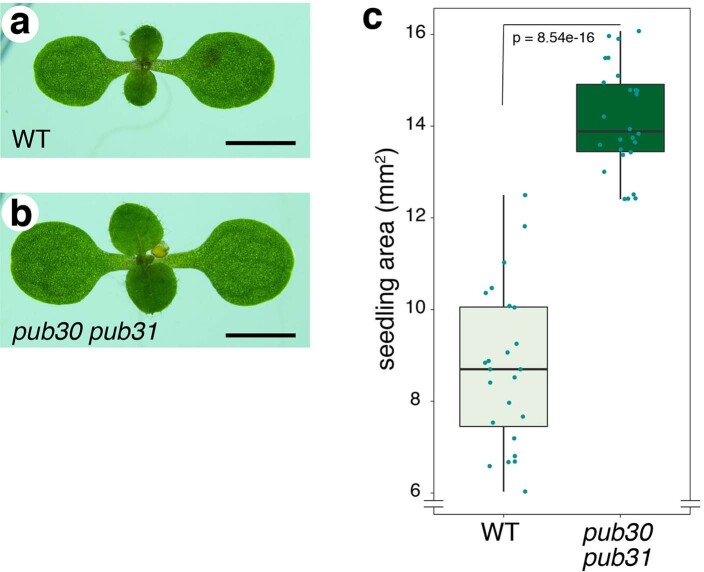
The *pub30 pub31* double mutant seedlings exhibit hyperplasia. (**a, b**) Representative 7-day-old seedlings of wild type (WT, a) and the *pub30 pub31* double mutant (b). Images are taken under the same magnification. Scale bars, 2 mm. (**c**) Quantitative analysis of seedling shoot area from wild-type (WT) and the *pub30 pub31* double mutant. Welch’s two-tailed unpaired t-test was performed (p = 8.54e-16). n = 24 (WT), n = 26 (*pub30 pub31*).

Both ERECTA-mediated inflorescence/pedicel growth and stomatal development are negatively regulated by PUB30 and PUB31 in a largely redundant manner (Figs. 1, 6 and Extended Data Fig. 1). The mutations that abolish the E2-binding (PUB30_W97A_ and PUB31_W93A_) as well as the BAK1-mediated phosphosites (PUB30_T155A_ and PUB31_T151A_) uniformly failed to rescue both extremely elongated pedicel and reduced stomatal index phenotypes of the *pub30 pub31* plants (Figs. 3 and 5). Thus, whereas each EPF/EPFL peptide ligand elicits a unique developmental response via ERECTA, once the ligands are perceived, subsequent processes of signal activation and attenuation are likely conserved. This appears to be the case for the EPF2-mediated inhibition of stomatal development (Fig. 6a) and the EPFL6-mediated elongation of inflorescence and pedicels (Fig. 6b). Nonetheless, the ERECTA receptor complex harbours intricate unequal redundancy: ERECTA-LIKE1 (ERL1) and ERL2, two paralogous receptors, synergistically function with ERECTA and form active receptor complexes with SERK1, SERK2, SERK3/BAK1 and SERK4 (refs. 8, 9,20). In addition to inflorescence/pedicel and stomatal phenotypes, *pub30 pub31* mutant exhibits seedling hyperplasia (Extended Data Fig. 10), suggesting that PUB30/31 may restrict cotyledon and leaf size. Exploring the molecular and biochemical basis of such a phenotype may reveal the roles of these additional paralogues of EPF/EPFL, ERECTA family and BAK1/SERKs family members.

PUB30/31 ubiquitinate the cytoplasmic domain of ERECTA (Extended Data Fig. 2f,g). This explains the previous finding that a truncated ERECTA protein lacking the entire cytoplasmic domain (ERECTAΔK) accumulates at high levels, thereby causing dominant-negative effects^39^. ERECTAΔK is unable to transduce signals (owing to the lack of the kinase domain) or be turned over via PUB30/31-mediated ubiquitination. A more recent study has shown that ERL1, after activation by the EPF1 peptide, undergoes rapid internalization via multivesicular bodies/late endosomes to vacuolar degradation^55^. Again, truncated ERL1 lacking the entire cytoplasmic domain (ERL1ΔK) is stably accumulated at the plasma membrane, irrespective of the ligand perception^55^. It is worth noting that endocytosis of BRI1 relies on the PUB12/13-mediated ubiquitination^31^. Because we found that the inhibitors of clathrin-mediated endocytosis and vacuolar trafficking/degradation inhibit PUB30/31-mediated ERECTA degradation (Fig. 3c and Extended Data Fig. 3b–d), it is likely that ERECTA turnover is coupled with endocytosis.

Our work broadens the roles of PUB proteins as a regulator of receptor kinase signalling and highlights the similarities and differences in their exact mode of actions. We found that BAK1 phosphorylates PUB30/31 at their linker domain (T155 and T151, respectively; Fig. 4). This echoes the idea of phosphorylation as a key activation mechanism of PUB proteins by LRR-RKs and other signalling kinases. For example, MPK3 interacts with and phosphorylates another U-box E3 ligase PUB22 (ref. 56); one of which two phosphosites, T88, lies in the linker domain. BRI1 phosphorylates PUB13 at S344, which also falls in the linker domain^31^. This phosphorylation event subsequently facilitates the turnover of BRI1. Most importantly, we found that BAK1 is required for the association of ERECTA and PUB30/31 as well as further ubiquitination of ERECTA by PUB30/31. Whereas BAK1 phosphorylates and activates PUB30/31, BAK1 itself is not ubiquitinated or subjected to degradation by PUB30/31 (Figs. 4, 5 and Extended Data Fig. 8). The action of BAK1 as a kinase and a scaffold is consistent with its higher binding affinity with PUB30/31 (Fig. 4b,c) and further emphasizes its role as a ‘universal’ coreceptor, allowing the activation and tuning of ligand-perceiving receptors (for example, ERECTA) while maintaining its own homeostasis (Fig. 6). Notably, this scaffold mechanism may also apply to other PUB proteins and their interacting kinases. For example, it has been shown that PUB1, a negative regulator of *Medicago* symbiosis, as well as PUB22 associates with and phosphorylated by signalling kinases, LYK3 (a lysin motif RLK) and MPK3, respectively^56,57^. In either case, however, LYK3 and MPK3 are not ubiquitinated by their interacting PUB proteins^56,57^. Therefore, they may also serve as scaffolds to recruit other components in the signalling pathway to be ubiquitinated by the PUB proteins.

The exact steps of phosphorylation–ubiquitination events differ among the known kinase-PUB signalling modules. For instance, PUB13 ubiquitinates FLS2 upon phosphorylation of PUB13 by BAK1, which in turn strengthens the interaction of PUB13 and FLS2 (ref. 30). By contrast, PUB13 ubiquitinates BRI1 in a BAK1-independent manner^31^. Whereas PUB13 marks elicitor-activated FLS2, PUB25/26 specifically target non-activated BOTRYTIS-INDUCED KINASE1 (BIK1), a receptor-like cytoplasmic kinase, for degradation^58,59^. Overall, the regulatory mechanism of the EPF/EPFL-ERECTA-BAK1-PUB30/31 circuit resembles that of flg22-FLS2-BAK1-PUB12/13 but differs from the ubiquitination of BRI1 and BIK1 by PUB12/13 and PUB25/26, respectively.

Our study revealed the unique aspects of PUB30/31 actions. Besides the phosphorylation–ubiquitination events among the kinase-PUB signalling modules, the phosphorylation status of E3s may also affect protein stability. For instance, phosphorylation of PUB22 by MPK3 stabilizes PUB22 by inhibiting its oligomerization and auto-ubiquitination^56^. By contrast, we found that the phosphorylation of PUB30/31 by BAK1 does not discernibly change their auto-ubiquitination (Extended Data Fig. 7f,g). In addition, the protein levels of phosphonull and phosphomimetic PUB30/31 are consistent with the transcript levels (Extended Data Fig. 9). Thus, our results suggest that the phosphorylation by BAK1 may not be acting as a degron-like signal on PUB30/31. It would be important to note that, structurally, PUB30/31 belong to a different subclade from PUB12/13, and such structural differences may be contributing to the unique mode of PUB30/31 actions. For example, unlike PUB12/13, PU30/31 lack the U-box N-terminal Domain (UND)^37,60^. Furthermore, based on the AlphaFold Structural Database^61^, the PUB30/31 linker region is predicted to adopt a single alpha helix, which is not as flexible as the inker domain in other PUB proteins. This structural feature may account for the low affinity and processivity in vitro of PUB30/31 to ubiquitinate ERECTA. Future biophysical and structural analyses may decipher the specificity and uniqueness of distinct class of PUB proteins in regulating receptor kinase signalling.

Whereas PUB30/31 together regulate ERECTA-mediated developmental processes, PUB30, but not PUB31, has been shown to mediate salt stress tolerance via interacting with and ubiquitinating BRI KINASE INHIBITOR^62^. This raises an important question of what mechanisms govern the target specificities among the PUB proteins. In this regard, it is worth mentioning that PUB12/13 also interact with and ubiquitinate ABA-insensitive 1 (ABI1), an abscisic acid (ABA) coreceptor, which belongs to a protein phosphatase 2C family regulating ABA signalling and drought response^63^. However, PUB12/13 do not ubiquitinate ERECTA, which is structurally resembling to their bona fide target FLS2 rather than ABI^31^. Searching for the interactors and resolving the structural basis of association with otherwise unrelated targets will shed light on the versatile roles of PUB proteins in signal transduction pathways underpinning development and environmental responses.

## Methods

### Plant materials and growth conditions

The *Arabidopsis* accession Columbia (Col) was used as the wild type. All plants used in this study are in a Col background. T-DNA insertion lines for *PUB30* (SALK_012549) and *PUB31* (SALK_054774) were obtained from the Arabidopsis Biological Resource Center. The following mutants and transgenic plant lines have been reported previously: *er-105* (ref. 9), *proERECTA::ERECTA-FLAG* in *er-105* and *proERECTA::ERECTA-YFP* in *er-105* (ref. 19.) *Arabidopsis* seeds were surface sterilized with 30% bleach and grown on half-strength Murashige and Skoog media containing 1× Gamborg Vitamin (Sigma), 0.75% Bacto Agar and 1% w/v sucrose for 9 d and then transplanted into soil. Plants were grown under long-day conditions (16 h light/8 h dark) at 22 °C.

### Plasmid construction and generation of transgenic plants

For recombinant protein expression, the following plasmids were generated: pJA51 (MBP-ER_CD), pCLL107 (GST-PUB30), pCLL109 (GST-PUB31), pCLL223 (GST-PUB30_T155A_), pCLL224 (GST-PUB30_T155D_), pCLL225 (GST-PUB31_T151A_) and pCLL226 (GST-PUB31_T151D_). To construct GST-PUB30 and GST-PUB31, the coding sequences of PUB30 and PUB31 were amplified using Phusion polymerase (ThermoFisher Scientific) and cloned into pGEX-4T-1 using the BamHI and SalI restriction sites. Site-directed mutagenesis was performed using a two-sided polymerase chain reaction (PCR) overlap extension followed by assembly into a linearized vector pGEX-4T-1. For Y2H assays, the coding sequences or domain sequences of the genes of interest were fused to either the DNA-binding domain of the pGBKT7 vector or the AD of the pGADT7 vector using restriction sites digestion and ligation. The following plasmids were generated: pMM213 (BD-ER_CD), pCLL103 (AD-PUB30), pCLL105 (AD-PUB31), pCLL104 (BD-PUB30), pCLL106 (BD-PUB31) and pCLL143 (BD-BAK1_CD). For complementation assays, the following plasmids were generated: pCLL124 (*proPUB30::PUB30-YFP*), pCLL126 (*proPUB31::PUB31-YFP*), pCLL123 (*proPUB30::PUB30-FLAG*), pCLL125 (*proPUB31::PUB31-FLAG*), pCLL176 (*proPUB30::PUB30*_*W97A*_*-FLAG*), pCLL178 (*proPUB31::PUB31*_*W93A*_*-FLAG*), pCLL235 (*proPUB30::PUB30*_*T155A*_*-FLAG*), pCLL236 (*proPUB30::PUB30*_*T155D*_*-FLAG*), pCLL237 (*proPUB31::PUB31*_*T151A*_*-FLAG*) and pCLL238 (*proPUB31::PUB31*_*T151D*_*-FLAG*). A PCR-based Gateway system was used to generate these constructs. The promoter regions (3 kb) of *PUB30* and *PUB31* were amplified and subcloned into the pENTR-5′-TOPO cloning vector (ThermoFisher Scientific). The *PUB30* and *PUB31* (WT or mutant) sequences were amplified and subcloned into the pKUT612 cloning vector using restriction enzyme digestion and T4 ligation. A three-way Gateway system^64^ was utilized to generate a series of PUB30 and PUB31 constructs driven by the respective promoters. See Supplementary Tables 2 and 3 for details of plasmid and primer sequence information. Plasmids are transformed into *Agrobacterium* GV3101/pMP90 and subsequently to *Arabidopsis* by floral dipping. More than ten lines were characterized for the phenotypes and reporter gene expressions.

### Quantitative PCR with reverse transcription analysis

RNA extraction, cDNA synthesis and quantitative PCR with reverse transcription (RT-qPCR) were performed as previously described^65^. For a list of primers, see Extended Data Table 3.

### Histological analysis and microscopy

For histological analysis, mature pedicels were fixed, dehydrated and embedded in polymethacryl resin Technovit 7100 (Heraeus Kulzer) as described previously^39^. Tissue sections were prepared using a Leica RM-6145 microtome, and tissue sections were stained with 0.1% toluidine blue (Sigma) in 0.1 M NaPO_4_ buffer (pH 7.0) and observed under Olympus BX40 light microscope. Confocal microscope images were taken using either a Zeiss LSM700 operated by Zen2009 (Zeiss) as described previously^19^ or a Leica SP5-WLL operated by LAS AF (Leica). Cell peripheries of seedlings were visualized with propidium iodide (Molecular Probes). Images were taken with excitation at 514 nm and emission at 518–600 nm for YFP, and excitation at 619 nm and emission at 642 nm for propidium iodide. For Lecia SP5 confocal microscope, a HyD detector was used. The confocal images were false coloured, and brightness/contrast were uniformly adjusted using Photoshop 2021 (Adobe). The Z-stack projection images were taken at an interval of 0.99 µm, covering the thickness of the entire cotyledon.

### Quantitative analysis and statistics

For analysis of epidermis, abaxial cotyledons from 10-, 6- or 8-d-old seedlings of respected genotypes were subjected to propidium iodide staining and confocal microscopy. The central regions overlying the distal vascular loop were imaged and numbers of epidermal cells, stomata and their cluster size were quantified. Pedicel lengths were measured using ImageJ. Statistical analysis was performed using R v.4.1.0 operated under R-Studio v.1.4.1717 (https://www.rstudio.com), and graphs were generated using the R ggplot2 package. For all boxplots, each box represents interquartile range, in which the top, middle and bottom lines indicate the 75, 50 and 25 percentiles, respectively, and the bar represents maximum to minimal values. Each dot represents a jittered individual data point. Data points that go beyond the bar are outliers. Violin plots (Fig. 1e) show the data and probability density distribution with individual data points overlaid as jittered dots. For all analyses, Welche’s unpaired *t*-test or Student’s *t*-test were performed for a pairwise comparison, and one-way analysis of variance (ANOVA) followed by Tukey’s HSD test were performed for comparison of groups. For individual sample size (*n*) and *P* values, see the corresponding figures or figure legends.

### Yeast two-hybrid assay

Bait and prey constructs were cotransformed into the yeast strain AH109 using the yeast transformation kit (Frozen-EZ Yeast Transformation II Kit, Zymo Research). The resulting transformants with appropriate positive and negative controls were spotted on SD (−Leu, −Trp) plates to check for growth in the absence of selection. Transformants were then spotted on SD (−Trp, −Leu, −His) selection media containing 1 mM 3-amino-1,2,4-triazole (3-AT, A8056, Sigma). The positive interactors were then scored based on the stringency of the selection.

### Expression, purification and refolding of peptides

Recombinant MEPF2 and MEPFL6 peptides were prepared as described previously^19^. The bioactivities of refolded peptides were confirmed as described previously^19^.

### Co-IP, protein gel electrophoresis and immunoblots

For co-IP assays of ERECTA and PUB30/31 with seedlings, *Arabidopsis* transgenic lines expressing different combinations of *proERECTA::ERECTA-YFP*, *Lti6B-GFP*, *proPUB30::PUB30-FLAG* and *proPUB31::PUB31-FLAG* were generated. For co-IP assays of BAK1 and PUB30/31 with seedlings, *Arabidopsis* transgenic lines expressing *Lti6B-GFP*, *proPUB30::PUB30-YFP* and *proPUB31::PUB31-YFP* were generated. For peptide treatment, *Arabidopsis* seedlings were grown for 5 d on half-strength Murashige and Skoog media plates and then transferred to double-distilled H_2_O for 24 h. Seedlings were first treated with 50 µM MG132 (M7449, Sigma) for 3 h. Thereafter, further treatment was performed with Tris–HCl (pH 8.0, 50 mM) buffer only, MEPF2 (2.5 µM) or MEPFL6 (2.5 µM) at room temperature for another 3 h before being pooled for harvest and then subjected to protein preparation.

Samples were ground in liquid nitrogen and homogenized in the extraction buffer (100 mM Tris–HCl pH 8.8, 150 mM NaCl, 1 mM EDTA, 20% glycerol, 1 mM phenylmethyl sulfonyl fluoride, 1× cOmplete Protease Inhibitor Cocktail (Roche), 1× phosphatase inhibitor cocktail 2 and 3 (Sigma)). The slurry was centrifuged at 10,000*g* for 15 min at 4 °C. The supernatant was sonicated on ice and then centrifuged at 100,000*g* for 30 min at 4 °C to yield microsomal fractions. The pellet was resuspended in membrane solubilization buffer (100 mM Tris–HCl at pH 7.3, 150 mM NaCl, 1 mM EDTA, 10% glycerol, 1% Triton X-100, 1 mM phenylmethyl sulfonyl fluoride, 1× cOmplete Protease Inhibitor Cocktail (Roche), 1× phosphatase inhibitor cocktail 2 and 3 (Sigma)) to release membrane proteins. The solution was sonicated on ice and centrifuged again at 100,000*g* for 30 min at 4 °C. The supernatant was incubated with protein G-coupled magnetic beads (Dynabeads Protein G; Invitrogen) that captured anti-FLAG (Abcam, catalogue no. ab205606) antibody at 4 °C for 2 h with gentle agitation. The beads were washed four times with 500 μl of phosphate buffer (pH 7.4) and precipitated proteins were eluted with 4× SDS sample buffer at 95 °C for 5 min. Either total membrane or immunoprecipitated proteins were separated on an SDS–polyacrylamide gel electrophoresis (SDS–PAGE) gel and transferred to a polyvinylidene difluoride (PVDF) membrane (Millipore) for immunoblot analysis using monoclonal anti-GFP (1:1,000; ThermoFisher Scientific, catalogue no. 33-2600), anti-FLAG (1:5,000; Sigma, catalogue no. F-3165) and anti-BAK1 (1:5,000; Agrisera, catalogue no. AS12 1858) as primary antibodies. As secondary antibodies, sheep anti-mouse IgG horseradish peroxidase-linked antibody (GE Healthcare, catalogue no. NA931) and goat anti-rabbit IgG (whole molecule)–peroxidase antibody (Sigma, catalogue no. A6154) were used at a dilution of 1:50,000 and 1:6,000, respectively. The protein blots were visualized using Chemi-luminescence assay kit (ThermoFisher Scientific, catalogue no. 34095) and imaged using a software, Image Lab (v.6.0.1; Bio-Rad).

For co-IP assays with *Arabidopsis* protoplasts, protoplasts were transfected with haemagglutinin (HA)-tagged ERECTA and MYC-tagged PUB30 or PUB31 (WT or various phosphor-mutated) and incubated for 8 h. Protoplasts were then pretreated with 2 µM MG132 (Sigma, catalogue no. M7449) for 1 h, followed by treatment with 5 µM EPFL6 for 1 h. The total proteins were isolated with extraction buffer (50 mM Tris–HCl, pH 7.5, 150 mM NaCl, 1 mM EDTA, 20% glycerol, 1% Triton X-100, and 1× cOmplete protease inhibitor cocktail (Roche), 1× phosphatase inhibitor cocktail 2 and 3 (Sigma)). The supernatant was incubated with protein G-coupled magnetic beads (Dynabeads Protein G; Invitrogen) that captured anti-MYC (Abcam, catalogue no. ab9106) antibody at 4 °C for 2 h with gentle agitation. The beads were then washed three times with 500 μl of wash buffer (50 mM Tris–HCl, pH 7.5, 150 mM NaCl, 1 mM EDTA, 20% glycerol, 0.2% Triton X-100 and 1× cOmplete protease inhibitor cocktail (Roche), 1× phosphatase inhibitor cocktail 2 and 3 (Sigma)) and precipitated proteins were eluted with 4× SDS sample buffer at 95 °C for 5 min. Either total or immunoprecipitated proteins were separated on a SDS–PAGE gel and transferred to PVDF membrane (Millipore) for immunoblot analysis using anti-HA (1:1,000; Abcam, catalogue no. ab18181) and anti-MYC (1:1,000; Abcam, catalogue no. ab32) as primary antibodies. As secondary antibody, goat anti-mouse IgG H&L (HRP) (Abcam, catalogue no. ab205719) was used at a dilution of 1:5,000. The protein blots were visualized as described in the previous paragraph.

### In vitro pull-down assay

For pull-down assays of PUB30/31 and ER_CD or BAK1_CD, ~15 μg of GST-PUB30 or GST-PUB31 or GST proteins were incubated with about 15 μg MBP-ERECTA_CD or MBP-BAK1_CD or MBP proteins in 900 μl of pull-down buffer (10% glycerol, 1% Triton X-100, 1.5 mM MgCl_2_, 150 mM NaCl, 1 mM EGTA, 50 mM HEPES, pH 7.5, 1 mM phenylmethyl sulfonyl fluoride and 1× cOmplete protease inhibitor cocktail (Roche)). For pull-down assay of PUB30/31 phosphomimetic and WT versions with BAK1_CD, ~15 μg of GST-PUB30T155D or GST-PUB30 or GST-PUB31T151D or GST-PUB31 or GST proteins were incubated with ~15 μg of MBP-BAK1_CD protein in 900 μl of pull-down buffer. For these pull-down assays, 30 μl of GST beads (Glutathione Sepharose 4 Fast Flow; Cytiva, catalogue no. 17-5132-01) were incubated with each reaction mixture with gentle shaking at 4 °C for ~1 h.

For pull-down assay of PUB30/31 phosphomimetic and WT versions with ER_CD, ~15 μg of GST-PUB30T155D or GST-PUB30 or GST-PUB31T151D or GST-PUB31 or GST proteins were incubated with ~15 μg of MBP-ERECTA_CD protein in 900 μl of pull-down buffer. For pull-down assay of PUB30/31 with BAK1_CD and BAK1_CD mut, ~15 μg of GST-PUB30 or GST-PUB31 proteins were incubated with ~15 μg of MBP-BAK1_CD or MBP-BAK1_CD mut or MBP proteins in 900 μl of pull-down buffer. For these pull-down assays, 30 μl of MBP beads (amylose resin; New England Biolabs, catalogue no. E8021S) were incubated with each reaction mixture with gentle shaking at 4 °C for ~1 h.

After the reaction, beads were washed three times and heated for 5 min in a 95 °C metal bath. The immunoprecipitated proteins were separated by SDS–PAGE and detected by anti-GST (1:5,000; GenScript, catalogue no. A00865-200) and anti-MBP (1:10,000; New England Biolabs, catalogue no. E8032) antibodies, respectively. As a secondary antibody, sheep anti-mouse IgG horseradish peroxidase-linked antibody (GE Healthcare, catalogue no. NA931) was used at a dilution of 1:50,000. The protein blots were visualized as described in the previous section.

### In vitro and in vivo ubiquitination assays

The in vitro ubiquitination reactions contained 1 μg each of MBP-ERECTA_CD, HIS6-E1 (AtUBA1), HIS6-E2 (AtUBC8), HIS6-ubiquitin (Boston Biochem) and GST-PUB30 or GST-PUB31 in the ubiquitination reaction buffer (0.1 M Tris–HCl, pH 7.5, 25 mM MgCl_2_, 2.5 mM dithiothreitol and 10 mM ATP; final volume 30 μl). The reactions were incubated at 30 °C for 3 h, stopped by adding SDS sample loading buffer and boiled at 95 °C for 5 min. The samples were then separated by 8% SDS–PAGE, and the ubiquitinated ERECTA_CD were detected by immunoblot analysis with anti-MBP (1:10,000; New England Biolabs, catalogue no. E8032) as primary antibody, whereas the auto-ubiquitination was detected by immunoblot analysis with anti-GST (1:5,000; GenScript, catalogue no. A00865-200) as the primary antibody. As secondary antibody, sheep anti-mouse IgG horseradish peroxidase-linked antibody (NA931, GE Healthcare) was used at a dilution of 1:50,000.

For in vivo ubiquitination assays, *Arabidopsis* protoplasts were cotransfected with FLAG-tagged ubiquitin (FLAG-UBQ), HA-tagged ERECTA and together with a control vector or MYC-tagged PUB30 or PUB31 (WT or various phosphor-mutated) and incubated for 8 h followed by treatment with 5 µM EPFL6 for 1 h in the presence of 2 µM MG132 (Sigma, catalogue no. M7449). The ubiquitinated ERECTA was detected with anti-HA (1:1,000; Abcam, catalogue no. ab18181) immunoblotting after immunoprecipitation with anti-FLAG (Abcam, catalogue no. ab205606) antibody. The total ubiquitinated proteins were detected by anti-FLAG (1:5,000; Sigma, catalogue no. F-3165) and anti-MYC (1:1,000; Abcam, catalogue no. ab32) as primary antibodies. As secondary antibody, sheep anti-mouse IgG horseradish peroxidase-linked antibody (GE Healthcare, catalogue no. NA931) was used at a dilution of 1:50,000. The protein blots were visualized as described in the previous section.

### In vitro kinase assay using isotope

Kinase assays were conducted in 30-µl reactions containing 20 mM Tris–HCl (pH 7.5), 5 mM EGTA, 1 mM dithiothreitol, 100 mM NaCl, 10 mM MgCl_2_, 100 µM [γ-^32^P]ATP mix (5 µCi of ATP; ATP, [γ-^32^P]-3000 Ci mmol^−1^, 10 mCi ml^−1^; EasyTide, 100 µCi), 10 μg of substrate proteins and 1 μg of kinases.

The reactions were incubated at 30 °C for 30 minutes and stopped with the SDS sample buffer. After SDS–PAGE, gels were dried, exposed to a GE multipurpose standard screen (catalogue no. 63-0034-87) for 18 h and imaged using a GE Typhoon FLA 9000 gel imager.

### Mass spectrometry and identification of the phosphosites

For the in vitro phosphorylation reaction, 1 µg of MBP-BAK1_CD protein was incubated with 10 µg of GST-PUB30 in 30 µl of kinase reaction buffer at 30 °C for 3 h (with gentle shaking). After the reaction, the SDS loading buffer was used to stop the kinase reactions. Samples were separated by 10% SDS–PAGE. The gels were fixed for 60 min in 50% methanol + 7% acetic acid, rinsed thoroughly with Milli Q water and stained with GelCode Blue Stain Reagent (ThermoFisher Scientific, catalogue no. 24590). Target bands for GST-PUB30 are cut off from the electrophoresis gel and digested using chymotrypsin (Sigma) at 37 °C overnight. To analyse the chymotryptic peptides, nano-flow reverse-phase liquid chromatography and tandem mass spectrometry was performed using a Q Exactive Hybrid Quadrupole-Orbitrap Mass Spectrometer (ThermoFisher Scientific) as described previously^66^. Subsequently, peptide identification was performed by searching the *Arabidopsis thaliana* reference genome (downloaded from https://www.uniprot.org) using the SEQUEST (ThermoFisher Scientific) search engine. The parameter of dynamic modifications with phosphorylation filter was added for the identification of phosphopeptides. Details of the peptide spectrum match with phosphorylated residues were validated manually and annotated in Thermo Proteome Discoverer v.2.4.

### In vivo phosphorylation assay

*Arabidopsis* protoplasts were cotransfected with HA-tagged BAK1 (BAK1-HA), and MYC-tagged WT and phosphonull versions of PUB30 or PUB31 and incubated for 8 h followed by treatment with 5 μM EPFL6 for 1 h in the presence of 2 μM MG132 (M7449, Sigma).

After immunoprecipitation using anti-MYC beads, phosphorylated PUB30/31 (WT and phosphonull versions) was detected with anti-pThr antibody (1:1,000; Cell Signaling Technology, catalogue no. 9386S) as the primary antibody. The input PUB30 or PUB31 proteins and BAK1 were detected with anti-MYC antibody (1:1,000; Abcam, catalogue no. ab32) and anti-HA antibody (1:1,000; Abcam, catalogue no. ab18181) as primary antibodies, respectively. As secondary antibody, goat anti-mouse IgG H&L (HRP) (Abcam, catalogue no. ab205719) was used at a dilution of 1:5,000. The protein blots were visualized as described in the previous section.

### Peptide and inhibitor treatment and immunoblot assays

The *proERECTA::ERECTA-FLAG er-105* and *proERECTA::ERECTA-FLAG pub30 pub31 er-105* seedlings were grown vertically at 22 °C on half-strength Murashige and Skoog medium for 7 d. Thereafter, different treatments were further performed. For the endocytosis inhibitor treatment, seedlings were incubated with or without 50 μM Tyrphostin A23 (Tyr A23) (Sigma, catalogue no. T7165) for 30 min. For the vacuolar ATPase inhibitor treatment, seedlings were incubated with or without 1 μM Con A (Abcam, catalogue no. ab144227) for 30 min. For peptides EPF2/EPFL6 treatment, seedlings were incubated with or without 5 μM peptides for 180 min. Inactive (boiled at 70 °C for 30 min before use) EPF2/EPFL6 were used as negative controls.

Total protein extracts were separated on a 10% SDS–polyacrylamide gel and detected by immunoblot analysis with anti-FLAG (1:5,000; Sigma, catalogue no. F-3165) and anti-actin (1:2,000; Abcam, catalogue no. ab230169) as primary antibodies. As secondary antibody, goat anti-mouse IgG H&L (HRP) (Abcam, catalogue no. ab205719) was used at a dilution of 1:5,000. Protein blots were visualized as described in the previous section.

### Biolayer interferometry

The binding affinities of the ERECTA_CD with GST-tagged PUB30 and PUB31 were measured using the Octet Red96 system (ForteBio, Pall Life Sciences) following the manufacturer’s protocols. The optical probes coated with anti-GST were first loaded with 2,000 nM GST-PUB30 or PUB31 before kinetic binding analyses. The experiment was performed in 96-well plates maintained at 30 °C. Each well was loaded with 200 μl of reaction volume, and the binding buffer used in these experiments contained 1× PBS (pH 7.2) supplemented with 0.02% Tween 20. The concentrations of the ERECTA_CD as the analyte in the binding buffer were 20,000, 10,000, 5,000, 2,500, 1,250, 625 and 312.5 nM. Similarly, for the binding of BAK1-CD with GST-PUB30/31, the optical probes coated with anti-GST were first loaded with 1,000 nM GST-PUB30 or PUB31 before kinetic binding analyses. The concentrations of the BAK1_CD as the analyte in the binding buffer were 5,000, 2,500, 1,250, 625, 312.5, 156.3 and 78.2 nM. All preformed complexes remained stable as suggested by the constant signal during the washing step after loading. There was no binding of the analytes to the unloaded probes as shown by the control wells. Binding kinetics to all seven concentrations of the analytes were measured simultaneously using default parameters on the instrument. The data were analysed using the Octet data analysis software. The association and dissociation curves were fit with the 1:1 homogeneous ligand model. The *k*_obs_ (observed rate constant) values were used to calculate dissociation constant (*K*_d_), with steady-state analysis of the direct binding.

### ERECTA protein stability in protoplasts

To determine ERECTA protein stability, protoplasts cotransfected with ERECTA-HA and phosphor-mutant and WT versions of PUB30/31-MYC were treated with 50 μM cycloheximide (Sigma, catalogue no. C4859) in the presence or absence of 5 μM EPFL6 for 3 h. Total proteins were separated on SDS–PAGE gels and transferred to a PVDF membrane (Millipore) for immunoblot analysis. ERECTA protein and the input PUB30 or PUB31 proteins were detected with anti-HA antibody (1:1,000; Abcam, catalogue no. ab18181) and anti-MYC antibody (1:1,000; Abcam, catalogue no. ab32) and as primary antibodies, respectively. As secondary antibody, goat anti-mouse IgG H&L (HRP) (Abcam, catalogue no. ab205719) was used at a dilution of 1:5,000. Protein blots were visualized as described in the previous section.

### Reporting summary

Further information on research design is available in the Nature Portfolio Reporting Summary linked to this article.

## Supplementary information

Supplementary Information Extended Data table of contents, figures and figure legends.

Reporting Summary

Supplementary Tables 1–3 Table 1: Mass spectra detecting the PUB30 T155 phosphorylation. Table 2: List of plasmids used in this study. Table 3: List of primers used in this study.

Supplementary Data Extended dataset *P* values for one-way ANOVA–Tukey’s HSD test.

## Data Availability

All generated and analysed data from this study are included in the main figures, Extended Data figures and supplementary information. Source data are provided with this paper.

## Source data

Source Data Fig. 1 All uncropped gel and blot images for all figures.
