# Supplementary material for: Direct attenuation of Arabidopsis ERECTA signalling by a pair of U-box E3 ligases
Source: Nat Plants. 2022 Dec 20;9(1):112–27. doi: 10.1038/s41477-022-01303-x (PMC9873567; doi:10.1038/s41477-022-01303-x)
Supplement: Supplementary file 1 — Extended Data table of contents, figures and figure legends. [file 41477_2022_1303_MOESM1_ESM.docx]

**Direct attenuation of Arabidopsis ERECTA signaling by a pair of U-box E3 ligases**

Liangliang Chen, Alicia M. Cochran, Jessica M. Waite, Ken Shirasu, Shannon M. Bemis, Keiko U. Torii

**SUPPLEMENTARY TABLE OF CONTENTS**

**Extended Data Tables 1-3**

**Extended Data Table 1.** Mass spectra detecting the PUB30 T155 phosphorylation

**Extended Data Table 2.** List of plasmids used in this study

**Extended Data Table 3.** List of primers used in this study

**Extended Dataset 1.** List of p values for all One-way ANOVA -Tukey's HSD tests

**Source File.** Uncropped Gel Blot images

**Extended Data Figures 1-10**

**Extended Data Fig. 1.** Analysis of *pub30* and *pub31* T-DNA mutants and genetic complementation of *pub30 pub31*.

**Extended Data Fig. 2.** Subcellular localization, domain structures, interaction, and ubiquitination assays of PUB30/31.

**Extended Data Fig. 3.** Regulation of ERECTA protein abundance and specific pathways involved.

**Extended Data Fig. 4.** Domain interaction analysis of PUB30/31 with ERECTA and BAK1 in yeast.

**Extended Data Fig. 5.** PUB30/31 interact with BAK1 but not with downstream BSK1 or YODA.

**Extended Data Fig. 6.** BAK1 phosphorylates PUB30 and PUB31 in the linker domain.

**Extended Data Fig. 7.** Phosphorylation of PUB30 and PUB31 by BAK1 is required for their association with and ubiquitination of ERECTA.

**Extended Data Fig. 8.** BAK1 is not a substrate of PUB30/31.

**Extended Data Fig. 9.** Protein and transcript levels of phosphonull and phosphomimetics PUB30/31 in Arabidopsis seedlings.

**Extended Data Fig. 10.** The *pub30 pub31* double mutant seedlings exhibit hyperplasia.

**
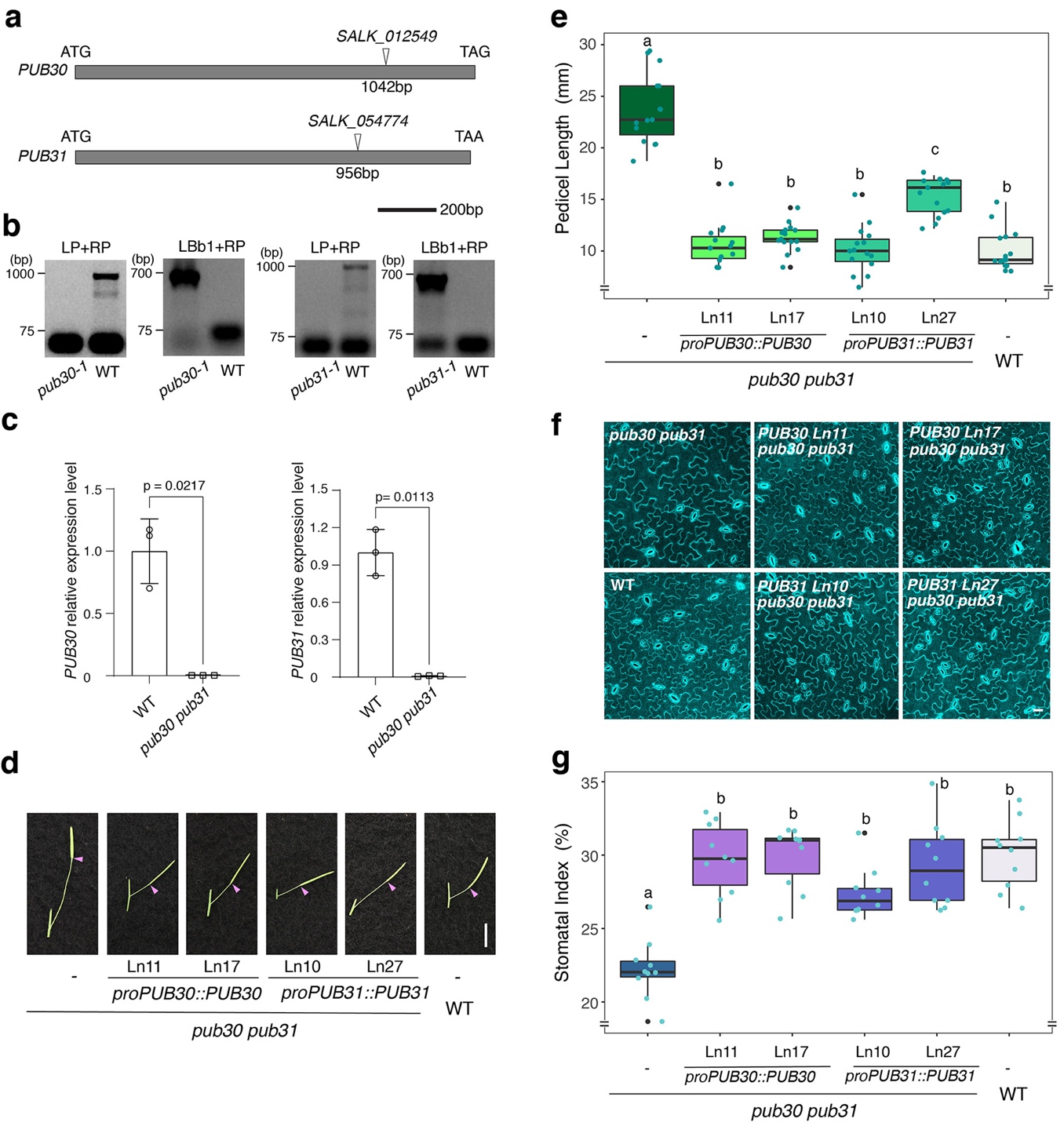
**

**Extended Data Fig. 1. Analysis of *pub30* and *pub31* T-DNA mutants and genetic complementation of *pub30 pub31* mutant phenotypes.**

(a) Schematic diagrams of T-DNA insertion sites in *pub* mutants. The start codon (ATG) and the stop codon (TAG/ TAA) are indicated. Scale bar: 200 bp

(b) PCR identification of the T-DNA insertion in *pub30-1* and *pub31-1* with T-DNA specific primers (LBb1) and flanking primers (LP and RP). These alleles are referred as *pub30* and *pub31*, respectively.

(c) qRT-PCR analysis of *PUB30* and *PUB31* in WT and *pub30 pub31* plants. Error bars represent SD (n = 3). Two-tailed paired Student’s t-test was performed. p values are in the graph.

(d) Representative pedicels of mature siliques of *pub30 pub31*, *proPUB30::PUB30*; *pub30 pub31* Ln 11and 17, *proPUB31::PUB31*; *pub30 pub31* Ln 10and 27, and wild type plants. Scale bar: 1 cm

(e) Morphometric analysis of pedicel length from each genotype. 6-wk-old mature pedicels (n = 15) were measured. One-way ANOVA followed by Tukey’s HSD test was performed for comparing all other genotypes and classify their phenotypes into three categories (a, b, and c). For p values see Extended Dataset S1.

(f) Confocal microscopy of 8-d-old abaxial cotyledon epidermis of *pub30 pub31*, *proPUB30::PUB30*; *pub30 pub31* Ln 11and 17, *proPUB31::PUB31*; *pub30 pub31* Ln 10 and 27, and wild type. Scale bar: 25 μm

(g) Quantitative analysis. Stomatal index (SI) of the cotyledon abaxial epidermis from 8-day-old seedlings of respective genotypes (n = 10). One-way ANOVA followed by Tukey’s HSD test was performed for comparing all other genotypes and classify their phenotypes into two categories (a and b). For p values see Extended Dataset S1.


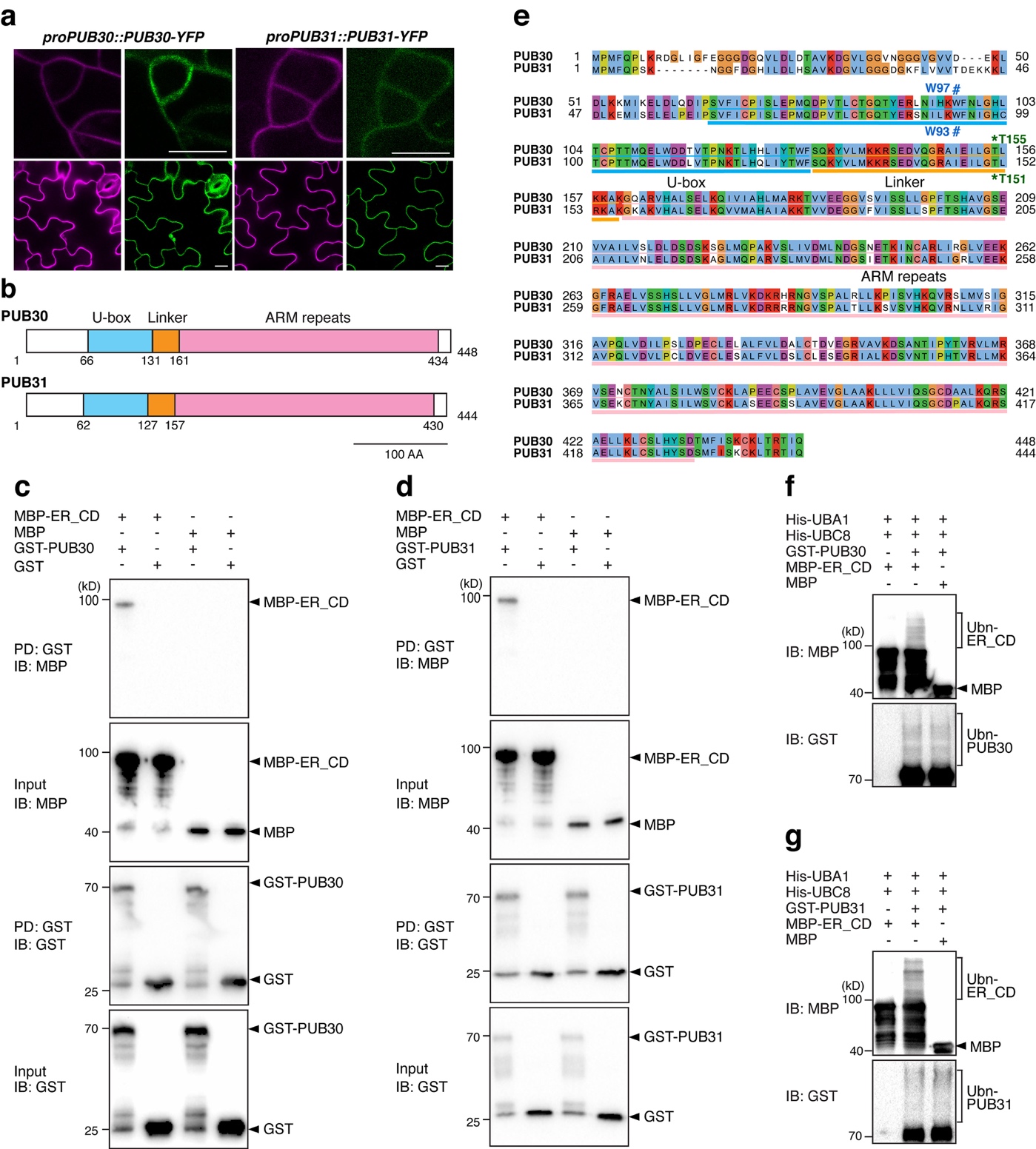


E**xtended Data Fig. 2. Subcellular localization, domain structures, interaction, and ubiquitination assays of PUB30/31.**

(a) PUB30/31-YFP signals could be detected at the PM in both meristemoids and pavement cells. Confocal microscopy of 6-d-old abaxial cotyledon epidermis of *proPUB30::PUB30-YFP*; *pub30 pub31* or *proPUB31::PUB31-YFP*; *pub30 pub31 transgenic lines.* Scale bar, 10 µm

(b) Schematic diagram of the domains of PUB30 and PUB31. PUB30 and PUB31 contain a U-box domain, a linker domain, and an ARMADILLO (ARM) repeat domain. The light blue, orange and pink color rectangles indicate the U-box domain, the linker domain, and the ARM repeats domain, respectively. Scale bar: 100 aa.

(c) PUB30 interacts with ER_CD *in vitro*. MBP-ER_CD was pulled down (PD) by GST-PUB30 immobilized on Glutathione Sepharose 4B and analyzed by immunoblotting (IB) using an anti-MBP antibody. GST and MBP were used as negative controls.

(d) PUB31 interacts with ER_CD *in vitro*.

(e) The alignment of PUB30 and PUB31. Protein sequences above light blue lines, orange lines, and pink lines represent U-box domain, linker domain, and ARM repeats domain, respectively. The amino acid position of PUB30 is labeled on the top. The Tryptophan 97 (W97) site in PUB30 and the conserved W93 site in PUB31 are labeled with #. The Threonine 155 (T155) site in PUB30 and the conserved T151 site in PUB31 are labeled with *.

(f) PUB30 ubiquitinates ERECTA *in vitro*. The ubiquitination of MBP-ER_CD was carried out by using GST-fused PUB30 as the E3 ligase, His-fused AtUBA1 as E1 activating enzyme, and His-fused UBC8 as E2 conjugating enzyme. MBP was used as a control.

(g) PUB31 ubiquitinates ERECTA *in vitro*.

**
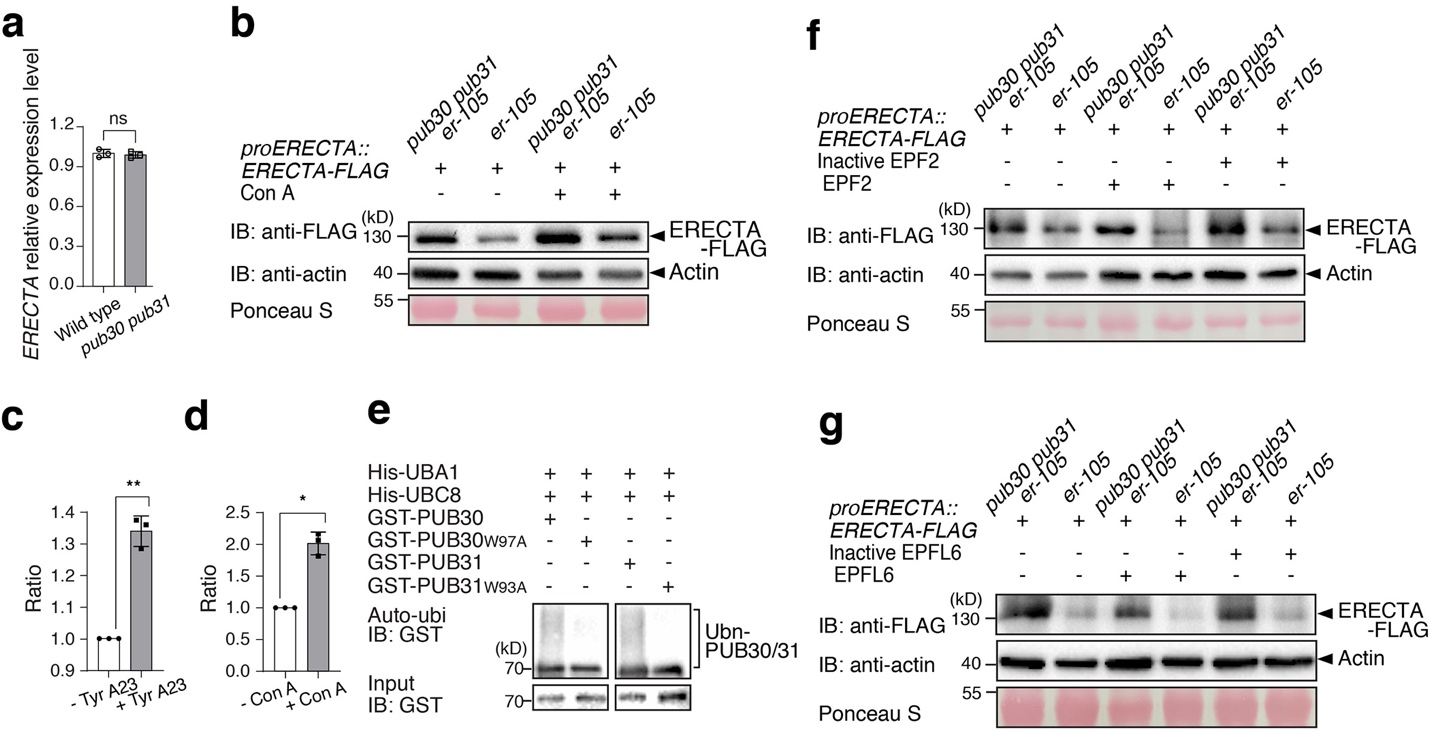
**

**Extended Data Fig. 3. Regulation of ERECTA protein abundance and specific pathways involved.**

(a) qRT-PCR analysis of *ERECTA* in wild type and *pub30 pub31* plants. Error bars represent SD (n = 3). Two tailed paired Student’s t-test was performed (p = 0.7073). ns, not significant.

(b) ERECTA protein accumulation in wildtype, and *pub30 pub31*, in the absence and presence of the vacuolar ATPase inhibitor Concanamycin A (Con A). Total proteins were isolated from 7-d-old seedlings and probed by an α-FLAG antibody. The protein inputs were equilibrated using α-Actin antibodies.

(c) Quantification of ERECTA abundance (ERECTA/Actin) in the absence and presence of Tyr A23. Error bars represent SD (n = 3). The asterisks indicate statistical significance by using two-tailed paired Student’s t-test ( p = 0.0067).

(d) Quantification of ERECTA abundance (ERECTA/Actin) in the absence and presence of Con A. Error bars represent SD (n = 3). The asterisks indicate statistical significance by using two-tailed paired Student’s t-test (p = 0.0102).

(e) The residues (W97 in PUB30 and W93 in PUB31) are essential for their autoubiquitination, respectively. The *in vitro* autoubiquitination assays were performed using GST-PUB30/31 wild type or mutants as the E3 ligases.

(f) ERECTA protein accumulation in wildtype, and *pub30 pub31*, in the absence and presence of the EPF2 and inactive EPF2. Total proteins were isolated from 7-d-old seedlings and probed by an α-FLAG antibody. The protein inputs were equilibrated using α-Actin antibodies.

(g) ERECTA protein accumulation in wildtype, and *pub30 pub31*, in the absence and presence of the EPFL6 and inactive EPFL6. Total proteins were isolated from 7-d-old seedlings and probed by an α-FLAG antibody. The protein inputs were equilibrated using α-Actin antibodies.


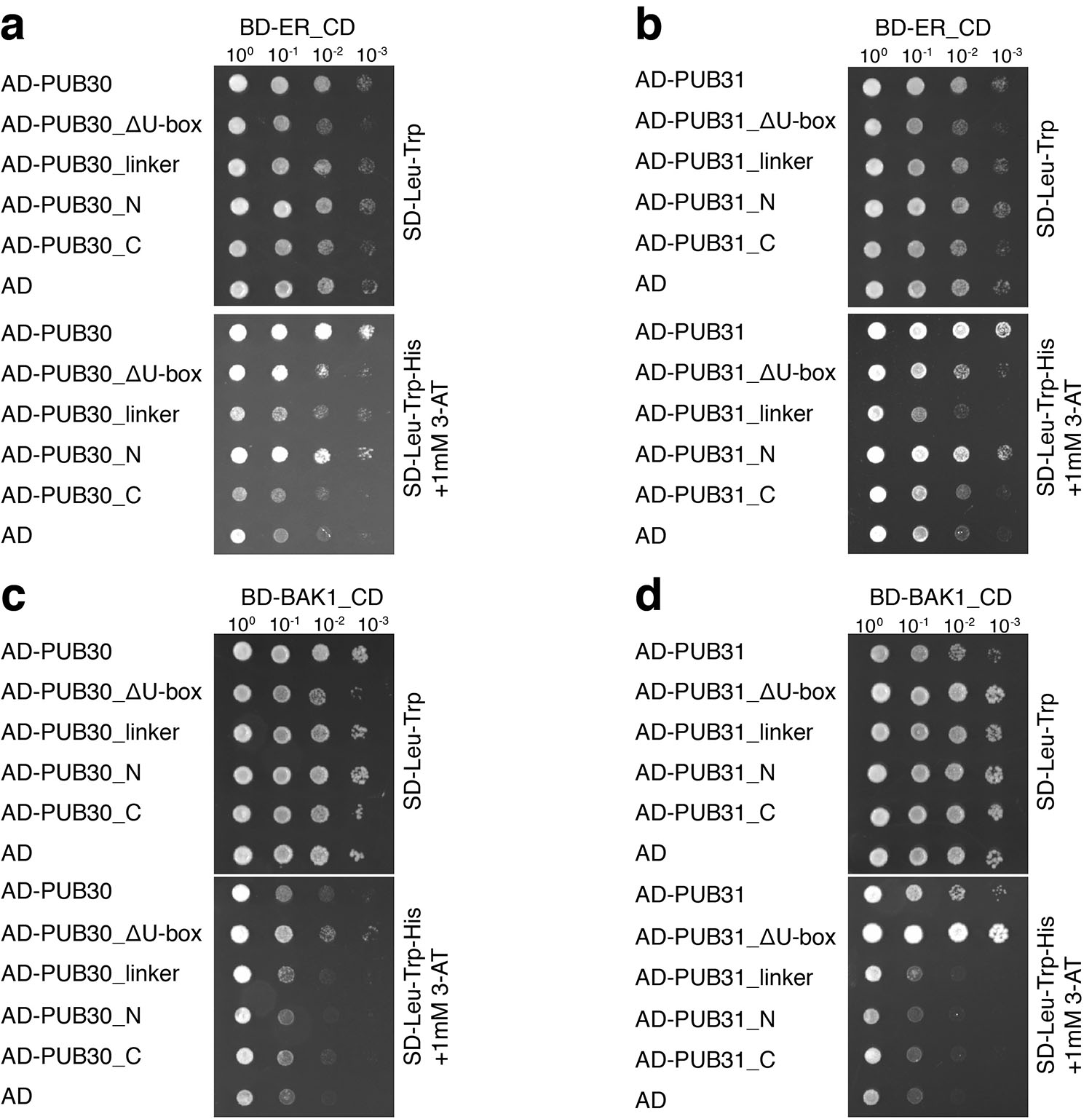


**Extended Data Fig. 4. Domain interaction analysis of PUB30/31 with ERECTA and BAK1 in yeast.**

(a) The U-box domain of PUB30 is not essential for its interaction with ERECTA in yeast. Whole length and different regions of PUB30 were used as baits. ER_CD and AD alone (as a negative control) were used as prey. Yeast clones were spotted in 10-fold serial dilutions on appropriate selection media. The experiment was repeated independently three times with similar results. For the constructs used here: PUB30 (1-65+132-448) is PUB30 (ΔU-box). PUB30 (132-200) contains the linker domain. PUB30 (1-200) is PUB30_N, which contains the U-box domain and linker domain. PUB30 (168-448) is PUB30_C, which does not contain U-box or linker domain.

(b) U-box domain of PUB31 is not essential for its interaction with ERECTA in yeast. For the constructs used here: PUB31 (1-61+128-444) is PUB31 (ΔU-box). PUB31 (128-200) contains the linker domain. PUB31 (1-200) is PUB31_N, which contains the U-box domain and linker domain. PUB31 (168-444) is PUB31_C, which does not contain U-box or linker domain.

(c) The U-box domain of PUB30 is not essential for its interaction with BAK1 in yeast.

(d) The U-box domain of PUB31 is not essential for its interaction with BAK1 in yeast.

**
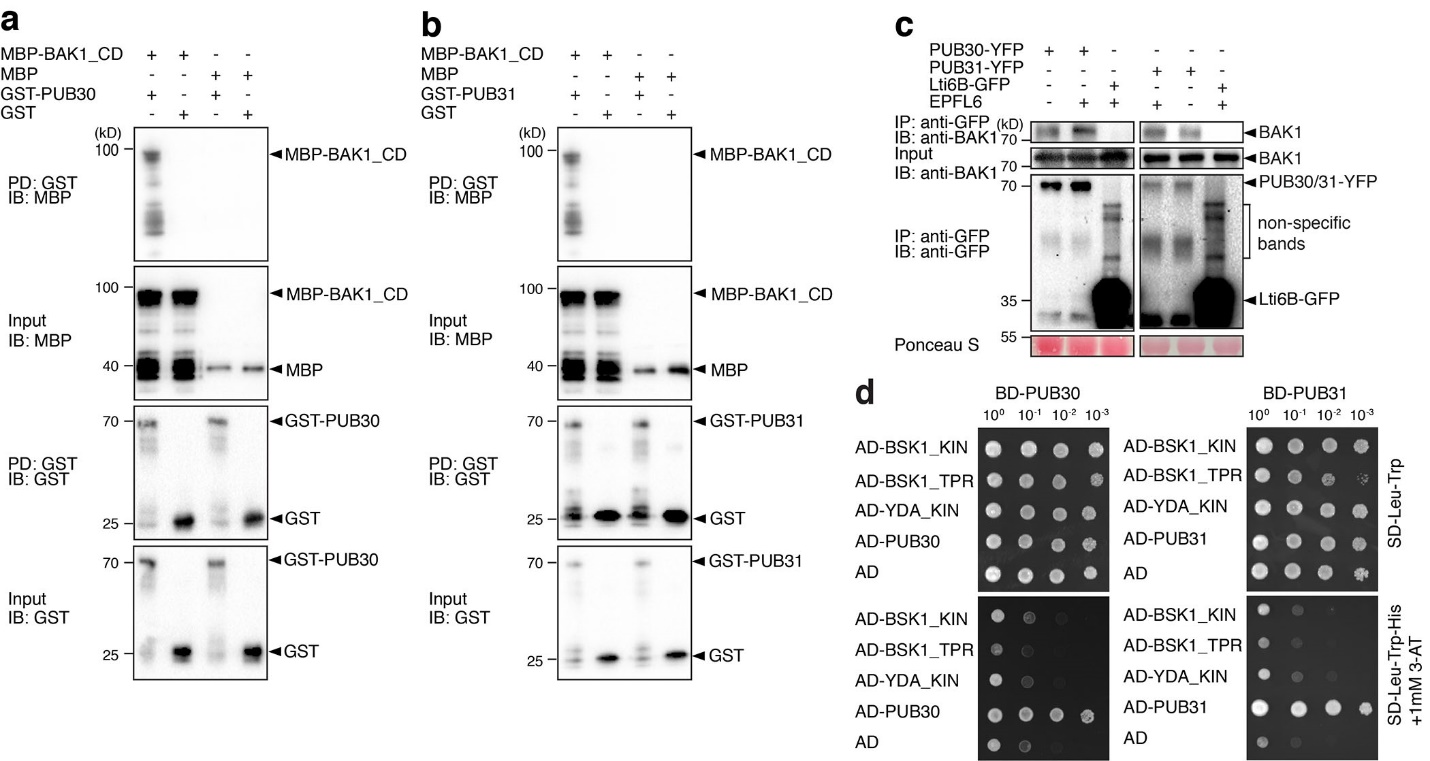
**

**Extended Data Fig. 5. PUB30/31 interact with BAK1 but not with downstream BSK1 or YODA.**

(a) PUB30 interacts with BAK1_CD *in vitro*. MBP-BAK1_CD was pulled down (PD) by GST-PUB30 immobilized on Glutathione Sepharose 4B and analyzed by immunoblotting (IB) using an anti-MBP antibody. MBP and GST were used as negative controls.

(b) PUB31 interacts with BAK1_CD *in vitro*.

(c) EPFL6 induces the association of PUB30 and PUB31 with BAK1 *in vivo*. After treatment with EPFL6, proteins from *proPUB30::PUB30-YFP*; *pub30 pub31*, *proPUB31::PUB31-YFP*; *pub30 pub31* and *Lti6B-GFP* plants were immunoprecipitated with anti-GFP beads (IP), and the immunoblots (IB) were probed with anti-BAK1 and anti-GFP antibodies, respectively. The experiment was repeated independently two times with similar results.

(d) PUB30 and PUB31 have no interaction with BSK1 or YODA (YDA) in yeast. PUB30 or PUB31 was used as bait. The TPR domain and kinase domain of BSK1, kinase domain of YDA, PUB30 or PUB31 (as a positive control), and AD alone (as a negative control) were used as prey. Yeast clones were spotted in 10-fold serial dilutions on appropriate selection media. The experiment was repeated independently three times with similar results.

**
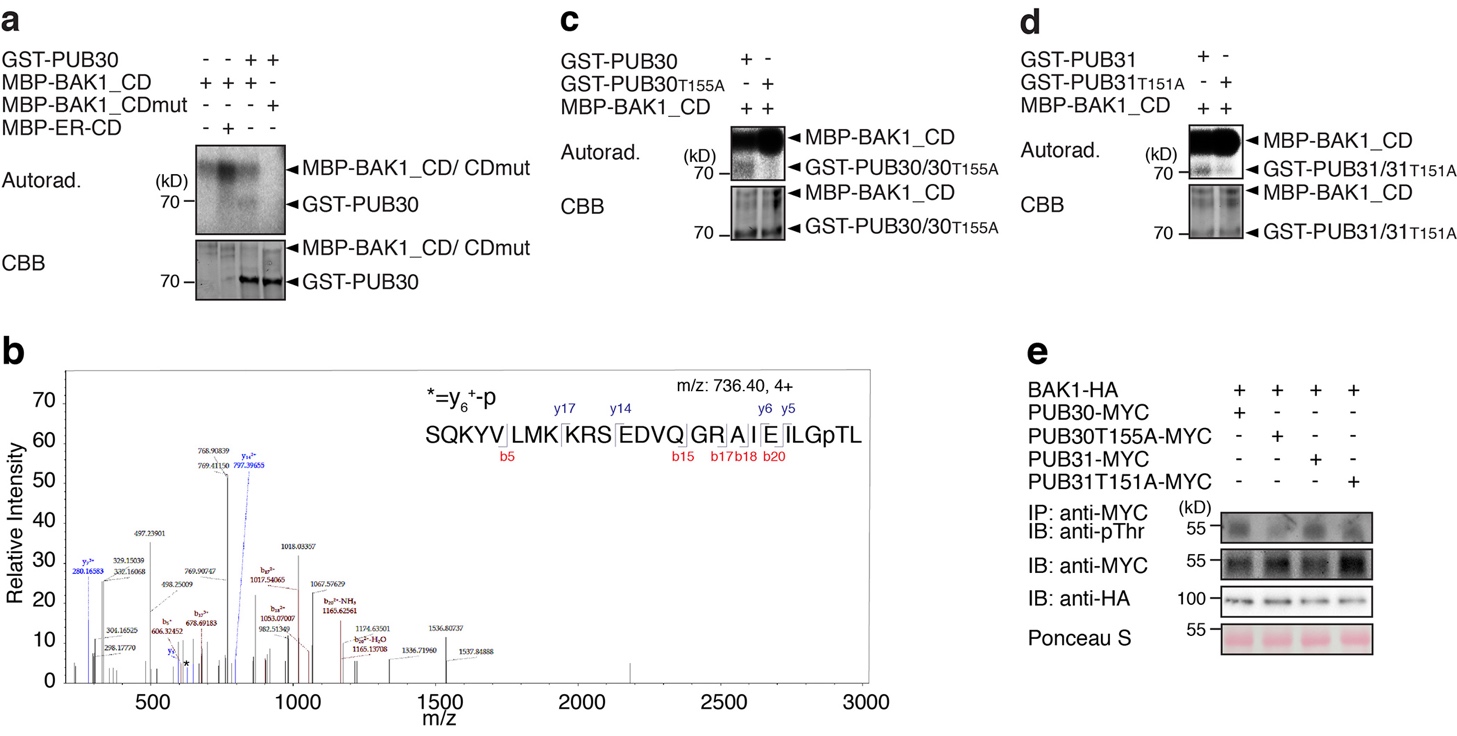
**

**Extended Data Fig. 6. BAK1 phosphorylates PUB30 and PUB31 in the linker domain.**

(a) BAK1_CD phosphorylates PUB30 *in vitro*. The phosphorylation of GST-PUB30 was carried out by using MBP-BAK1_CD as the kinase. MBP-BAK1_CD alone was used as a negative control. Combination of MBP-BAK1_CDmut and GST-PUB30 was also used as a negative control. In contrast, combination of MBP-BAK1_CD and MBP-ER_CD, but not with GST-PUB30 was used as a positive control. Autoradiography (Top) was occupied for phosphorylation detection, and CBB staining (Bottom) was performed to show the protein loading.

(b) MS/MS spectra for selected in vitro phosphorylation site of PUB30: Thr155.

(c) PUB30 T155 is required for BAK1-mediated phosphorylation. The threonine residue (PUB30_T155_) was mutated to alanine (A). GST-tagged wild-type or mutated PUB30 proteins were subjected to an *in vitro* kinase assay using MBP-BAK1_CD as the kinase.

(d) PUB31 T151 is required for BAK1-mediated phosphorylation. The threonine residue (PUB31_T151_) was mutated to alanine (A). GST-tagged wild-type or mutated PUB31 proteins were subjected to an *in vitro* kinase assay using MBP-BAK1_CD as the kinase.

(e) BAK1 mediates PUB30/31 phosphorylation *in vivo*. Arabidopsis protoplasts were co-transfected with HA-tagged BAK1 (BAK1-HA), and MYC-tagged wild-type and phosphonull versions of PUB30 or PUB31 and incubated for 8 h followed by treatment with 5 μM EPFL6 for 1 h in the presence of 2 μM MG132. After immunoprecipitation using anti-MYC beads, the phosphorylated PUB30/31 (wild-type and phosphonull versions) was probed with α-pThr antibody. The input PUB30 or PUB31 proteins and BAK1 were probed with α-MYC antibody and an α-HA antibody, respectively.

**
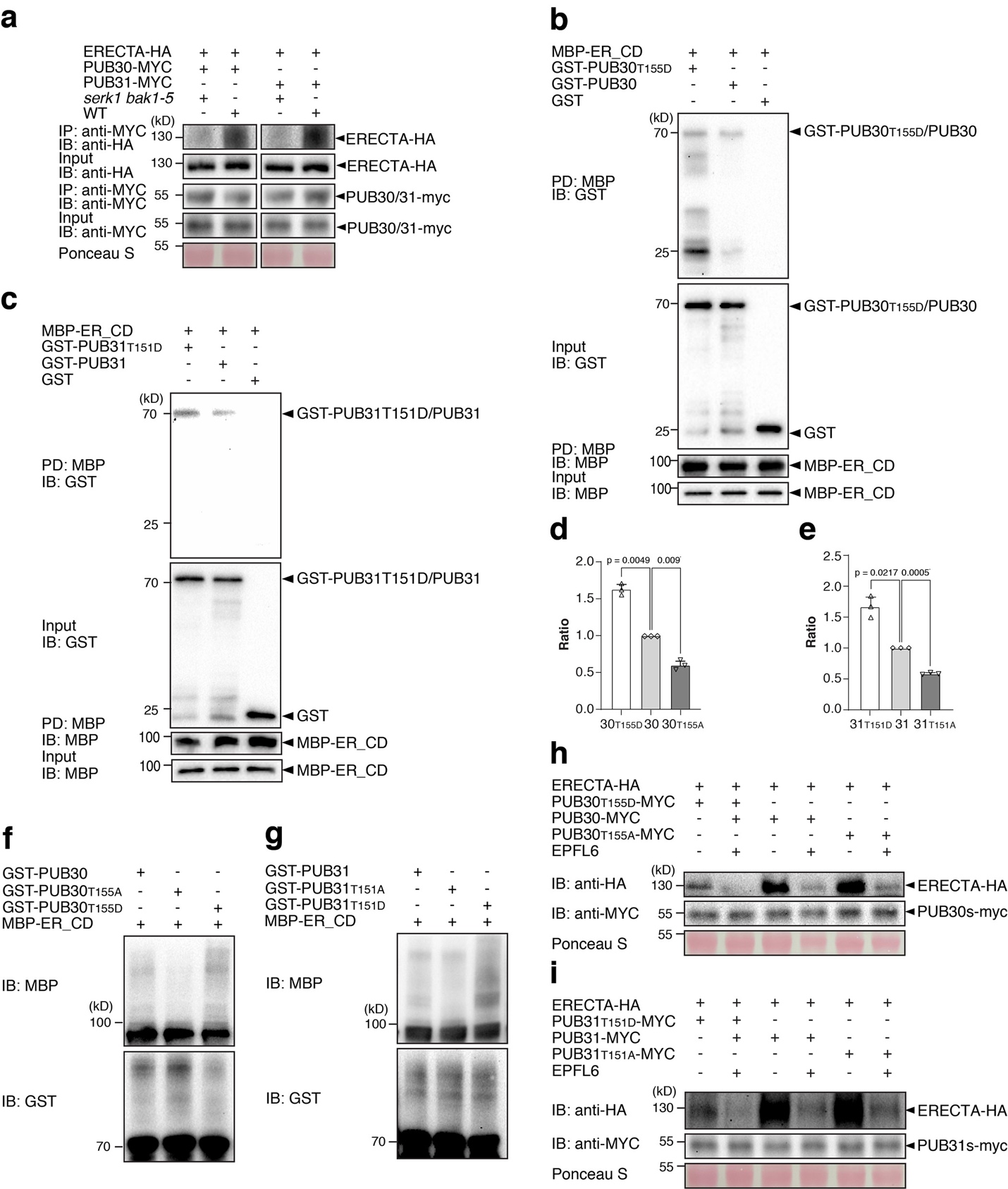
**

**Extended Data Fig. 7. Phosphorylation of PUB30 and PUB31 by BAK1 is required for their association with and ubiquitination of ERECTA.**

(a) The association of PUB30/31 with ERECTA *in vivo* in wild-type and *serk1bak1-5* background. ERECTA-HA and PUB30/PUB31-MYC were co-transfected into protoplast. After incubation for 8h, pretreatment with 2 μM MG132 for 1 h and treatment with 5 μM EPFL6 for 1 h, total proteins were immunoprecipitated with anti-MYC beads (IP), and the immunoblots (IB) were probed with anti-HA and anti-MYC antibodies, respectively.

(b) The phosphor-mutations of PUB30 affect its interaction with ER_CD *in vitro*. GST-PUB30_T155D_ or GST-PUB30 was pulled down (PD) by MBP-ER_CD immobilized on Amylose Resin and analyzed by immunoblotting (IB) using an anti-GST antibody. GST was used as a negative control.

(c) The phosphor-mutations of PUB31 affect its interaction with ER_CD *in vitro*. GST-PUB31_T151D_ or GST-PUB31 was pulled down (PD) by MBP-ER_CD and analyzed by immunoblotting (IB) using an anti-GST antibody. GST was used as a negative control.

(d, e) Quantification of *in vivo* interaction strength of ERECTA with wild-type or various phosphor-mutated PUB30/31. Error bars represent SD (n = 3). Two-tailed paired Student’s t-test was performed. p values are indicated in the graph.

(f) T155 phosphorylation of PUB30 is not prerequisite for its E3 ligase activity, but important for the ubiquitination of ER_CD. *In vitro* ubiquitination assays were performed with the indicated recombinant proteins. PUB30 auto-ubiquitination and ER_CD ubiquitination were detected with immunoblot using an anti-GST antibody and an anti-MBP antibody, respectively.

(g) T151 phosphorylation of PUB31 is not prerequisite for its E3 ligase activity, but important for the ubiquitination of ER_CD.

(h) EPFL6 treatment destabilizes ERECTA-HA in Arabidopsis protoplasts co-expressing different versions of PUB30 (PUB30_T155D_-MYC, PUB30-MYC, PUB30_T155A_-MYC). Protoplasts expressing the indicated proteins were treated with 50 μM CHX and 5 μM EPFL6 for 3 hr before total protein was examined with immunoblot. The experiment was repeated independently two times with similar results.

(i) EPFL6 treatment destabilizes ERECTA-HA in Arabidopsis protoplasts co-expressing different versions of PUB31 (PUB31_T151D_-MYC, PUB31-MYC, PUB31_T151A_-MYC). Experiments are done as described in (h). The experiment was repeated independently two times with similar results.


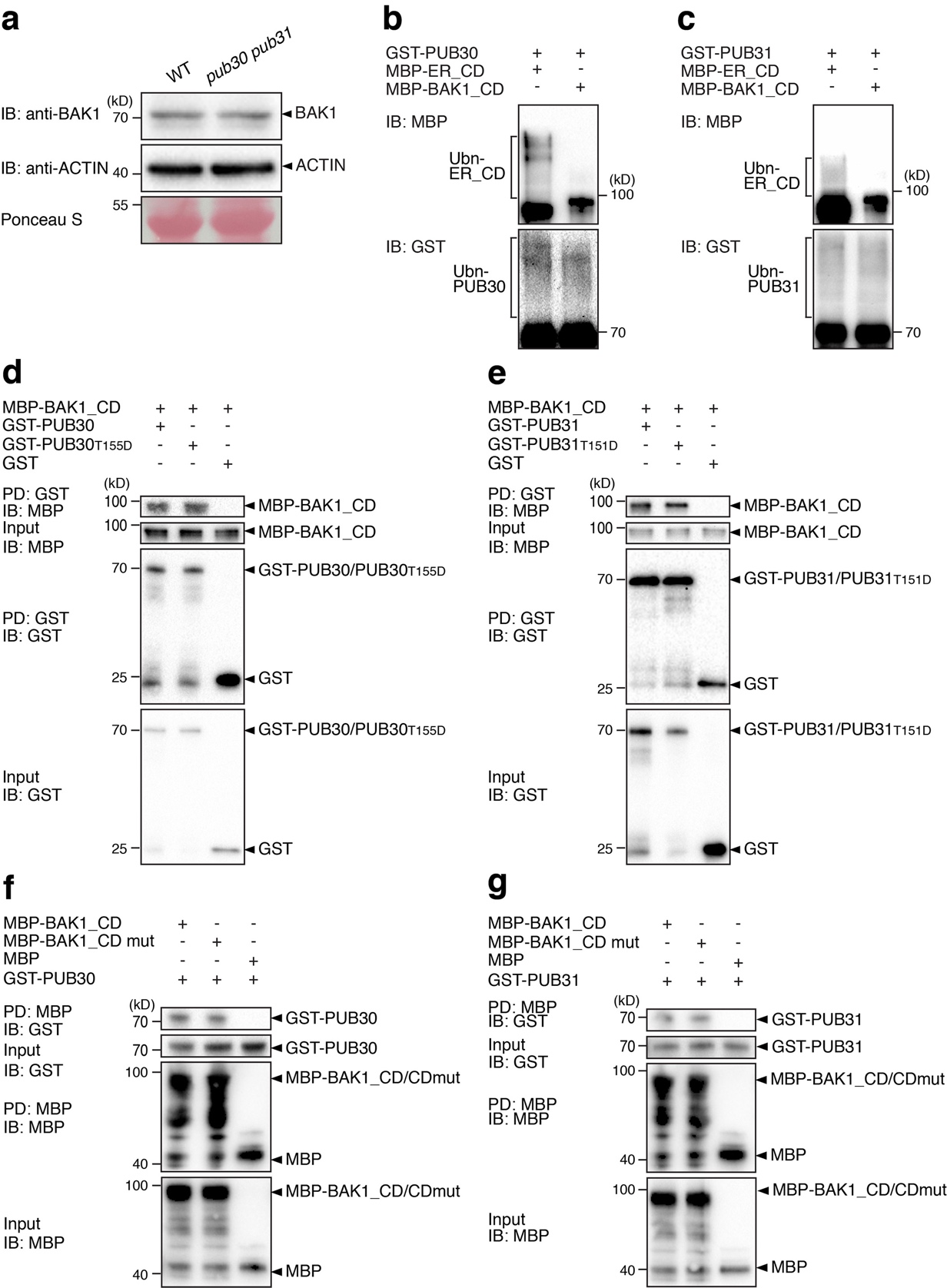


**Extended Data Fig. 8. BAK1 is not a substrate of PUB30/31.**

(a) BAK1 protein accumulation in wildtype, and *pub30 pub31*. Total proteins were isolated from 7-d-old seedlings and probed by an anti-BAK1 antibody. The protein inputs were equilibrated using α-Actin antibodies.

(b) BAK1 is not ubiquitinated by PUB30 *in vitro*. The ubiquitination assay of MBP-BAK1_CD was carried out by using GST-fused PUB30 as the E3 ligase, His-fused AtUBA1 as E1 activating enzyme, and His-fused UBC8 as E2 conjugating enzyme. MBP-ER_CD was used as a positive control.

(c) BAK1 is not ubiquitinated by PUB31 *in vitro*.

(d) The phosphorylation of PUB30 by BAK1 does not affect its interaction with BAK1_CD *in vitro*. MBP-BAK1_CD was pulled down (PD) by GST-PUB30 or GST-PUB30_T155D_ immobilized on Glutathione Sepharose 4B and analyzed by immunoblotting (IB) using an anti-MBP antibody. GST was used as a negative control.

(e) The phosphorylation of PUB31 by BAK1 does not affect its interaction with BAK1_CD *in vitro*.

(f) The kinase activity of BAK1 does not affect its interaction with PUB30 *in vitro*. GST-PUB30 was pulled down (PD) by MBP-BAK1_CD or MBP-BAK1_CDmut immobilized on Amylose Resin and analyzed by immunoblotting (IB) using an anti-GST antibody. MBP was used as a negative control.

(g) The kinase activity of BAK1 does not affect its interaction with PUB31 *in vitro*.

**
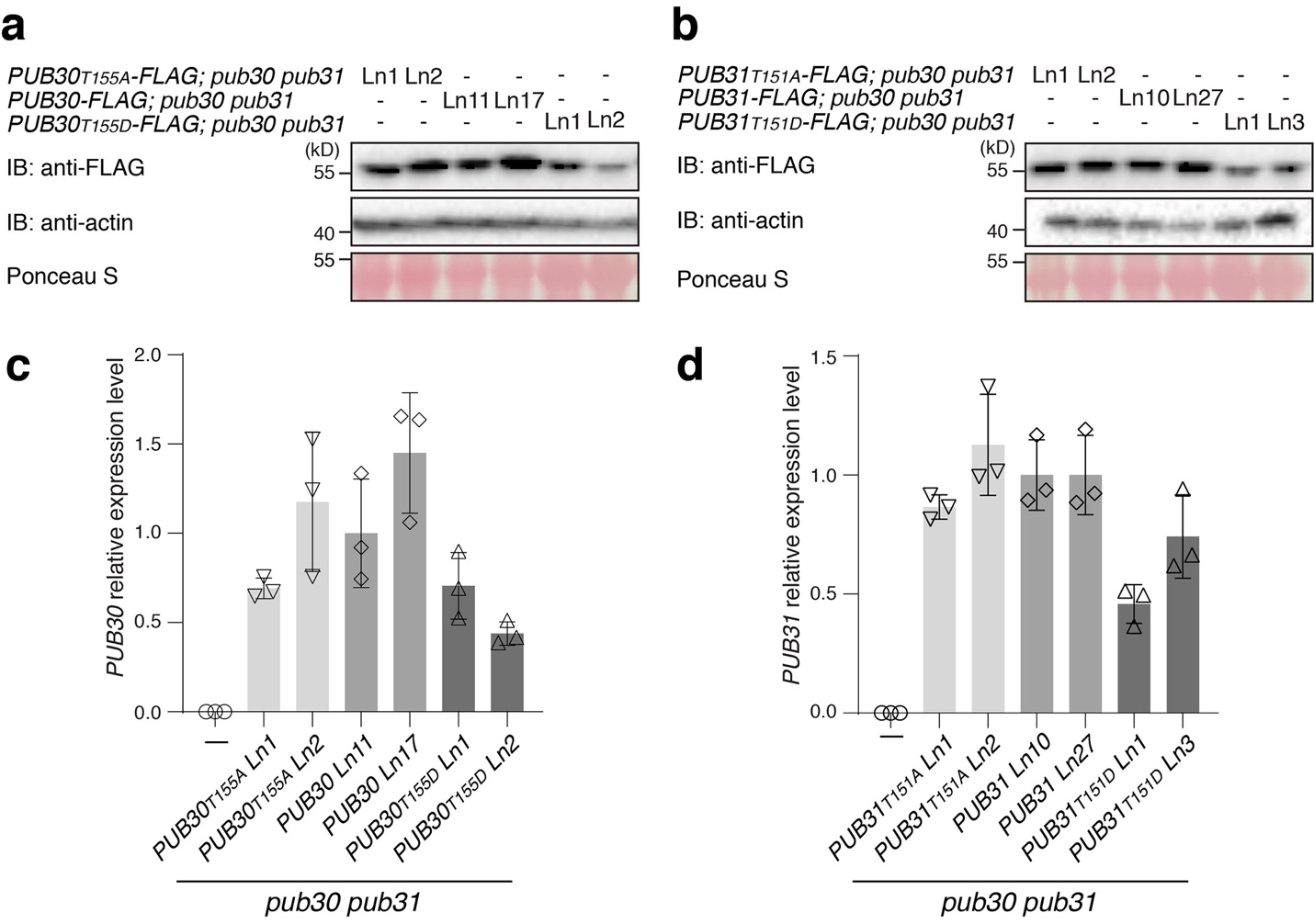
**

**Extended Data Fig. 9. Protein and transcript levels of phosphonull and phosphomimetics PUB30/31 in Arabidopsis seedlings.**

(a) The protein levels of wild-type or various phosphosite mutated PUB30 in the Arabidopsis complementation lines. Total proteins were isolated from 7-d-old seedlings and probed by an α-FLAG antibody. An α-Actin antibody was used as a native control.

(b) The protein levels of wild-type or various phosphosite mutated PUB31 in the Arabidopsis complementation lines. Total proteins were isolated from 7-d-old seedlings and probed by an α-FLAG antibody. An α-Actin antibody was used as a native control.

(c) The transcript levels of PUB30 by qRT-PCR analysis in *pub30 pub31* and wild-type or various phosphosite mutated PUB30 complementation lines. Error bars represent SD (n = 3).

(d) The transcript levels of PUB30 by qRT-PCR analysis of *PUB31* in *pub30 pub31* and wild-type or various phosphosite mutated PUB30 complementation lines. Error bars represent SD (n = 3).

**
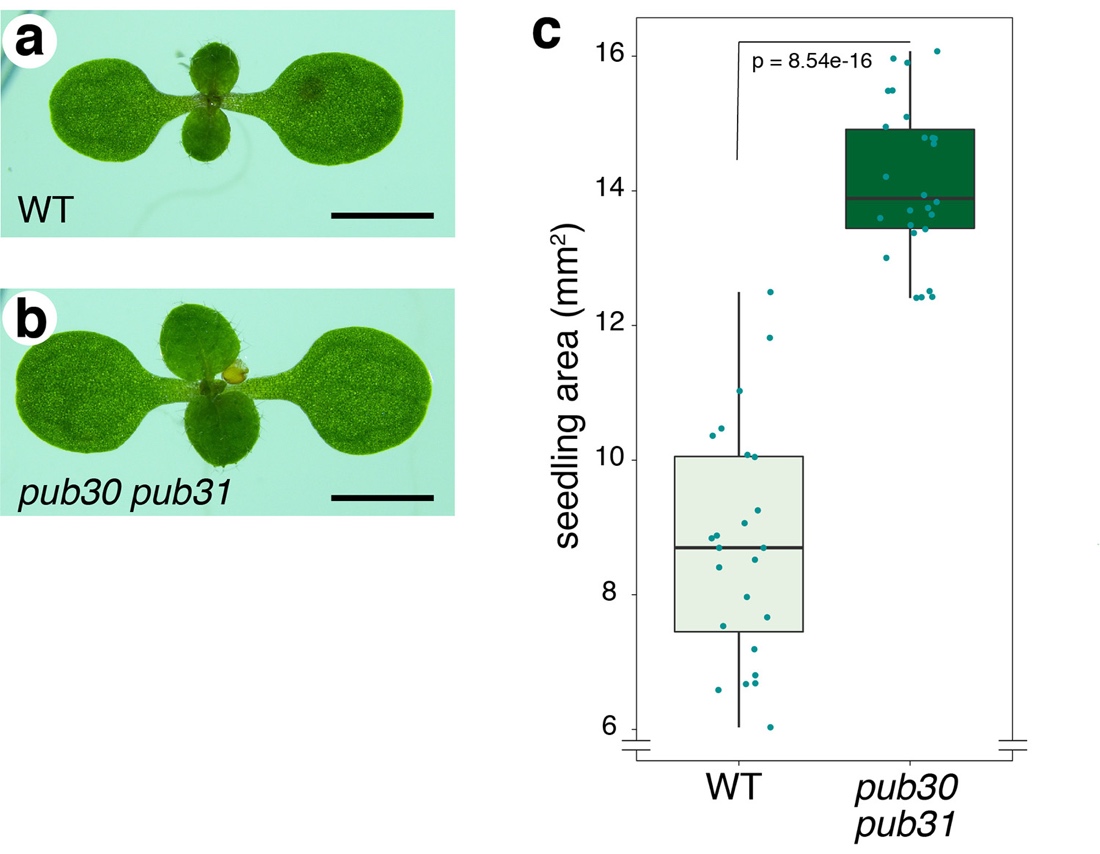
**

**Extended Data Fig. 10. The *pub30 pub31* double mutant seedlings exhibit hyperplasia.**

(a, b) Representative 7-day-old seedlings of wild type (WT, a) and the *pub30 pub31* double mutant (b). Images are taken under the same magnification. Scale bars, 2 mm

(c) Quantitative analysis of seedling shoot area from wild-type (WT) and the *pub30 pub31* double mutant. Welch's two-tailed unpaired t-test was performed (p = 8.54e-16). n= 24 (WT), n=26 (*pub30 pub31*)
