# Supplementary material for: Direct attenuation of Arabidopsis ERECTA signalling by a pair of U-box E3 ligases
Source: Nat Plants. 2022 Dec 20;9(1):112–27. doi: 10.1038/s41477-022-01303-x (PMC9873567; doi:10.1038/s41477-022-01303-x)
Supplement: Supplementary file 2 — Reporting Summary [file 41477_2022_1303_MOESM2_ESM.pdf]

nature portfolio

Corresponding author(s): TORII, Keiko U.

Last updated by author(s): Oct 24, 2022

Reporting Summary

Nature Portfolio wishes to improve the reproducibility of the work that we publish. This form provides structure for consistency and transparency in reporting. For further information on Nature Portfolio policies, see our Editorial Policies and the Editorial Policy Checklist.

Statistics

For all statistical analyses, confirm that the following items are present in the figure legend, table legend, main text, or Methods section.

n/a	Confirmed	
☐	☒	The exact sample size (n) for each experimental group/condition, given as a discrete number and unit of measurement
☐	☒	A statement on whether measurements were taken from distinct samples or whether the same sample was measured repeatedly
☐	☒	The statistical test(s) used AND whether they are one- or two-sided<br>*Only common tests should be described solely by name; describe more complex techniques in the Methods section.*
☐	☒	A description of all covariates tested
☐	☒	A description of any assumptions or corrections, such as tests of normality and adjustment for multiple comparisons
☐	☒	A full description of the statistical parameters including central tendency (e.g. means) or other basic estimates (e.g. regression coefficient) AND variation (e.g. standard deviation) or associated estimates of uncertainty (e.g. confidence intervals)
☐	☒	For null hypothesis testing, the test statistic (e.g. F, t, r) with confidence intervals, effect sizes, degrees of freedom and P value noted<br>*Give P values as exact values whenever suitable.*
☒	☐	For Bayesian analysis, information on the choice of priors and Markov chain Monte Carlo settings
☒	☐	For hierarchical and complex designs, identification of the appropriate level for tests and full reporting of outcomes
☒	☐	Estimates of effect sizes (e.g. Cohen's d, Pearson's r), indicating how they were calculated

Our web collection on statistics for biologists contains articles on many of the points above.

Software and code

Policy information about availability of computer code

Data collection	Image J (version 1.53c), Image Lab (Bio-Rad, version 6.0.1) were used in data collection. The data of biolayer interferometry (BLI) were collected using the Octet data acquisition software 10.0.
Data analysis	Statistical analysis of phenotypes experiments was performed using R ver. 4.1.0 operated under R-Studio ver. 1.4.1717 (https://www.rstudio.com), and graphs were generated using R ggplot2 package. The data of biolayer interferometry (BLI) were analyzed using the ForteBio data analysis software (Octet BLI Analysis 10.0). Statistical analysis of biochemistry experiments was performed using GraphPad Prism 3.1. The MS/MS spectra were analyzed with SEQUEST (ThermoFisher Scientific). Images were cropped using Adobe Photoshop 2022.

For manuscripts utilizing custom algorithms or software that are central to the research but not yet described in published literature, software must be made available to editors and reviewers. We strongly encourage code deposition in a community repository (e.g. GitHub). See the Nature Portfolio guidelines for submitting code & software for further information.

Data

Policy information about availability of data

All manuscripts must include a data availability statement. This statement should provide the following information, where applicable:

- Accession codes, unique identifiers, or web links for publicly available datasets
- A description of any restrictions on data availability
- For clinical datasets or third party data, please ensure that the statement adheres to our policy

All generated and analyzed data from this study are included in the main figures, Extended Data figures and supplementary information. Source data are provided with this paper.

Field-specific reporting

Please select the one below that is the best fit for your research. If you are not sure, read the appropriate sections before making your selection.

☒ Life sciences ☐ Behavioural & social sciences ☐ Ecological, evolutionary & environmental sciences

For a reference copy of the document with all sections, see nature.com/documents/nr-reporting-summary-flat.pdf

Life sciences study design

All studies must disclose on these points even when the disclosure is negative.

Sample size	Sample size was determined based on similar studies in this field. For stomatal index analysis, the sample size was at least 7. For pedicel length analysis, the sample size was at least 8.
Data exclusions	No data were excluded.
Replication	To ensure robust reproducibility: All Octet data presented in this manuscript were repeated two times. All Y2H, immunoblot assays were repeated at least two times. To derive the statistics, three biological independent replicates of related experiments were performed. All confocal images presented were imaged at least thrice for a single data point. For transgenic lines: at least two individual lines for each transgenic plant were analyzed before carrying out further experiments with the lines. For quantification of stomata index of transgenic and mutant lines used in the manuscript, at least seven independent replicates were used for each genotype tested.
Randomization	Plants for all phenotypic characterizations were randomly chosen among each genotype population. Positions of plants in pots and petri dishes were exchanged every day to minimize the effect of environmental variability in the growth room.
Blinding	No blinding was performed in this study. This is because blinding requires mixing seeds of different genotypes (mutants and transgenic lines), which would result in a risk of mislabeling.

Reporting for specific materials, systems and methods

We require information from authors about some types of materials, experimental systems and methods used in many studies. Here, indicate whether each material, system or method listed is relevant to your study. If you are not sure if a list item applies to your research, read the appropriate section before selecting a response.

Materials & experimental systems

n/a	Involved in the study	
☐	☒	Antibodies
☒	☐	Eukaryotic cell lines
☒	☐	Palaeontology and archaeology
☒	☐	Animals and other organisms
☒	☐	Human research participants
☒	☐	Clinical data
☒	☐	Dual use research of concern

Methods

n/a	Involved in the study	
☒	☐	ChIP-seq
☒	☐	Flow cytometry
☒	☐	MRI-based neuroimaging

Antibodies

Antibodies used

1, Mouse monoclonal anti-HA (Clone number: HA.C5), Abcam, Cat# ab18181 RRID: AB_444303, Lot# GR3230856-1; GR3380510-2; GR3410448-2
2, Mouse monoclonal anti-Myc, Abcam, Cat# ab32 RRID: AB_303599, Lot# GR206680-8
3, Rabbit polyclonal anti-Myc, Abcam, Cat# ab9106 RRID: AB_307014, Lot# GR130480-27
4, Mouse monoclonal anti-FLAG, Sigma, Cat# F3165 RRID: AB_259529, Lot# SLCG2330
5, Rabbit monoclonal anti-FLAG, Abcam, Cat# ab205606, Lot# GR3293857-1, GR3293857-19
6, Rabbit polyclonal anti-GFP, Abcam, Cat# ab290 also ENCAB615WUN RRID: AB_303395, Lot# GR278073-1
7, Mouse monoclonal anti-GFP, Thermo Scientific Fisher, Cat# 33-2600 RRID: AB_2533111, Lot# WC324562; WE325933
8, Mouse monoclonal Anti-MBP, NEB, Cat# E8032S Lot# 0101705
9, Mouse monoclonal Anti-GST, Genscript, Cat# A00865-200
10, Mouse Anti-Actin antibody [mAbGEa], Abcam, Cat# ab230169, Lot# GR3244091-1
11, Rabbit polyclonal anti-BAK1, Product # AS12 1858, Agrisera, Lot# 1904
12, Anti-Ubiquitin Antibody, clone P4D1-A11, Sigma, Cat# 05-944, Lot# 2895882, dilution of 1:2500
13, Anti-Mouse HRP conjugated secondary antibody, GE Healthcare, Cat#NA931VS Lot# 16889299
14, Anti-Mouse HRP conjugated secondary antibody, Abcam, Cat# ab205719 Lot# GR3279214-1; GR3405228-1
15, Anti-Rabbit HRP conjugated secondary antibody, Sigma, Cat# A6154 Lot# SLBV9141
16, Mouse monoclonal anti-Phospho-Threonine (42H4), Cat# 9386, Lot# 12

Validation

Validation statements, relevant citations of commercial primary antibodies are available from manufacturers:
1, https://www.abcam.com/ha-tag-antibody-hac5-ab18181.html
2, https://www.abcam.com/myc-tag-antibody-9e10-ab32.html
3, https://www.abcam.com/myc-tag-antibody-ab9106.html
4, https://www.sigmaaldrich.com/US/en/product/sigma/f3165
5, https://www.abcam.com/ddddk-tag-binds-to-flag-tag-sequence-antibody-epr20018-251-ab205606.html
6, https://www.abcam.com/gfp-antibody-ab290.html
7, https://www.thermofisher.com/antibody/product/GFP-Antibody-clone-C163-Monoclonal/33-2600
8, https://www.neb.com/products/e8032-anti-mbp-monoclonal-antibody#Product%20Information
9, https://www.genscript.com/antibody/A00865-THE_GST_Antibody_mAb_Mouse.html
10, https://www.abcam.com/actin-antibody-mabgea-ab230169.html
11, https://www.agrisera.com/en/artiklar/bak1-bri1-associated-receptor-kinase.html
12, https://www.sigmaaldrich.com/US/en/product/mm/05944?
gclid=Cj0KCQiAkNiMBhCxARIsAIDDKNXuvhZscV95HXHtSCsSinEICH_0R5K9bCKWe4nZZCTEsd7o6pz3pWgaAt2dEALw_wcB
13, https://www.sigmaaldrich.com/catalog/product/sigma/gena93101ml?lang=en&region=US
14, https://www.abcam.com/goat-mouse-igg-hl-hrp-ab205719.html
15, https://www.sigmaaldrich.com/US/en/product/sigma/a6154?context=product
16, https://www.cellsignal.com/products/primary-antibodies/phospho-threonine-42h4-mouse-mab/9386
