# Supplementary material for: Direct attenuation of Arabidopsis ERECTA signalling by a pair of U-box E3 ligases
Source: Nat Plants. 2022 Dec 20;9(1):112–27. doi: 10.1038/s41477-022-01303-x (PMC9873567; doi:10.1038/s41477-022-01303-x)
Supplement: Supplementary file 4 — Extended dataset P values for one-way ANOVA–Tukey’s HSD test. [file 41477_2022_1303_MOESM4_ESM.pdf]

#Extended Dataset p-values for One-way ANOVA-Tukey's HSD Test

#Fig. 1c, Pedicel Length

$genotype

	diff	lwr	upr	p adj
pub30-er105	6.1450	5.2591501	7.030849854	0.0000000
pub30pub31-er105	16.1170	15.2311501	17.002849854	0.0000000
pub30pub31er105-er105	0.2855	-0.6003499	1.171349854	0.9368058
pub31-er105	5.4495	4.5636501	6.335349854	0.0000000
WT-er105	5.2680	4.3821501	6.153849854	0.0000000
pub30pub31-pub30	9.9720	9.0861501	10.857849854	0.0000000
pub30pub31er105-pub30	-5.8595	-6.7453499	-4.973650146	0.0000000
pub31-pub30	-0.6955	-1.5813499	0.190349854	0.2125546
WT-pub30	-0.8770	-1.7628499	0.008849854	0.0539795
pub30pub31er105-pub30pub31	-15.8315	-16.7173499	-14.945650146	0.0000000
pub31-pub30pub31	-10.6675	-11.5533499	-9.781650146	0.0000000
WT-pub30pub31	-10.8490	-11.7348499	-9.963150146	0.0000000
pub31-pub30pub31er105	5.1640	4.2781501	6.049849854	0.0000000
WT-pub30pub31er105	4.9825	4.0966501	5.868349854	0.0000000
WT-pub31	-0.1815	-1.0673499	0.704349854	0.9912615

#Fig. 1g, Stomatal Index

$genotype

	diff	lwr	upr	p adj
pub30pub31-pub30	-7.0634924	-11.6940092	-2.432976	0.0014561
pub31-pub30	0.2354632	-4.3950536	4.865980	0.9990212
WT-pub30	4.1883996	-0.4421172	8.818916	0.0870800
pub31-pub30pub31	7.2989556	2.6684389	11.929472	0.0010078
WT-pub30pub31	11.2518920	6.6213753	15.882409	0.0000020
WT-pub31	3.9529364	-0.6775804	8.583453	0.1150998

#Fig. 1i, Stomata + Meristemoid Index

$genotype

	diff	lwr	upr	p adj
pub30pub31-er105	-13.1269794	-17.488857	-8.765102	0.0000000
pub30pub31er105-er105	-1.4396448	-5.801522	2.922233	0.8042911
WT-er105	-1.8750874	-6.236965	2.486790	0.6479836
pub30pub31er105-pub30pub31	11.6873346	7.325457	16.049212	0.0000003
WT-pub30pub31	11.2518920	6.890015	15.613770	0.0000007
WT-pub30pub31er105	-0.4354426	-4.797320	3.926435	0.9927599

#Extended Fig.1e Pedicel lengths of complementation lines

$genotype

	diff	lwr	upr	p adj
PUB30COM-17-PUB30COM-11	0.78475753	-1.592697	3.162212	0.9283330
pub30pub31-PUB30COM-11	13.21468474	10.837230	15.592139	0.0000000
PUB31COM-10-PUB30COM-11	-0.40644726	-2.783902	1.971007	0.9960985

PUB31COM-27-PUB30COM-11	4.80273328	2.425279	7.180188	0.0000011
WT-PUB30COM-11	-0.46749324	-2.844948	1.909961	0.9924997
pub30pub31-PUB30COM-17	12.42992721	10.052473	14.807382	0.0000000
PUB31COM-10-PUB30COM-17	-1.19120479	-3.568659	1.186250	0.6894929
PUB31COM-27-PUB30COM-17	4.01797575	1.640521	6.395430	0.0000591
WT-PUB30COM-17	-1.25225077	-3.629705	1.125204	0.6421288
PUB31COM-10-pub30pub31	-13.62113200	-15.998586	-11.243678	0.0000000
PUB31COM-27-pub30pub31	-8.41195146	-10.789406	-6.034497	0.0000000
WT-pub30pub31	-13.68217798	-16.059632	-11.304724	0.0000000
PUB31COM-27-PUB31COM-10	5.20918054	2.831726	7.586635	0.0000001
WT-PUB31COM-10	-0.06104598	-2.438500	2.316408	0.9999997
WT-PUB31COM-27	-5.27022652	-7.647681	-2.892772	0.0000001

#Extended Fig.1g Stomatal Index of complementation lines

$genotype

	diff	lwr	upr	p adj
PUB30COM-17-PUB30COM-11	0.2043481	-2.8277825	3.236479	0.9999542
pub30pub31-PUB30COM-11	-7.4803420	-10.5124726	-4.448211	0.0000000
PUB31COM-10-PUB30COM-11	-2.3260616	-5.3581922	0.706069	0.2257018
PUB31COM-27-PUB30COM-11	-0.4104618	-3.4425925	2.621669	0.9986074
WT-PUB30COM-11	0.3246870	-2.7074437	3.356818	0.9995529
pub30pub31-PUB30COM-17	-7.6846901	-10.7168207	-4.652559	0.0000000
PUB31COM-10-PUB30COM-17	-2.5304097	-5.5625403	0.501721	0.1528047
PUB31COM-27-PUB30COM-17	-0.6148099	-3.6469405	2.417321	0.9906661
WT-PUB30COM-17	0.1203389	-2.9117917	3.152470	0.9999967
PUB31COM-10-pub30pub31	5.1542804	2.1221498	8.186411	0.0000838
PUB31COM-27-pub30pub31	7.0698801	4.0377495	10.102011	0.0000001
WT-pub30pub31	7.8050289	4.7728983	10.837160	0.0000000
PUB31COM-27-PUB31COM-10	1.9155997	-1.1165309	4.947730	0.4334050
WT-PUB31COM-10	2.6507485	-0.3813821	5.682879	0.1192167
WT-PUB31COM-27	0.7351488	-2.2969818	3.767279	0.9791411

#Extended Fig.3h Pedicel length of PUB inactive E3 ligase mutations

$genotype

	diff	lwr	upr	p adj
PUB30W97Acom-pub30pub31	-0.118125	-0.27671014	0.04046014	0.2000235
PUB31W93Acom-pub30pub31	-0.053375	-0.21196014	0.10521014	0.7949745
WT-pub30pub31	-0.859000	-1.01758514	-0.70041486	0.0000000
PUB31W93Acom-PUB30W97Acom	0.064750	-0.09383514	0.22333514	0.6836203
WT-PUB30W97Acom	-0.740875	-0.89946014	-0.58228986	0.0000000
WT-PUB31W93Acom	-0.805625	-0.96421014	-0.64703986	0.0000000

#Extended Fig.3i Stomatal Index of PUB inactive E3 ligase mutations

$genotype

	diff	lwr	upr	p adj
31WAcom-30WAcom	1.054925	-3.063300	5.173151	0.8934655
pub30pub31-30WAcom	2.976321	-1.141904	7.094547	0.2182122
WT-30WAcom	8.483532	4.365306	12.601757	0.0000419
pub30pub31-31WAcom	1.921396	-2.196829	6.039622	0.5796845
WT-31WAcom	7.428607	3.310381	11.546832	0.0002433
WT-pub30pub31	5.507210	1.388985	9.625436	0.0059159

#Extended Fig.5f Pedicel length of PUB phosphosite mutations

$genotype

	diff	lwr	upr	p adj
T151Acom-pub30pub31	-1.94625	-4.240691	0.348191	0.1379326
T151Dcom-pub30pub31	-8.36000	-10.654441	-6.065559	0.0000000
T155Acom-pub30pub31	-1.59000	-3.884441	0.704441	0.3229135
T155Dcom-pub30pub31	-9.92000	-12.214441	-7.625559	0.0000000
WT-pub30pub31	-8.79500	-11.089441	-6.500559	0.0000000
T151Dcom-T151Acom	-6.41375	-8.708191	-4.119309	0.0000000
T155Acom-T151Acom	0.35625	-1.938191	2.650691	0.9971375
T155Dcom-T151Acom	-7.97375	-10.268191	-5.679309	0.0000000
WT-T151Acom	-6.84875	-9.143191	-4.554309	0.0000000
T155Acom-T151Dcom	6.77000	4.475559	9.064441	0.0000000
T155Dcom-T151Dcom	-1.56000	-3.854441	0.734441	0.3434349
WT-T151Dcom	-0.43500	-2.729441	1.859441	0.9927097
T155Dcom-T155Acom	-8.33000	-10.624441	-6.035559	0.0000000
WT-T155Acom	-7.20500	-9.499441	-4.910559	0.0000000
WT-T155Dcom	1.12500	-1.169441	3.419441	0.6884943

#Extended Fig.3g Stomatal Index of PUB phosphosite mutations

$genotype

	diff	lwr	upr	p adj
PUB30TI55Acom-pub30pub31	1.5663023	-1.711486	4.8440905	0.7197942
PUB30TI55Dcom-pub30pub31	11.5050768	8.227289	14.7828650	0.0000000
PUB31T151Acom-pub30pub31	0.6440196	-2.633769	3.9218078	0.9919227
PUB31T151Dcom-pub30pub31	10.1087599	6.830972	13.3865481	0.0000000
WT-pub30pub31	7.8050289	4.527241	11.0828172	0.0000001
PUB30TI55Dcom-PUB30TI55Acom	9.9387745	6.660986	13.2165628	0.0000000
PUB31T151Acom-PUB30TI55Acom	-0.9222827	-4.200071	2.3555055	0.9603390
PUB31T151Dcom-PUB30TI55Acom	8.5424576	5.264669	11.8202459	0.0000000
WT-PUB30TI55Acom	6.2387266	2.960938	9.5165149	0.0000098
PUB31T151Acom-PUB30TI55Dcom	-10.8610572	-14.138845	-7.5832690	0.0000000
PUB31T151Dcom-PUB30TI55Dcom	-1.3963169	-4.674105	1.8814713	0.8057015
WT-PUB30TI55Dcom	-3.7000479	-6.977836	-0.4222597	0.0183274
PUB31T151Dcom-PUB31T151Acom	9.4647403	6.186952	12.7425285	0.0000000
WT-PUB31T151Acom	7.1610093	3.883221	10.4387976	0.0000005
WT-PUB31T151Dcom	-2.3037310	-5.581519	0.9740572	0.3148091
