# Supplementary material for: Direct attenuation of Arabidopsis ERECTA signalling by a pair of U-box E3 ligases
Source: Nat Plants. 2022 Dec 20;9(1):112–27. doi: 10.1038/s41477-022-01303-x (PMC9873567; doi:10.1038/s41477-022-01303-x)

Original Uncropped Scanned Images of blots and gels

Fig. 2d, e

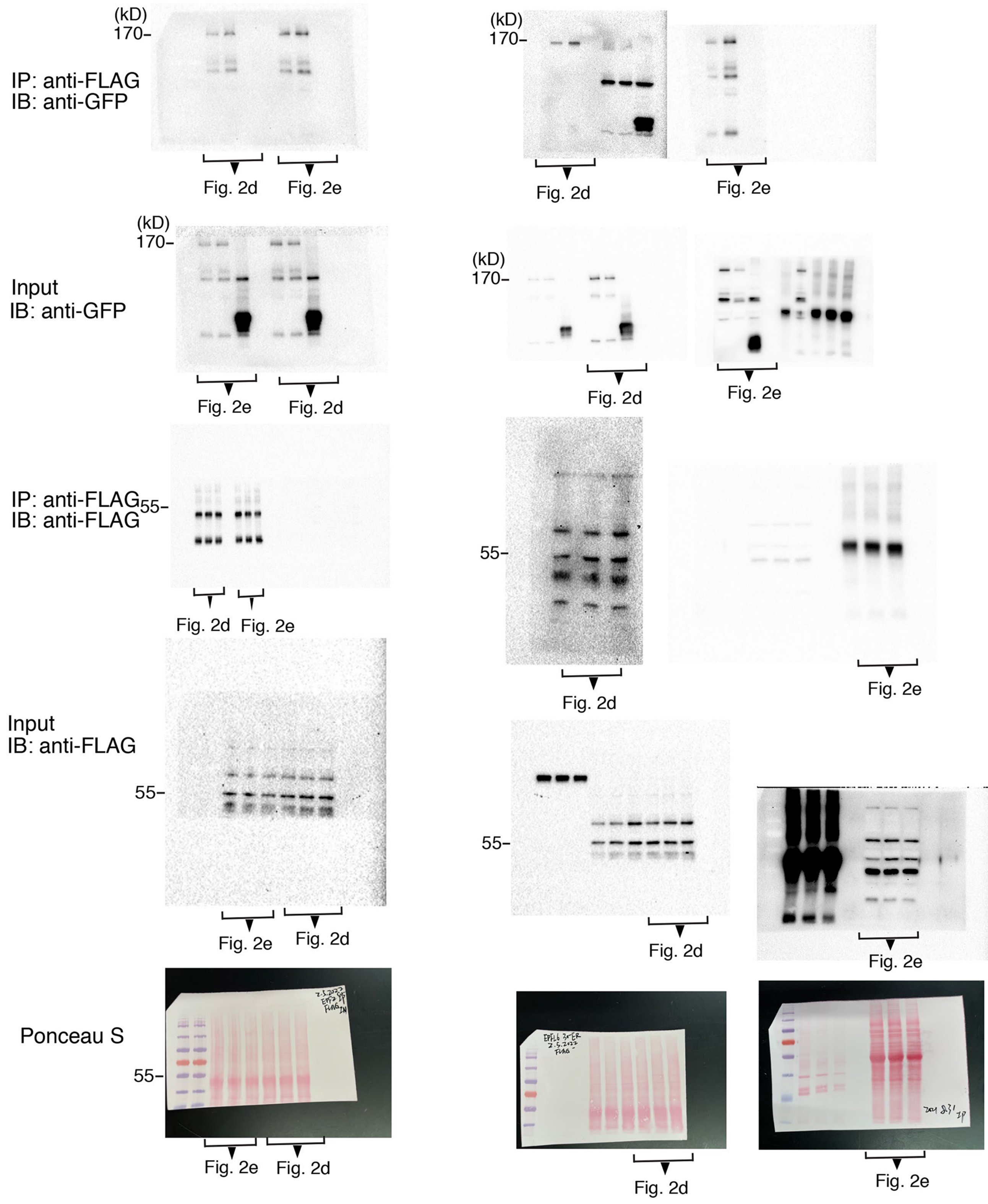

The above:
EPF2 treatment for PUB30-ERECTA (Fig.2d)
and PUB31-ERECTA (Fig.2e)

The above:
EPFL6 treatment for PUB30-ERECTA (Fig.2d)
and PUB31-ERECTA (Fig.2e)

Original Uncropped Scanned Images of blots and gels

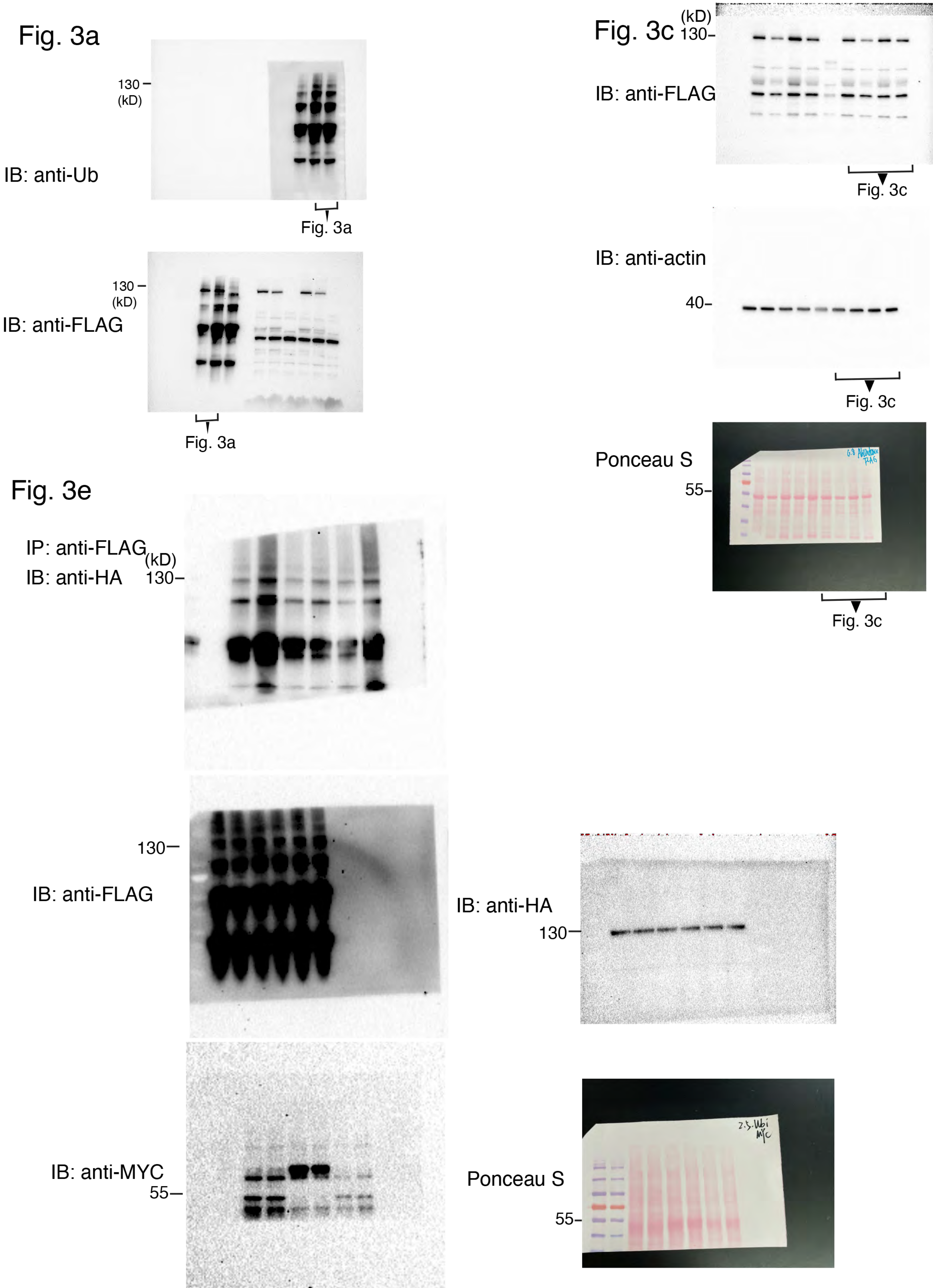

Original Uncropped Scanned Images of blots and gels

Fig. 4d

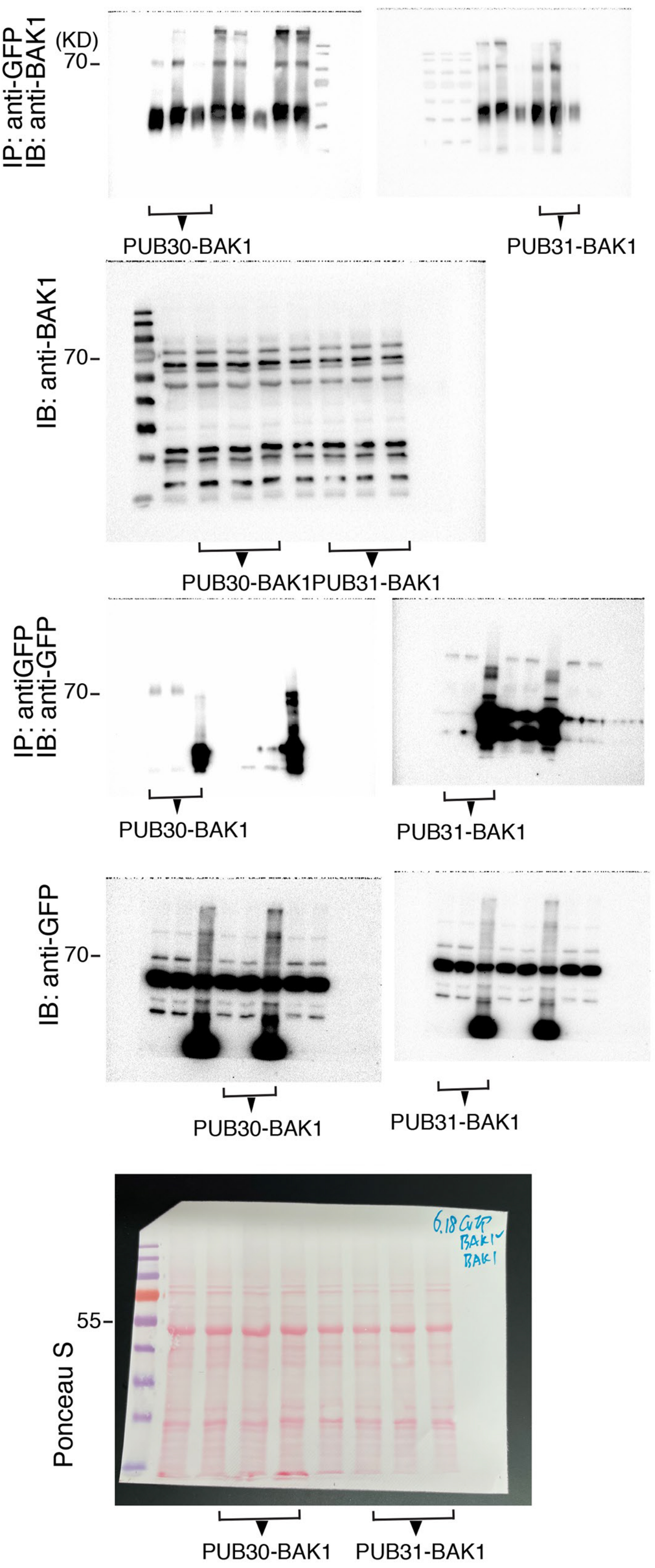

Fig. 4e, f

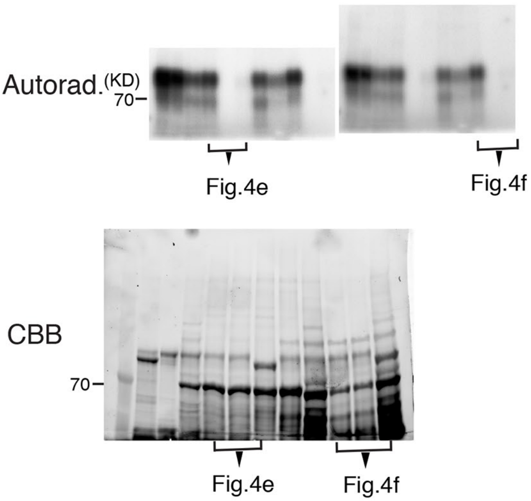

Original Uncropped Scanned Images of blots and gels

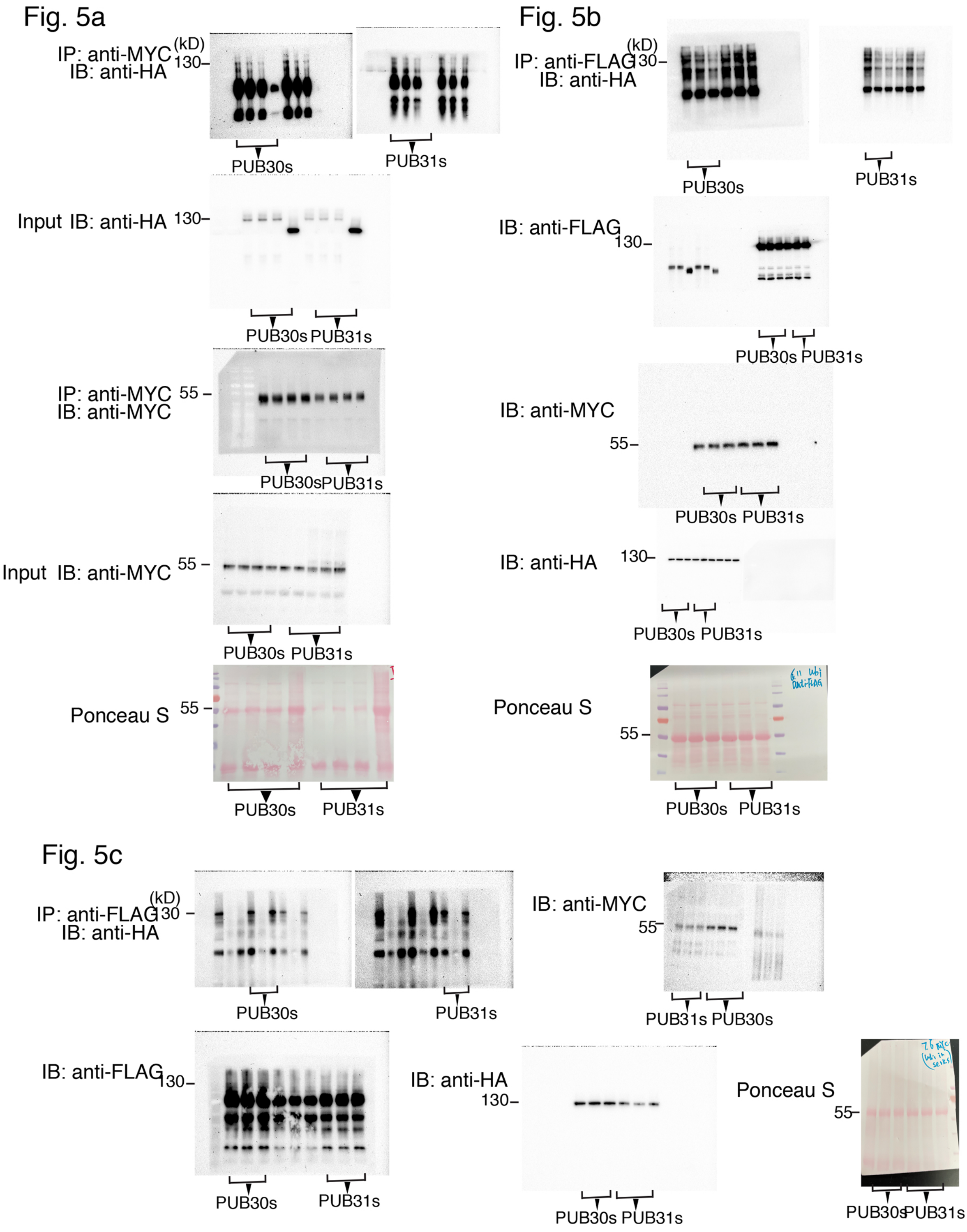

Original Uncropped Scanned Images of blots and gels

Extended Fig. 2c, d

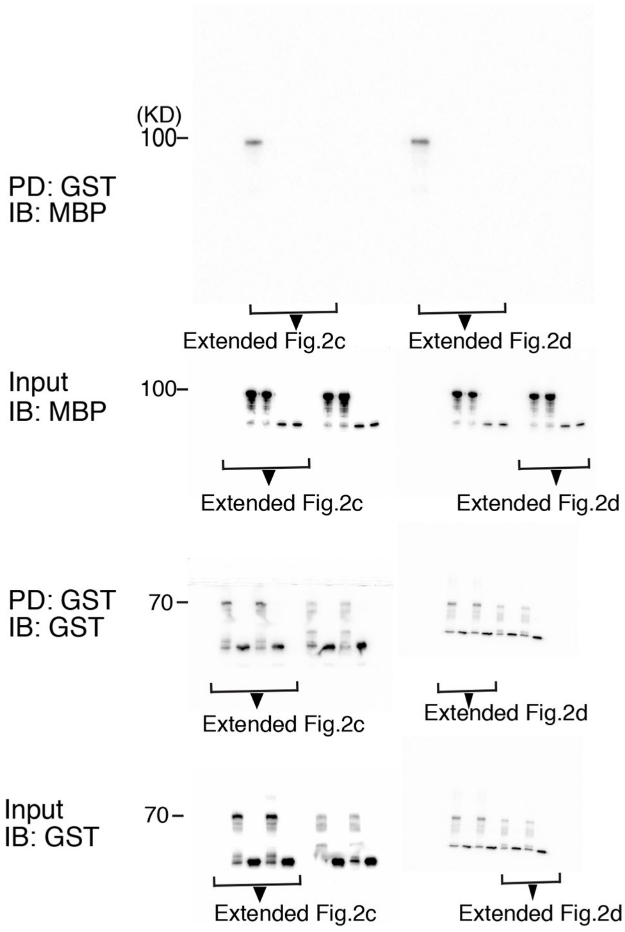

Extended Fig. 2f, g

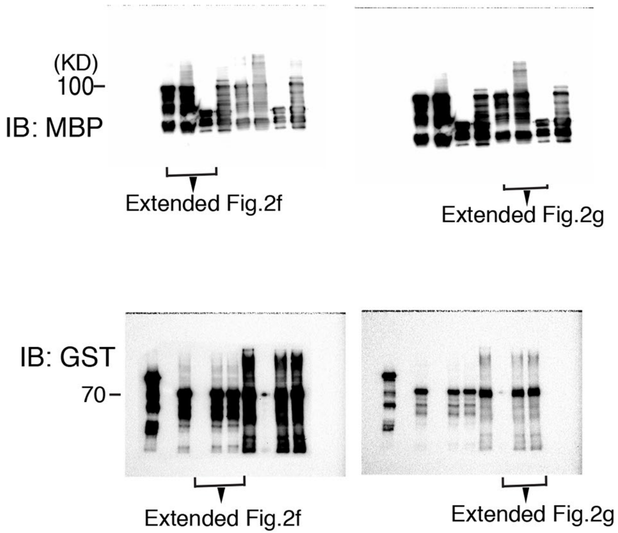

Original Uncropped Scanned Images of blots and gels

Extended Fig. 3b

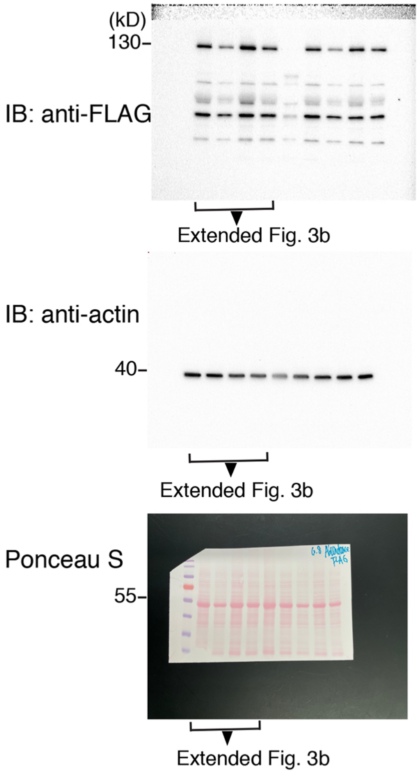

Extended Fig. 3e

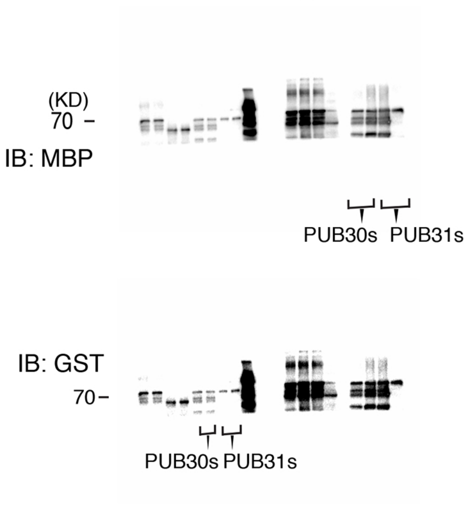

Extended Fig. 3f

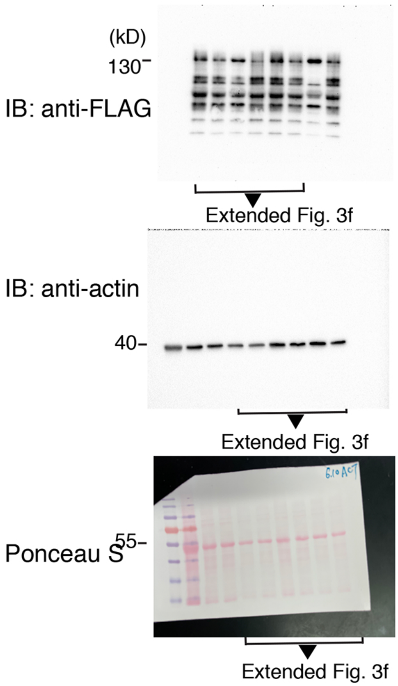

Extended Fig. 3g

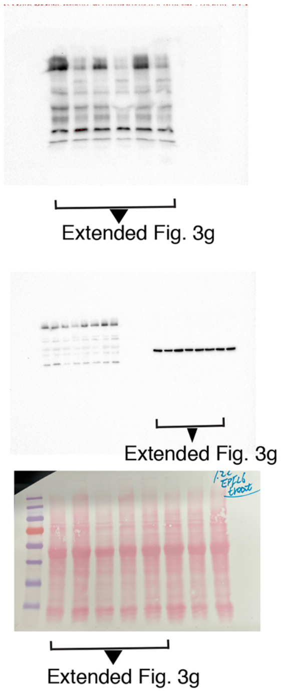

Original Uncropped Scanned Images of blots and gels

Extended Fig. 5a, b

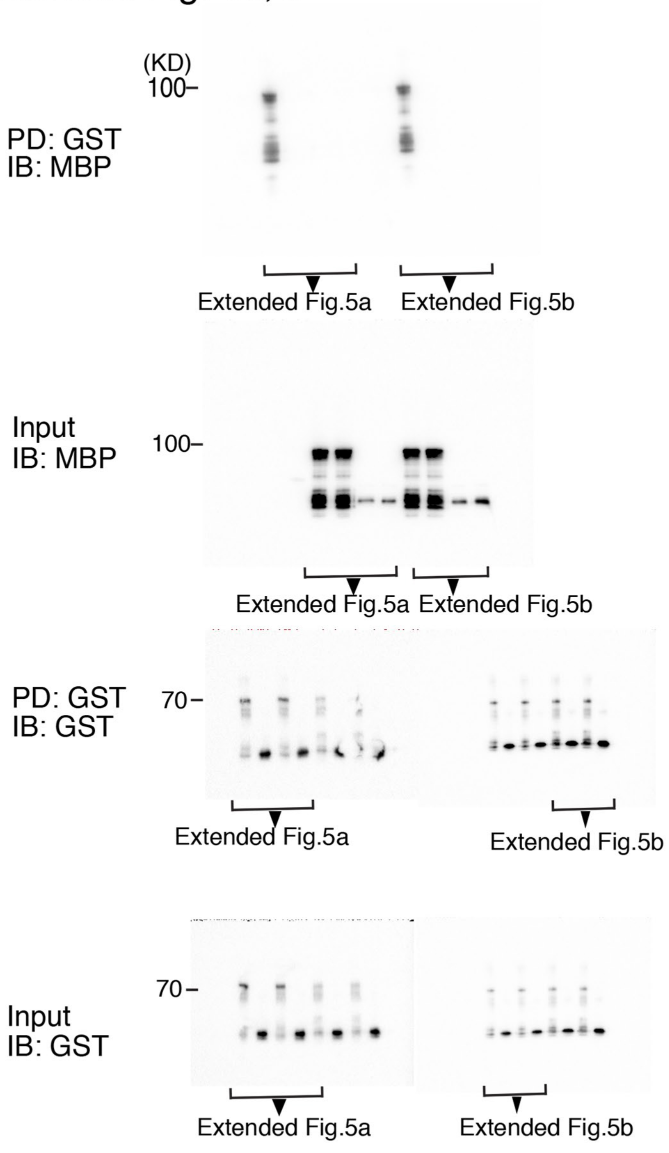

Extended Fig. 5c

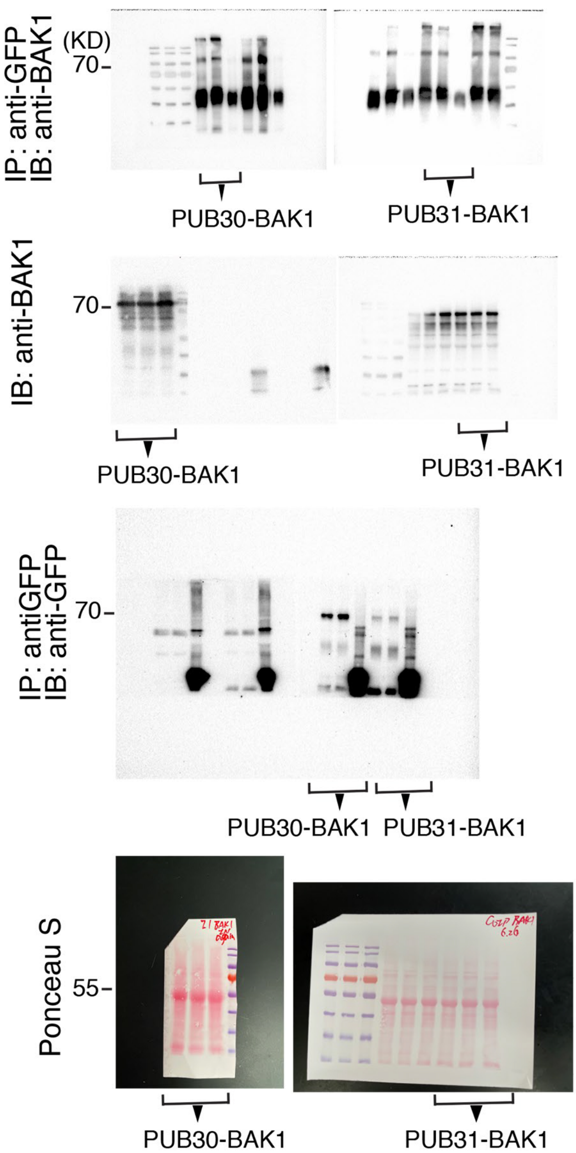

Original Uncropped Scanned Images of blots and gels

Extended Fig. 6a

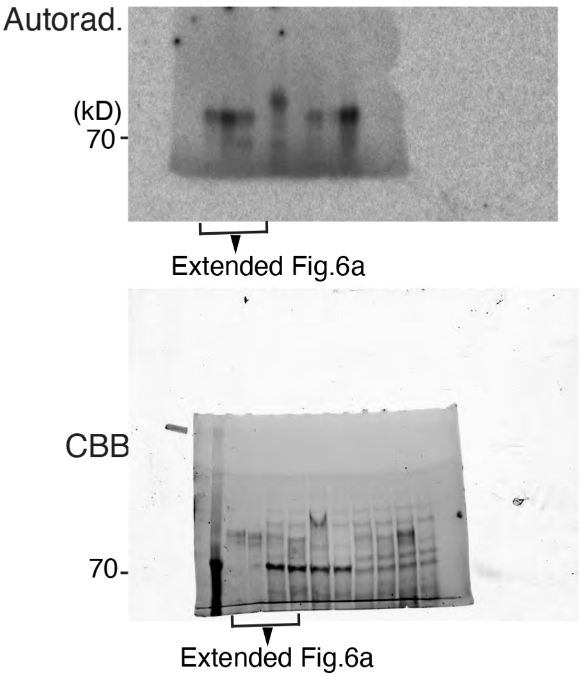

Extended Fig. 6b

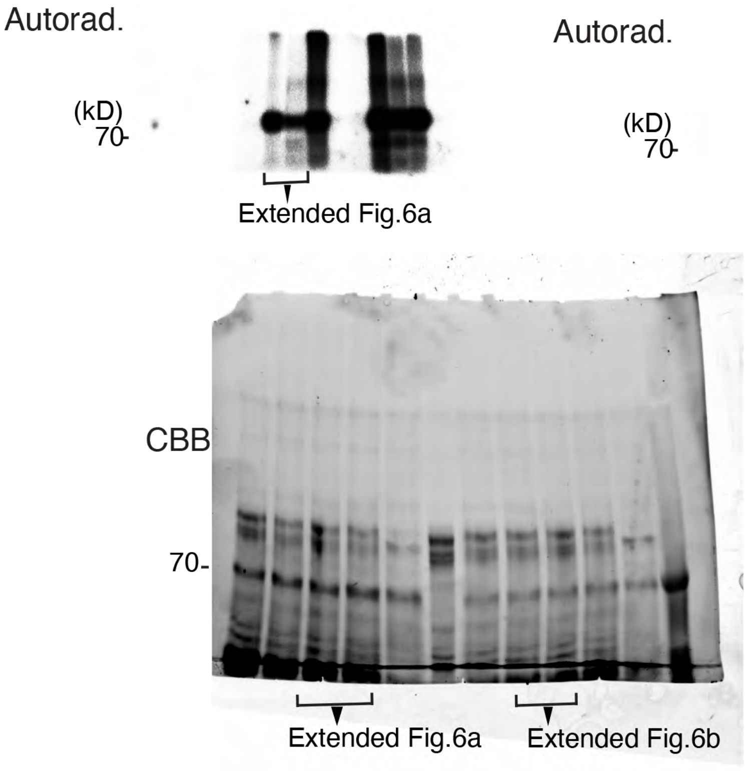

Extended Fig. 6c

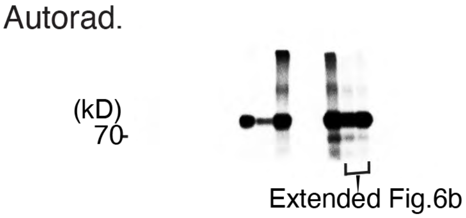

Extended Fig. 6e

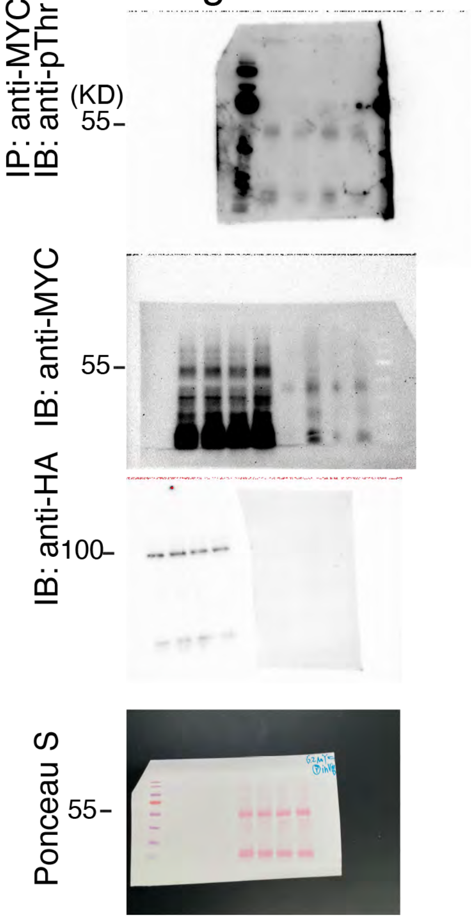

Original Uncropped Scanned Images of blots and gels

Extended Fig. 7a

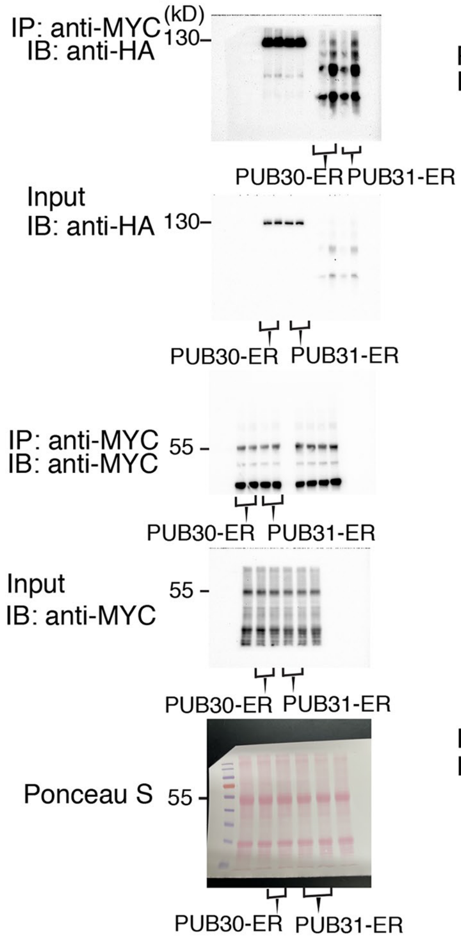

Extended Fig. 7b

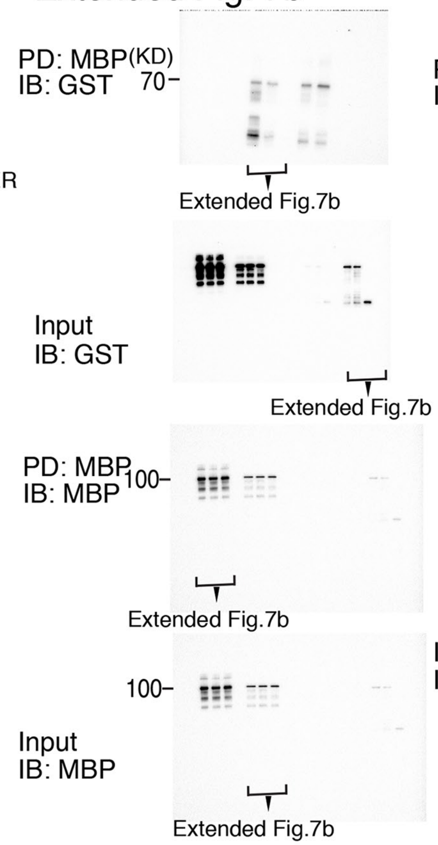

Extended Fig. 7c

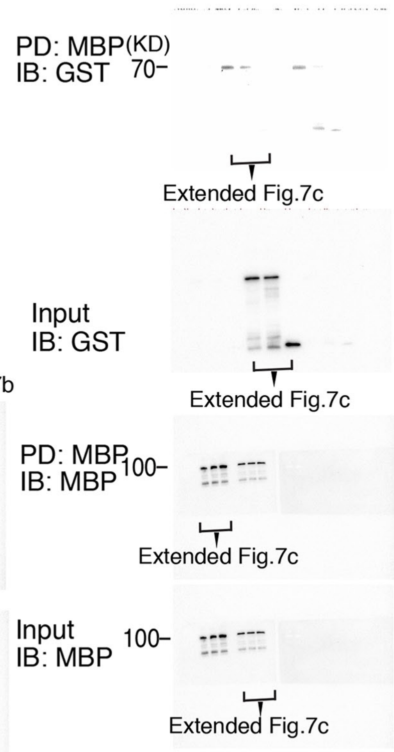

Extended Fig. 7f,g

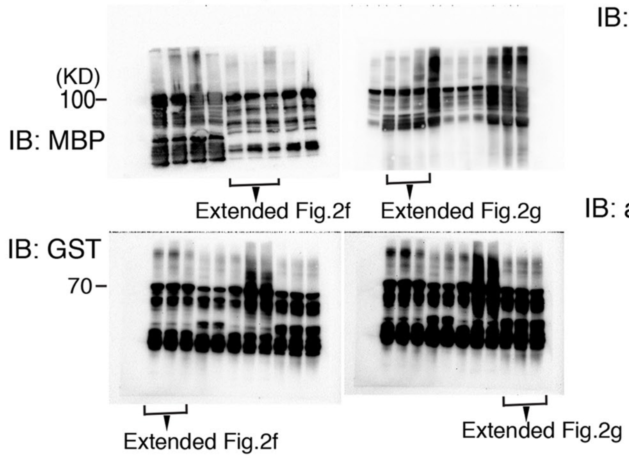

Extended Fig. 7h, i

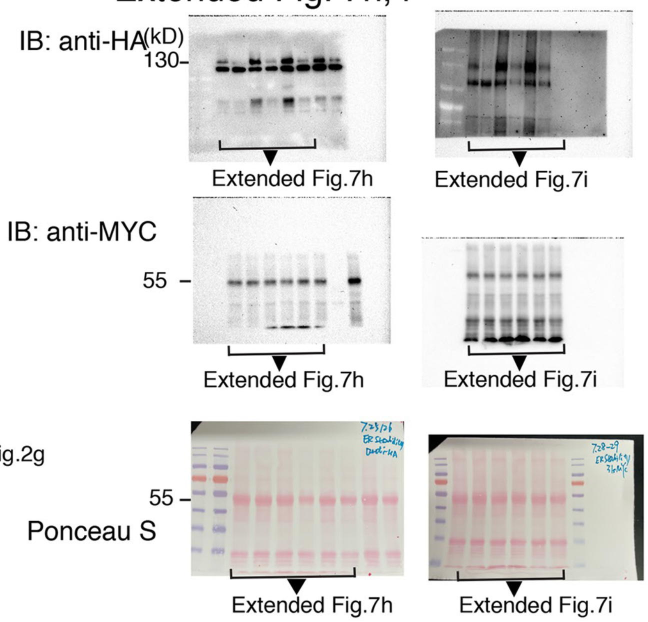

Original Uncropped Scanned Images of blots and gels

Extended Fig. 8a

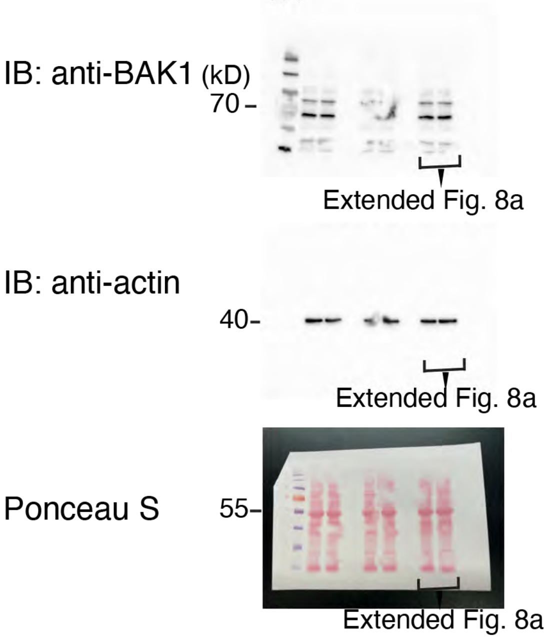

Extended Fig. 8b, c

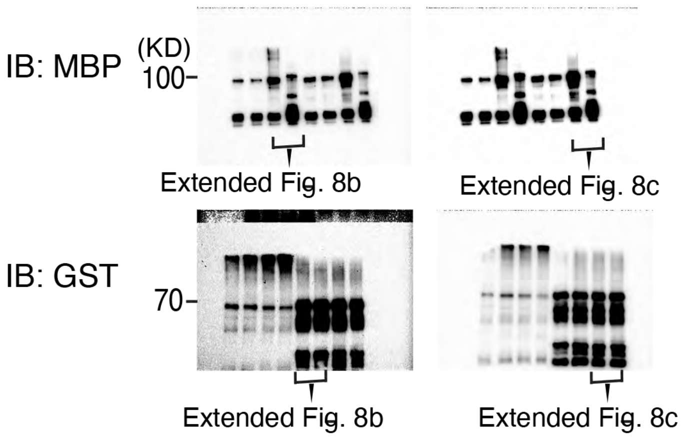

Extended Fig. 8d, e

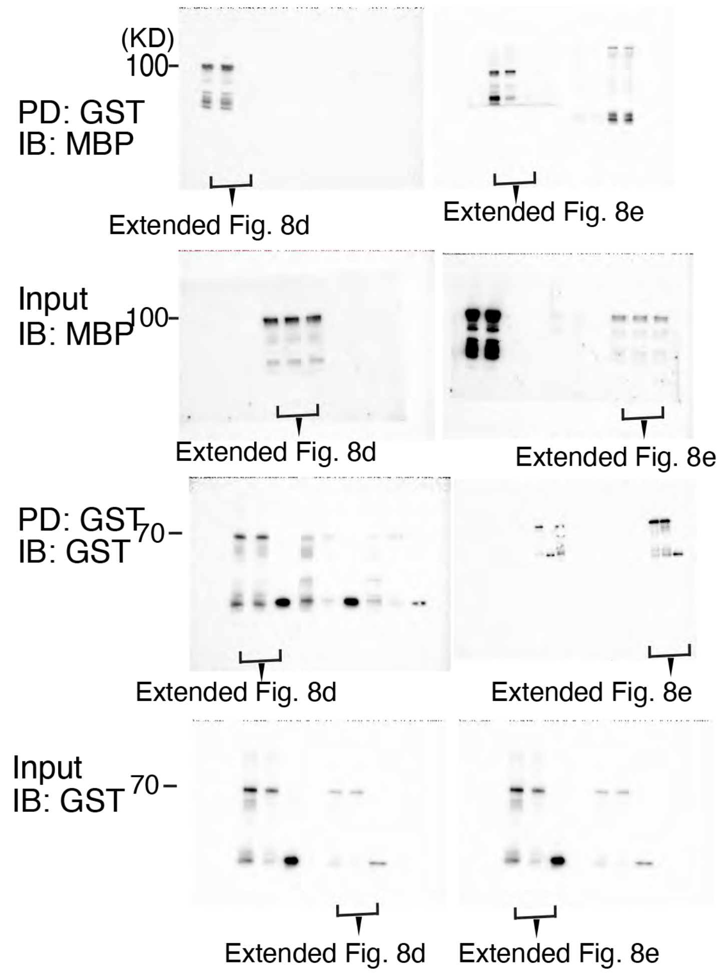

Extended Fig. 8f, g

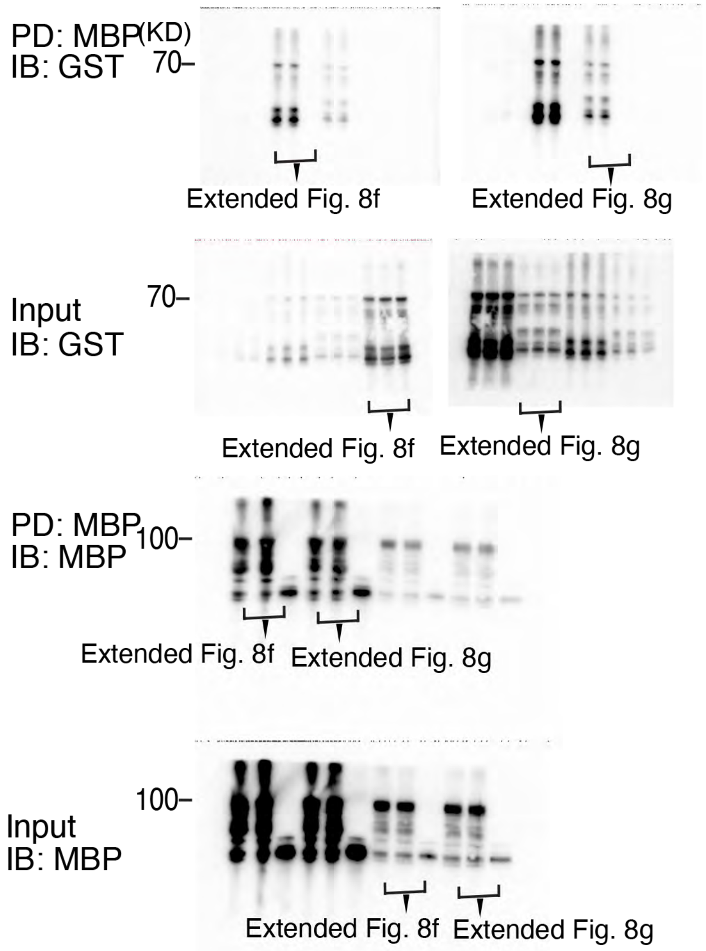

Original Uncropped Scanned Images of blots and gels

Extended Fig. 9a

Extended Fig. 9b

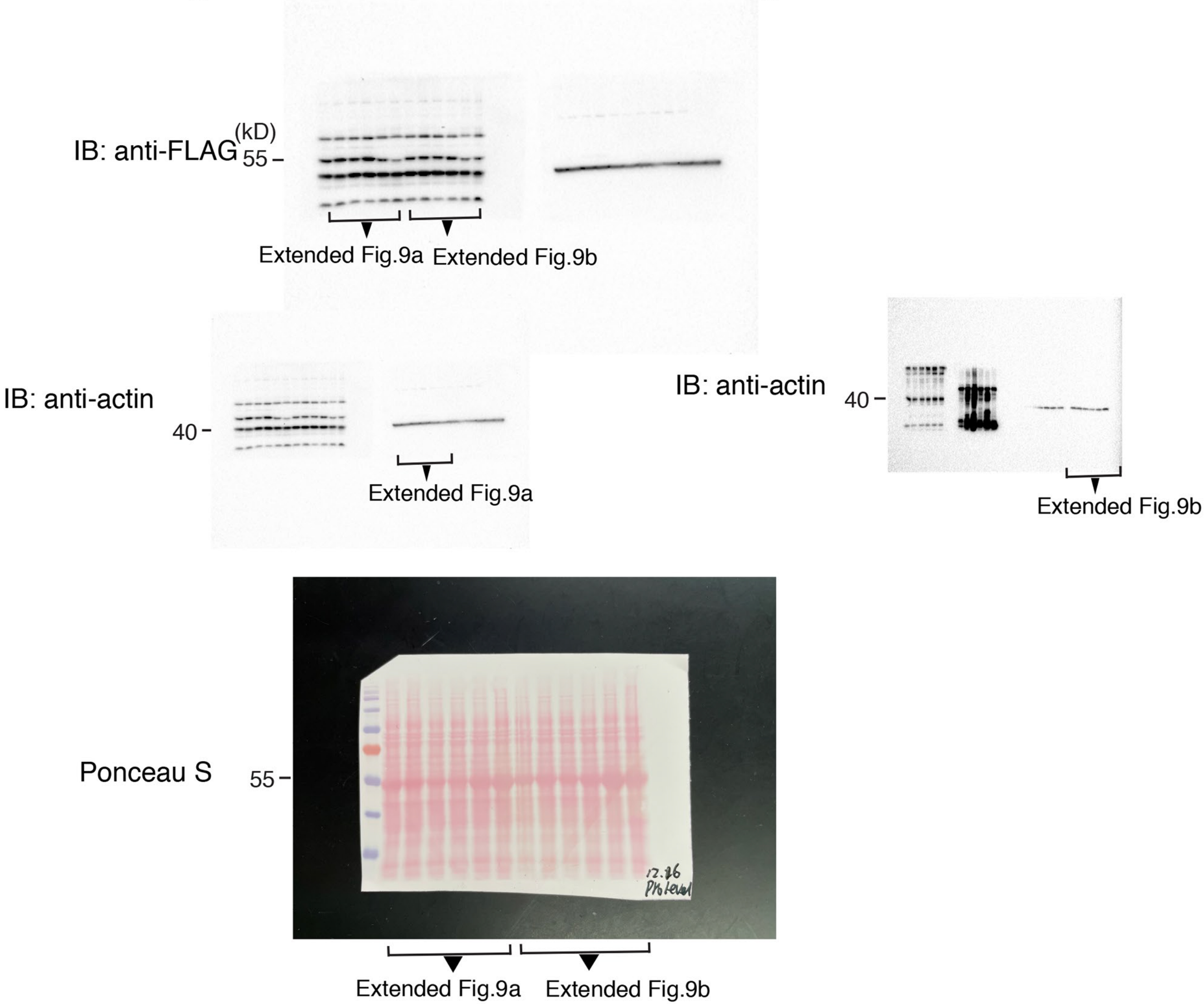

Supplement: Source Data Fig. 1 — All uncropped gel and blot images for all figures. [file 41477_2022_1303_MOESM5_ESM.pdf]
